# Clinical and CT Features of Subsolid Pulmonary Nodules With Interval Growth: A Systematic Review and Meta-Analysis

**DOI:** 10.3389/fonc.2022.929174

**Published:** 2022-07-04

**Authors:** Xin Liang, Mengwen Liu, Meng Li, Li Zhang

**Affiliations:** ^1^ Medical Statistics Office, National Cancer Center/National Clinical Research Center for Cancer/Cancer Hospital, Chinese Academy of Medical Sciences and Peking Union Medical College, Beijing, China; ^2^ Department of Diagnostic Radiology, National Cancer Center/National Clinical Research Center for Cancer/Cancer Hospital, Chinese Academy of Medical Sciences and Peking Union Medical College, Beijing, China

**Keywords:** subsolid nodule, clinical features, CT features, interval growth, meta-analysis

## Abstract

**Background:**

Establishing risk-based follow-up management strategies is crucial to the surveillance of subsolid pulmonary nodules (SSNs). However, the risk factors for SSN growth are not currently clear. This study aimed to perform a systematic review and meta-analysis to identify clinical and CT features correlated with SSN growth.

**Methods:**

Relevant studies were retrieved from Web of Science, PubMed, Cochrane Library, and EMBASE. The correlations of clinical and CT features with SSN growth were pooled using a random-effects model or fixed-effects model depending on heterogeneity, which was examined by the *Q* test and *I^2^* test. Pooled odds ratio (OR) or pooled standardized mean differences (SMD) based on univariate analyses were calculated to assess the correlation of clinical and CT features with SSN growth. Pooled ORs based on multivariate analyses were calculated to find out independent risk factors to SSN growth. Subgroup meta-analysis was performed based on nodule consistency (pure ground-glass nodule (pGGN) and part-solid nodule (PSN). Publication bias was examined using funnel plots.

**Results:**

Nineteen original studies were included, consisting of 2444 patients and 3012 SSNs. The median/mean follow-up duration of these studies ranged from 24.2 months to 112 months. Significant correlations were observed between SSN growth and eighteen features. Male sex, history of lung cancer, nodule size > 10 mm, nodule consistency, and age > 65 years were identified as independent risk factors for SSN growth based on multivariate analyses results. Eight features, including male sex, smoking history, nodule size > 10 mm, larger nodule size, air bronchogram, higher mean CT attenuation, well-defined border, and lobulated margin were detected to be significantly correlated with pGGNs growth. Smoking history showed no significant correlation with pGGN growth based on the multivariate analysis results.

**Conclusions:**

Eighteen clinical and CT features were identified to be correlated with SSN growth, among which male sex, history of lung cancer, nodule size > 10 mm, nodule consistency and age > 65 years were independent risk factors while history of lung cancer was not correlated with pGGN growth. These factors should be considered when making risk-based follow-up plans for SSN patients.

## Introduction

Subsolid pulmonary nodules (SSNs) refer to both part-solid nodules (PSNs) and pure ground-glass nodules (pGGNs) (1), and they are defined as nodules that contain components higher than normal lung tissue but less opaque than consolidated bronchovascular margins (2). The widespread availability of high-resolution computed tomography (CT) and the promotion of low-dose chest CT (LDCT) screening programs have increased the detection rate of SSN. Especially because of the COVID-19 epidemic, people are actively undergoing CT scans, so the probability of finding SSNs in the lungs has greatly increased.

The majority (60%-90%) of persistent SSNs have a more indolent clinical course than solid nodules during 5 to 10 years of observation (3–6), and these nodules often represent precursors of invasive adenocarcinoma. A prospective study suggested that SSN growth often indicated a higher risk of invasive adenocarcinoma (7). For pGGNs, the transition to mixed GGNs (solid component within the ground-glass nodule by thin-section CT at a lung window setting) indicates more rapid growth (8). Moreover, it was reported in two studies that 2% and 13% of SSNs showed growth after 5 years of stability (6, 9). The complex growth characteristics and potential malignant properties of SSNs lead to challenges in clinical management. The current guidelines for SSNs take nodule growth as the basis to adjust the follow-up plan and recommend definitive therapy (10). Although there is no consensus on the duration and frequency of SSN follow-up in the guidelines, the 2017 Fleischner Society guidelines, the American College of Chest Physicians guideline (ACCP) and the National Comprehensive Cancer Network (NCCN) all recommend further evaluation and/or consideration of resection if solid component(s) or growth develops in SSNs (11, 12). Considering the risk of a missed diagnosis of lung cancer and worse prognosis, many patients with persistent SSN(s) may switch to more frequent CT surveillance or definitive treatment. This leads to more overexamination and overtreatment in clinical practice. If we can predict whether a nodule will grow, we can adopt different follow-up schemes for different patients to ease their anxiety and solve these problems.

CT surveillance is the sole effective approach for evaluating SSN growth at present. Although studies have revealed that long-term surveillance of SSN(s) with LDCT is a safe strategy, repeated CT scans over several years have nonnegligible consequences, such as anxiety, radiation exposure, false-positive results and unnecessary costs (13). Therefore, risk-based follow-up management for these patients is greatly desired. Several studies have shown that nodule size and history of lung cancer are important risk factors for SSN growth (14, 15). Other studies have shown that lobular margins and a bubble-like appearance are correlated with the growth of SSN (16). Due to the lack of large-sample data comparisons and analyses of clinical and CT features of SSN growth, we retrieved relevant studies up to December 2021 and carried out a meta-analysis, which aimed to clarify the risk factors correlated with SSN growth and provide information for establishing risk-based follow-up strategy for SSN(s) patients.

## Methods

This meta-analysis was carried out in accordance with the Preferred Reporting Items for Systematic Reviews and Meta-Analyses guidelines (17, 18). The primary procedures are outlined in the following sections.

### Literature Search

We performed a systematic literature search of Web of Science, PubMed, Cochrane Library and EMBASE up to December 31, 2021. The search terms “non-solid nodule”, “part-solid nodule”, “subsolid nodule” and their synonyms combined with “growth” or “follow-up” were used without language restriction, and medical subject headings (MeSH) were applied if available. The reference lists of the retrieved articles and review articles were manually searched for other relevant studies. Two authors (L.Z. and M.W.L.) independently performed the search and reviewed all identified publications for inclusion using predetermined criteria.

### Inclusion Criteria

Studies were included when they met the following criteria: (a) studies published in English or Chinese; (b) the cases included in the studies were of SSNs; (c) clinical or CT features were analyzed in the studies; and (d) nodule growth was defined as the whole nodule growing by > 2 mm in diameter, the emergence of a solid component in a pGGN or the solid area growing by > 2 mm in diameter in a PSN. Reports of lectures, conference papers, and reviews were excluded.

### Data Extraction and Quality Assessment

For each eligible study, two authors independently extracted the following data: (a) general information of the studies, (b) mean value and standard deviation of numerical clinical and CT features included in univariate analysis, (c) number of negative and positive cases for categorical clinical and CT features included in univariate analysis, and (d) odds ratio (OR) value with 95% confidence interval of clinical and CT features in multiple logistic regression model. The Newcastle–Ottawa Scale (NOS) was used to assess the methodological quality of the included studies (19).

### Data Analysis

In the univariate analyses, pooled ORs and pooled standardized mean differences (SMDs) were used to detect the strength of each correlation between binary and continuous features and SSN growth, respectively. To facilitate the analyses, we converted ordered categorical variables in some studies into binary variables. Then, we used Pearson’s chi-square test or Fisher’s exact test to detect if there was a significant difference in the fourfold table for each study and pooled the corresponding ORs. We also estimated the means and standard deviations based on the corresponding medians, ranges and sample sizes for continuous variables whose means and standard deviations were absent (20). Then, we used Student’s t test to detect if there was a significant difference for each study and pooled the corresponding SMDs. In the multivariate analyses, pooled adjusted ORs obtained from multiple logistic regression models were used to assess the strength of each correlation of a CT or clinical feature with nodule growth. *P-*values < 0.05 was considered statistically significant. Statistical heterogeneity was determined using the *Q* test and *I^2^* test (21). If *P* < 0.1 or *I^2^* > 50%, the random-effects model (DerSimonian–Laird model) was used. Otherwise, the fixed-effects model (Mantel–Haenszel model/inverse variance model) was used. Subgroup meta-analysis was performed based on nodule consistency (pGGN and PSN). Publication bias was evaluated by Begg’s funnel plot. *P*-values ≥ 0.05 was considered to indicate that no publication bias existed (22). Statistical analyses were performed with R version 4.0.5 and the Meta package.

## Results

### Study Selection

**Figure 1:**
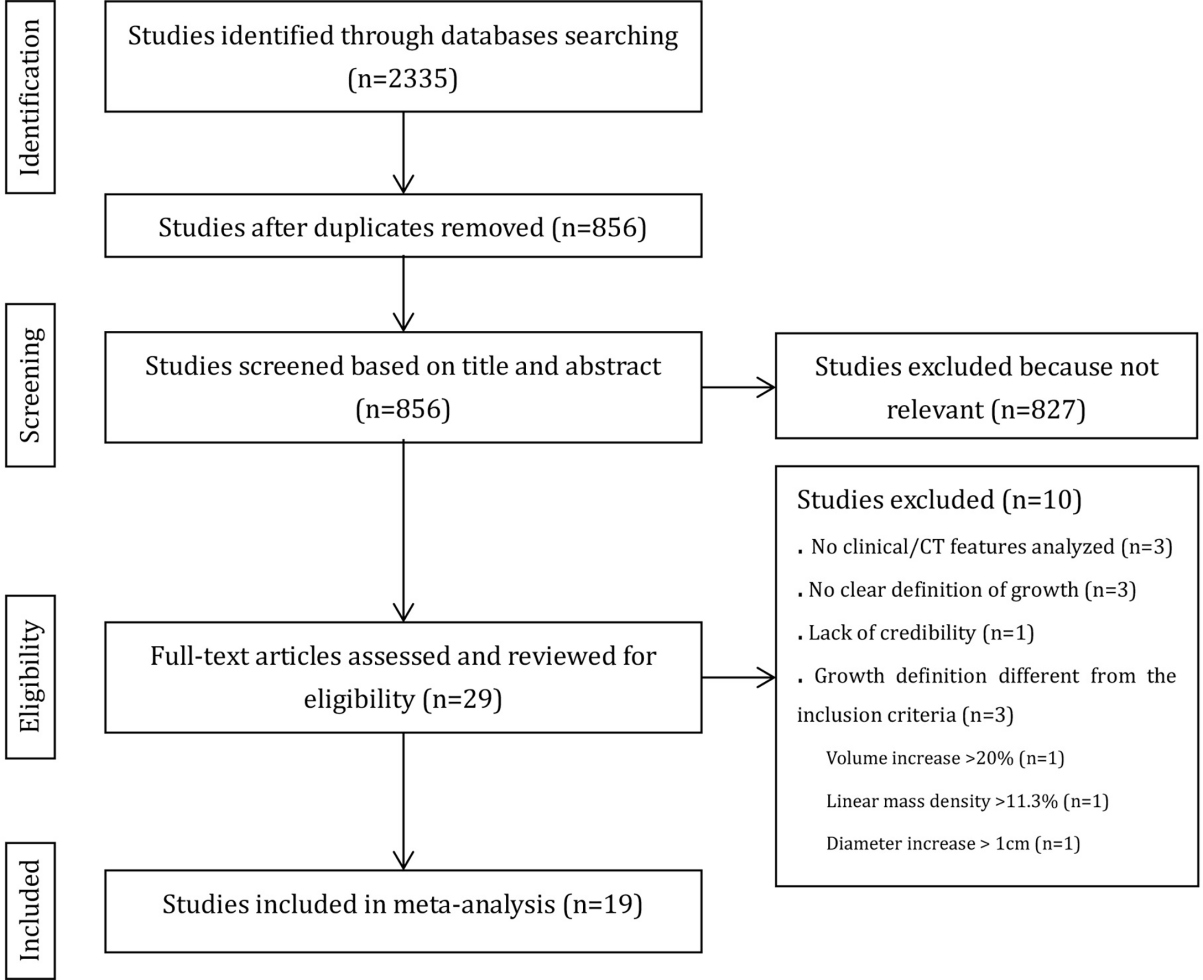
Flow chart shows summary of the literature review process.

**Figure 1** provides an overview of the literature search and study selection process. Nineteen original studies (5, 6, 8, 9, 14–16, 23–34) were retrieved from 827 potential publications that assessed the relationship between SSN growth and CT or clinical features.

### Study Characteristics and Quality Assessment

All included studies were case–control studies. The group with nodule growth was the case group, and the group without nodule growth was the control group. Among the 19 included studies, 2 analyzed SSNs after 5 years of stability, 2 analyzed SSNs after 3 years of stability, and the remaining analyzed SSNs at baseline. The characteristics of the included studies are shown in **Table 1**. In total, 2444 patients with 3012 SSNs were included. The frequency of SSN growth ranged from 2.13% to 51.61% on a per-nodule basis.

**Table 1:** Study characteristics.

First author	Year	Country/Region	Data period	Follow-up duration (months)	Total number (patients/nodules)	Nodule consistency	Number of nodules (pGGN/PSN)	Analyzed by nodule consistentcy	Number of CT Detector Rows	CT Scan DOSE	Slice thickness (mm)	Definition of nodule growth^†^
Haruhisa Matsuguma et al. (15)	2013	Japan	Jan 2000 - Jun 2008	29 (1-136)	174/174	SSN	98/76	Yes	4, 64	Standard-dose	1 or 0.5	a, b, c
Takashi Eguchi et al. (24)	2014	Japan	Sep 1998 - Sep 2013	57 (24.1-113.6)	124/124	pGGN	124/0	No	NA	Low-dose or standard-dose	1.25	a, b
Shotaro Takahashi et al. (16)	2012	Japan	Apr 1999 - Jun 2010	66 ± 25	111/150	pGGN	150/0	No	4, 64	Standard-dose	2	a
Hyun Woo Lee et al. (9)	2019	South Korea	Jan 2003 - Dec 2017	136 (120-179)	160/208	SSN	162/46	No	16, 256	Low-dose	1 or 2	a
Jaeyoung Cho et al. (28)	2016	South Korea	May 2003 - Jun 2015	77.5 (38.1-117.1)	218/453	SSN	438/15	No	64, 256	NA	1 to 3	a, b, c
Yuki Sato et al. (31)	2017	Japan	Apr 2008 - Dec 2014	44 (24.1-87.0)	187/187	SSN	134/53	No	NA	NA	0.625 to 2	a, b, c
Boksoon Chang et al. (14)	2013	South Korea	Jun 1997 - Sep 2006	59 (25-140)	89/122	pGGN	122/0	No	64	Low-dose	1 or 5	a
Jong Hyuk Lee et al. (29)	2016	South Korea	May 2005 - Feb 2013	days:849 (90-2900)	213/213	SSN	136/77	Yes	16, 64	Low-dose or standard-dose	≤ 1.25	a, b, c
Masaya Tamura et al. (26)	2014	Japan	Oct 2008 - Oct 2012	26.1 ± 4.6	53/63	pGGN	63/0	No	NA	NA	2	a, b
Miyako Hiramatsu et al. (23)	2008	Japan	1999-2006	days: 1048 (177-3269)	125/125	SSN	95/30	No	NA	Standard-dose	1.25 or 2	a, b, c
So Hyeon Bak et al. (27)	2016	South Korea	Jan 2004 - Jan 2014	24.2 ± 16.9 (2.2-64.9)	49/54	pGGN	54/0	No	64	Standard-dose	2 to 2.5	a, b
Zhe Shi et al. (34)	2019	China	Jan 2011 - Dec 2012	52 (32-69)	59/101	pGGN	101/0	No	64	Standard-dose	1	a, b
Xianqun Xu et al. (33)	2017	China	Jan 2010 - May 2016	NA	69/69	SSN	NA	No	16	Standard-dose	1	a
Yoshihisa Kobayashi et al. (25)	2014	Japan	Jan 1999 - Feb 2013	NA	67/120	SSN	NA	No	NA	NA	NA	a
Sei Won Lee et al. (8)	2013	South Korea	Apr 2004 - Jul 2011	48 (24-99)	114/175	SSN	143/32	No	64, 256	Standard-dose	3 or 1	a
Wu Fang et al. (30)	2016	China	Jun 2008 - Apr 2015	NA	100/108	pGGN	108/0	No	256	Standard-dose	1.5	a, b
En-Kuei Tang et al. (5)	2019	Taiwan	Jan 2002 - Aug 2016	42.84 ± 35.16	128/128	SSN	93/35	No	16, 64, 256	NA	1 to 2.5	a, b, c
Bixiong Wang et al. (32)	2017	China	Feb 2009-2016	37 (24-81)	169/203	SSN	189/14	No	40	Standard-dose	5 and 1	a
Jong Hyuk Lee et al. (6)	2020	Korea	Jan 2002 - Dec2018	112 (84-208)	235/235	SSN	212/24	No	16, 64	Standard-dose	≤1.5	a, b, c

^†^ a, the whole nodule grew by ≥ 2 mm in diameter; b, emergence of a new solid component; c, the solid area grew by > 2 mm in diameter in part-solid nodules. pGGN, pure ground-glass nodule; PSN, part-solid nodule; SSN, subsolid nodule.

According to the NOS, 14 studies (74%) were high quality (more than five stars), and the other 5 (26%) were low quality (**Supplementary Appendix, Part 1, Table S1**).

### Categorization of Clinical and CT Features

Eighty-six descriptions were used to describe CT or clinical features in the 19 studies. One CT feature (emergence of a solid component) was removed because it is one of the criteria for SSN growth. After merging and subsuming similar descriptions that referred to the same CT findings as a single CT characteristic, 74 features remained. Among them, 11 features were investigated in both one single study for multivariate analysis and more than one study for univariate analysis, 47 features were only investigated in one single study, and 16 features were only investigated in more than one study. Finally, 27 clinical and CT features were included in the meta-analysis, and 58 clinical and CT features which investigated in only one study were extracted from their original studies (5, 8, 9, 16, 23, 24, 27, 28, 32–36) and summarized in **Supplementary Appendix** (**Part 1, Table S2**). Among the 27 features included in the meta-analysis, age was analyzed in three ways: as a continuous variable, a binary variables with a threshold of 65 years, and a binary variables with a threshold of 60 years; nodule size was analyzed in two ways: as a continuous variable, and a binary variable with a threshold of 10 mm. The process of categorizing the clinical and CT features is shown in **Supplementary Appendix** (**Part 1, Table S3**).

### Features Correlated With SSN Growth

Twenty-seven clinical and CT features were included in the meta-analysis. Eighteen features, including male sex, history of lung cancer, smoking history, nodule size > 10 mm, larger nodule size, older age, nodule consistency, bubble-like appearance, air bronchogram, spiculated margin, higher mean CT attenuation, well-defined border, lesion below major fissure, larger volume, larger solid component, lobulated margin, higher STD CT attenuation and higher max CT attenuation, were detected to be significantly correlated with SSN growth, while 9 features, including multiple nodules, longer follow-up duration, age > 60 years, age > 65 years, emphysema, nodule shape, peripheral distribution, pleural/fissure retraction and larger mass, showed no significant correlation with SSN growth. Among the 27 CT and clinical features, 13 features, including male sex, number of nodules, history of lung cancer, smoking history, nodule > 10 mm, nodule size, age (years), follow-up duration, nodule consistency, bubble-like appearance, air bronchogram, spiculated margin and mean CT attenuation, were investigated in five or more studies. The pooled OR/SMD of these features are summarized in **Table 2** and forest plots are shown in **Supplementary Appendix** (**Part 1, Figure S1**).

**Table 2:** Clinical and CT features included in the meta-analysis in SSN.

Features	Studies (patients/nodules)	Test of Correlation			Test of Heterogeneity	
		Pooled OR or SMD	95% CI	*P* Value	*I^2^* (%)	*P* Value
Sex (Male)	16 (1846/2400) ^†^	1.469	1.066-2.026	0.019	42.50	0.037
No. of nodules (Multiple)	16 (1877/2439) ^†^	0.976	0.768-1.241	0.843	0.00	0.693
History of lung cancer (Yes)	12 (1501/1980) ^†^	1.738	1.098-2.750	0.018	54.70	0.012
Smoking history (Yes)	12 (1424/1878) ^†^	1.692	1.137-2.520	0.010	39.40	0.078
Nodule size (> 10 mm)	12 (1466/1978) ^†^	6.386	3.514-11.605	< 0.001	65.60	0.001
Nodule size, mm	10 (1107/1517)	0.678	0.310-1.046	< 0.001	83.40	< 0.001
Age, years	8 (958/1355)	0.305	0.089-0.521	0.006	42.20	0.097
Follow-up duration, months	8 (784/936)	0.077	-0.613-0.767	0.827	94.20	< 0.001
Nodule consistency (PSN)	8 (1167/1597) ^†^	3.682	2.655-5.107	< 0.001	22.80	0.248
Bubble like appearance (Yes)	6 (792/1216)	3.938	1.214-12.772	0.022	72.20	0.003
Air bronchogram (Yes)	5 (824/1154)	4.858	2.593-9.101	< 0.001	20.30	0.285
Spiculated margin (Yes)	5 (824/1154)	10.786	1.006-115.624	0.049	63.70	0.041
Mean of CT attenuation, HU	5 (405/465)	1.952	0.780-3.125	0.001	95.40	< 0.001
Age (> 60 years)	4 (299/385) ^†^	1.578	0.969-2.570	0.067	37.30	0.188
Well-defined border (Yes)	4 (353/443)	0.544	0.301-0.983	0.044	0.00	0.652
Age (> 65 years)	3 (354/425)	1.738	0.792-3.812	0.168	62.70	0.068
Emphysema (Yes)	3 (506/593)	0.607	0.207-1.774	0.361	40.90	0.184
Lesion location (Below major fissure)	3 (374/455)	0.448	0.242-0.832	0.011	0.00	0.761
Nodule shape (Round)	3 (264/321)	0.559	0.263-1.187	0.130	42.70	0.175
Volume, mm3	3 (177/224)	0.988	0.041-1.936	0.041	88.90	< 0.001
Solid part size, mm	2 (288/336)	0.429	0.164-0.695	0.002	0.00	0.575
Lobulated margin (Yes)	2 (200/272)	15.081	3.050-74.575	0.001	0.00	0.650
Peripheral distribution (Yes)	2 (324/357)	3.342	0.432-25.874	0.248	0.00	0.904
Pleural/fissure retraction (Yes)	2 (453/688)	1.963	0.244-15.785	0.526	0.00	0.824
STD of CT attenuation, HU	2 (128/170)	1.067	0.693-1.440	< 0.001	0.00	0.957
Max of CT attenuation, HU	2 (128/170)	1.299	0.574-2.024	< 0.001	72.00	0.059
Mass, mg	2 (108/155)	0.715	-0.986-2.417	0.410	94.50	< 0.001

^†^Total number of patients in Yoshihisa Kobayashi’s study, and Bixiong Wang’s study were not reported; PSN, part solid nodule; OR, odds ratio; SMD, standardized mean difference; CI, confidence interval.

### Independent Risk Factors for SSN Growth

**Figure 2:**
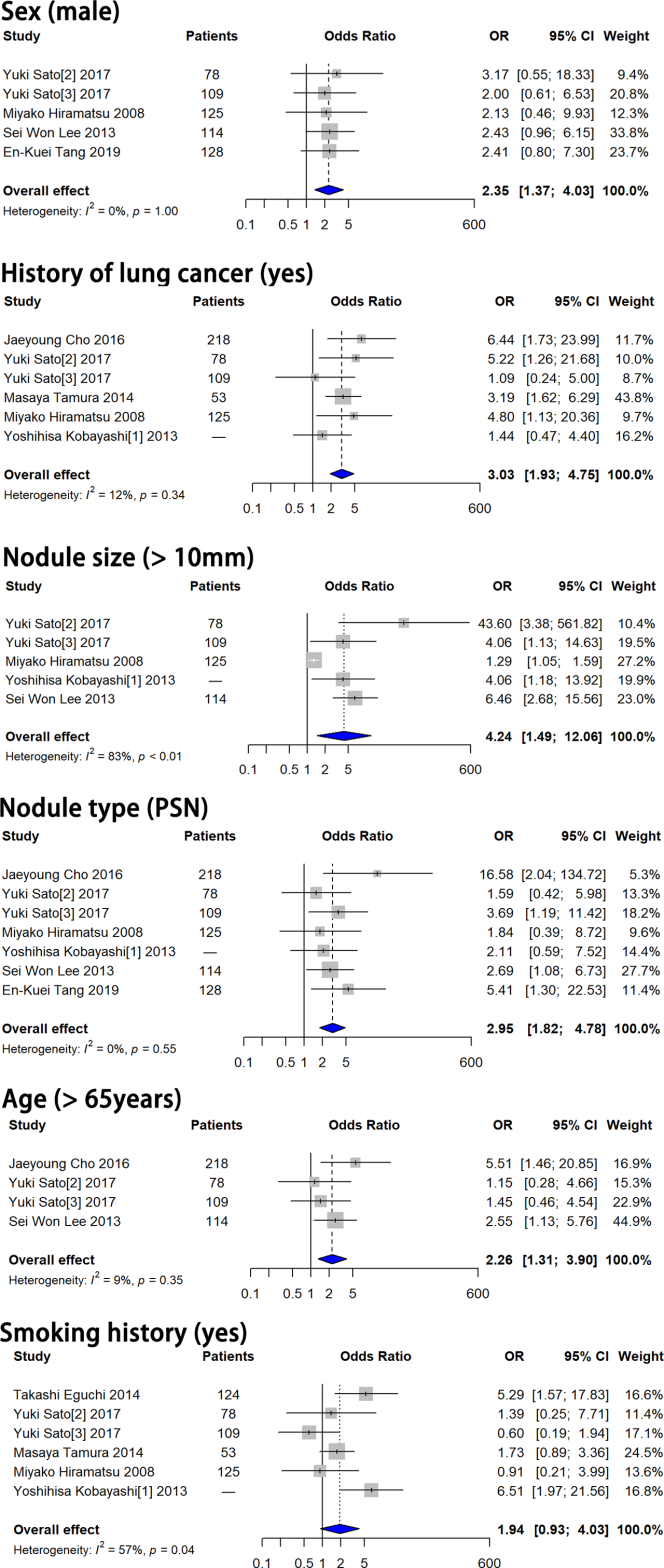
Forest plots showed that male sex, history of lung cancer, nodule size > 10 mm, PSN and age > 65 years were independent risk factors for SSN growth. Smoking history showed no significant correlation with SSN growth. OR, odds ratio; CI, confidence interval; PSN, part-solid nodule.

Pooled ORs of six features, including sex, history of lung cancer, smoking history, nodule size > 10 mm, nodule consistency and age > 65 years, were calculated based on the multivariate analysis results. Forest plots of these six features are shown in **Figure 2**. Sex, history of lung cancer, nodule size > 10 mm, nodule consistency and age > 65 years were proven to be independent risk factors for SSN growth. Male patients showed a 2.351-fold higher probability of SSN growth (pooled OR 2.351, 95% CI 1.370-4.032, *P* = 0.002). Patients with a history of lung cancer had a 3.030-fold higher probability of SSN growth (pooled OR 3.030, 95% CI 1.933-4.749, *P* < 0.001). Patients with a nodule size > 10 mm had a 4.236-fold higher probability of SSN growth (pooled OR 4.236, 95% CI 1.488-12.059, *P* = 0.002). PSNs (nodule consistency) had a 2.951-fold higher probability of SSN growth (pooled OR 2.951, 95% CI 1.821-4.782, *P* < 0.001). Patients aged > 65 years had a 2.260-fold higher probability of SSN growth (pooled OR 2.260, 95% CI 1.308-3.903, *P* = 0.003). Smoking history showed no significant correlation with SSN growth based on the multivariate analysis results (pooled OR 1.941, 95% CI 0.935-4.029, *P* = 0.075).

### Subgroup Analyses on Nodule Consistency

The nodule consistency of the 19 original studies we included was pGGN in 7 studies and SSN in 12 studies. Among the 12 studies taking SSN as research objects, 2 studies analyzed the correlation between features and nodule growth by nodule consistency. In total, 9 studies analyzed the correlation between features and pGGN growth, and 2 studies analyzed the correlation between features and PSN. The number of studies on PSN is too few to perform a meta-analysis. Therefore, we just performed the meta-analysis on pGGN including a total of 16 features based on univariate analysis and 1 feature based on multivariate analysis.

**Figure 3:**
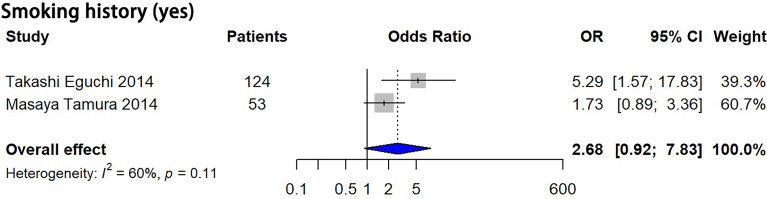
Forest plots showed no significant correlation between smoking history and pGGN growth based on multivariate analysis. OR, odds ratio; CI, confidence interval; pGGN, pure ground glass nodule.

Eight features, including male sex, smoking history, nodule size > 10 mm, larger nodule size, air bronchogram, higher mean CT attenuation, well-defined border, and lobulated margin were detected to be significantly correlated with pGGN growth (*P* = 0.03, 0.003, 0.047, 0.009, 0.001, 0.005, 0.044 and 0.001, respectively), while 8 features, including multiple nodules, history of lung cancer, age, longer follow-up duration, bubble-like appearance, nodule shape, volume and larger mass, showed no significant correlation with pGGN growth (*P* = 0.675, 0.366, 0.071, 0.796, 0.234, 0.130, 0.176 and 0.410, respectively) based on univariate analysis (**Table 3**; **Supplementary Appendix, Part 1, Figure S2**). Smoking history showed no significant correlation with pGGN growth based on the multivariate analysis results (*P* = 0.071, **Figure 3**).

**Table 3:** Clinical and CT features included in the meta-analysis based on univariate analyses in pGGN.

Features	Studies (patients/nodules)	Test of Correlation			Test of Heterogeneity	
		Pooled OR or SMD	95% CI	*P* Value	*I^2^* (%)	*P* Value
Sex (Male)	6 (534/658)	1.615	1.049-2.488	0.030	0.00%	0.614
Number of nodules (Multiple)	7 (634/766)	1.092	0.724-1.648	0.675	0.00%	0.474
History of lung cancer (Yes)	4 (386/435)	1.634	0.564-4.738	0.366	74.90%	0.008
Smoking history (Yes)	5 (423/508)	2.143	1.292-3.554	0.003	0.00%	0.508
Nodule size (> 10 mm)	4 (351/433)	4.975	1.024-24.164	0.047	81.80%	0.001
Nodule size, mm	6 (532/659)	0.847	0.209-1.485	0.009	89.20%	0.000
Age, years	4 (383/497)	0.217	-0.019-0.452	0.071	0.00%	0.905
Follow-up duration, months	6 (496/600)	0.095	-0.623-0.812	0.796	91.60%	0.000
Bubble like appearance (Yes)	3 (300/380)	3.005	0.491-18.379	0.234	80.20%	0.006
Air bronchogram (Yes)	2 (211/258)	4.374	1.764-10.845	0.001	0.00%	0.492
Mean of CT attenuation, HU	4 (336/396)	2.228	0.659-3.798	0.005	96.50%	0.000
Well-defined border (Yes)	4 (353/443)	0.544	0.301-0.983	0.044	0.00%	0.652
Nodule shape (Round)	3 (264/321)	0.559	0.263-1.187	0.130	42.70%	0.175
Volume, mm3	2 (108/155)	1.149	-0.517-2.815	0.176	93.90%	0.000
Lobulated margin (Yes)	2 (200/272)	15.081	3.050-74.575	0.001	0.00%	0.650
Mass, mg	2 (108/155)	0.715	-0.986-2.417	0.410	94.50%	0.000

pGGN, pure ground glass nodule; OR, odds ratio; SMD, standardized mean difference; CI, confidence interval.

### Publication Bias

The funnel plots did not show significant publication bias for the CT and clinical features analyzed based on both the univariate analysis results and multivariate analysis results (*P* > 0.05). (**Supplementary Appendix, Part 1, Figure S3** and **Figure S4**)

## Discussion

In this systematic review and meta-analysis, 18 clinical and CT features were found to be significantly correlated with SSN growth, and 5 features including male sex, history of lung cancer, nodule size > 10 mm, nodule consistency and age > 65 years were identified to be independent risk factors for SSN growth. Eight features, including male sex, smoking history, nodule size > 10 mm, larger nodule size, air bronchogram, higher mean CT attenuation, well-defined border, and lobulated margin were associated with pGGN growth. Among the features associated with SSN growth, 11 features, including sex, history of lung cancer, smoking history, nodule size (> 10 mm), nodule size (mm), age (years), nodule consistency, bubble-like appearance, air bronchogram, spiculated margin and mean CT attenuation, were investigated in 5 or more studies, while the other 7 features were only investigated in 2 to 4 studies. Among the features associated with pGGN growth, 5 features including male sex, number of nodules, smoking history, nodule size (mm) and follow-up duration, were investigated in 5 studies or more studies, while the other 11 features were only investigated in 2 to 4 studies. The real clinical significance of the features investigated in less than 5 studies needs to be further studied due to the small number of studies included.

Studies have proven that PSNs are more aggressive than pGGNs (37, 38). In this meta-analysis, PSNs had a 2.95-fold higher probability of SSN growth than pGGNs, which is consistent with the previous studies. When we analyzed the characteristics of pGGN, we found that history of lung cancer, age, bubble like appearance, and volume, which are significantly correlated SSN growth, are not associated with pGGN growth. Although the including studies for these four features are very limited (only 2 to 4 studies), the results may imply that the features correlated with the growth of the pGGN and the PSN are different. Therefore, to predict the growth of SSN based on risk features, it should be identified whether the nodule is PSN or pGGN at first.

A history of lung cancer showed high correlation with SSN growth, and it forecasted a 3.498-fold higher probability of SSN growth. However, history of lung cancer was not associated with pGGN growth. Multiple studies suggest that lung cancer history is associated with SSN growth (15, 23, 28, 39), but a few studies found that lung cancer history was not associated with pGGN growth (24, 29). A prospective multicenter study concluded that history of lung cancer was not a factor for SSN growth, and 85.1% of nodules included in the study were pGGNs (7). The relationship between the history of lung cancer and the growth of pGGN and PSN needs further study. Shewale et al. (40) retrospectively reviewed 210 patients with GGNs and a history of lung cancer and demonstrated that patients with a lung adenocarcinoma history had a 6.85-fold higher likelihood for SSN growth than patients with other lung cancer subtypes. As we know, among non-small cell lung cancer, adenocarcinoma is a histological subtype prone to epidermal growth factor receptor (EGFR) mutation, and previous studies have shown that the growth of SSN(s) is closely related to gene mutation status. EGFR mutations have been found to be a promoter of GGN growth in both mice and humans (41, 42). This may be one of the reasons why SSNs are more likely to grow in patients with lung cancer, especially those with adenocarcinoma. The correlation between other tumor types and SSN growth needs to be elucidated.

Among the 5 independent risk factors for SSN growth, a nodule size > 10 mm showed the highest correlation with SSN growth, leading to a 4.236-fold higher probability of SSN growth than nodules ≤ 10 mm in size. Nodule size is an important factor in both SSN follow-up guidelines and pulmonary nodule malignancy prediction models. In the Fleischner society, ACCP and NCCN guidelines, the management of SSN(s) differs based on nodule size (11, 12, 43). Nodule size is also a risk factor used in the Mayo model, PanCan model and Vancouver model to estimate the malignancy risk of pulmonary nodules (11, 44). Several studies also use volume to describe the size of nodules. Han et al. (45) found that in lung cancer screening, semiautomatic volume measurements showed higher accuracy than diameter measurements. In this meta-analysis, three original studies were included, and a significant correlation between volume and nodule growth was found (pooled SMD 0.988, 95% CI 0.041-1.936, *P* = 0.041). However, considering the difficulties in identifying the SSN boundary for computer-aided measurements, using diameter to describe the size of the SSN is still a better choice.

In this meta-analysis, we also found another interesting result. The follow-up duration showed no significant correlation with either SSN growth or pGGN growth (*P* = 0.827 and 0.796 respectively). Kobayashi et al. (4) found the tendency to grow was clear within the first 3 years for SSNs. Lee et al. (6) followed SSNs that had been stable for initial 5 years and found that only 5 (2.1%) of nodules grew. The frequency of SSNs increasing in size after prolonged stability is quite small, which is consistent to our results. Based on the above, we speculate that the frequency of CT examination can be reduced for SSN followed up for more than 5 years.

Studies have reported that approximately 37%-70% of SSNs detected on CT screening are transient and resolve spontaneously or with antibiotic therapy within 3 months of the initial examination (46–48). Features associated with transient SSNs include younger age, male sex, peripheral eosinophilia, multiplicity, ill-defined margins, nonspiculated margins, and large solid components (47, 49). Some features, such as ill-defined margins, male sex and large solid components coincide with the features of SSNs growth which may lead to the determination of a transient nodule as a growing nodule. Therefore, we suggest that the prediction of SSN growth should be performed at least 3 months after the initial examination. SSN growth prediction is important not only to make follow-up plan but also to optimize surgical timing. Based on the included studies, we found that only 49.23% stable SSNs were invasive adenocarcinoma (IAC) while 81.01% growth SSNs were IAC, and growth SSNs showed a 4.32-fold higher probability of invasive adenocarcinoma than stable SSNs (**Supplementary Appendix, Part 2**). These results suggest that surgical resection after the growth of SSNs may be more appropriate than upon detection, which needs further study.

Our study had several limitations. First, the study subjects in the included studies were not completely homogenous. Among the 19 included studies, 2 analyzed SSNs after 5 years of stability, 2 analyzed SSNs after 3 years of stability, and the remaining analyzed SSNs at baseline. Fortunately, no significant differences in the included features were detected in publication bias tests. Second, most of the features had no multivariate analysis results in the original studies, and their specific role in SSN growth needs to be further studied. Third, compared with a large number of clinical and CT features, the number of studies included is so small that the meta-analysis of some features may lack credibility. Fourth, the number of studies on PSN is too few to perform a meta-analysis. Nevertheless, to the best of our knowledge, our meta-analysis is the only study to investigate the correlation of clinical and CT features with SSN growth, and this analysis included all available literature.

In conclusion, in this meta-analysis, eighteen clinical and CT features were detected to be significantly correlated with SSN growth, and 5 features including male sex, history of lung cancer, nodule size > 10 mm, nodule consistency and age > 65 years were identified to be independent risk factors for SSN growth. For pGGN, history of lung cancer, older age, bubble-like appearance, and larger volume were not risk factors for growth, although these factors were associated with SSN growth. A risk-based SSN follow-up strategy should consider these factors and nodule consistency, and separate strategies should be planned for each single nodule in patients with multiple SSNs.

## Data Availability Statement

The original contributions presented in the study are included in the article/**Supplementary Material**. Further inquiries can be directed to the corresponding author.

## Author Contributions

(I) Conception and design: All authors; (II) Administrative support: None; (III) Provision of study materials or patients: None; (IV) Collection and assembly of data: LZ, MWL, ML; (V) Data analysis and interpretation: All authors; (VI) Manuscript writing: All authors; (VII) Final approval of manuscript: All authors

## Funding

This work was supported by National Natural Science Foundation of China (81701692); and Beijing Municipal Natural Science Foundation (7184238).

## Conflict of Interest

The authors declare that the research was conducted in the absence of any commercial or financial relationships that could be construed as a potential conflict of interest.

## Publisher’s Note

All claims expressed in this article are solely those of the authors and do not necessarily represent those of their affiliated organizations, or those of the publisher, the editors and the reviewers. Any product that may be evaluated in this article, or claim that may be made by its manufacturer, is not guaranteed or endorsed by the publisher.
