# Supplementary material for: Clinical and CT Features of Subsolid Pulmonary Nodules With Interval Growth: A Systematic Review and Meta-Analysis
Source: Front Oncol. 2022 Jul 4;12:929174. doi: 10.3389/fonc.2022.929174 (PMC9289285; doi:10.3389/fonc.2022.929174)
Supplement: Supplementary file 1 [file DataSheet_1.docx]

Supplementary Appendix

Part 1:

| **Table S1.** Quality assessment of included studies. | | | | | | | | |
| --- | --- | --- | --- | --- | --- | --- | --- | --- |
| Paper number | Selection | | | | Comparability | Exposure | | |
|  | Adequate definition of cases | Representativeness of the cases | Selection of Controls | Definition of Controls | Comparability of cases and controls on the basis of the design or analysis | Ascertainment of exposure | Same method of ascertainment for cases and controls | Non-Response rate |
| 1 (12) | ★ | ★ | ★ | ★ | ★★ | ★ | ★ | ★ |
| 2 (22) | ★ | ★ | ★ | ★ | ★★ | ★ | ★ | ★ |
| 3 (13) |  | ★ |  |  |  | ★ | ★ | ★ |
| 4 (5) | ★ |  | ★ | ★ | ★★ | ★ | ★ | ★ |
| 5 (21) |  |  |  |  | ★★ | ★ | ★ | ★ |
| 6 (26) | ★ | ★ | ★ | ★ | ★★ | ★ | ★ | ★ |
| 7 (3) |  | ★ |  |  |  | ★ | ★ | ★ |
| 8 (25) | ★ | ★ | ★ | ★ | ★★ | ★ | ★ | ★ |
| 9 (31) | ★ | ★ | ★ | ★ | ★★ | ★ | ★ | ★ |
| 10 (23) | ★ | ★ | ★ | ★ | ★★ | ★ | ★ | ★ |
| 11 (20) | ★ |  | ★ | ★ |  | ★ | ★ | ★ |
| 12 (27) | ★ |  | ★ | ★ | ★★ | ★ | ★ | ★ |
| 13 (32) |  |  |  |  |  | ★ | ★ | ★ |
| 14 (24) |  | ★ |  |  | ★★ | ★ | ★ | ★ |
| 15 (4) |  | ★ |  |  | ★★ | ★ | ★ | ★ |
| 16 (31) | ★ |  | ★ | ★ | ★★ | ★ | ★ | ★ |
| 17 (29) | ★ | ★ | ★ | ★ | ★★ | ★ | ★ | ★ |
| 18 (30) |  | ★ |  |  |  | ★ | ★ | ★ |
| 19 (6) | ★ |  | ★ | ★ |  | ★ | ★ | ★ |

| **Table S2.** Clinical or CT features investigated in one single study. | | | | | | | |  |
| --- | --- | --- | --- | --- | --- | --- | --- | --- |
|  | **univariate** | | | | **OR** | | |  |
|  |  |  |  |  |  |  |  |  |
|  |  |  |  |  |  |  |  |  |
|  | study(patients/nodules) | growth | nongrowth | P | study(patients/nodules) | OR (95% CI) | P |  |
| Age,years |  |  |  |  | En-Kuei Tang (128/128) | 1.071(1.018-1.128) | 0.009 |  |
| Age (> 60) |  |  |  |  | Miyako Hiramatsu (125/125) | 0.99(0.93-1.07) | 0.950 |  |
| History of cancer (Yes) | Shotaro Takahashi (111/150) | 14/5 | 107/24 | 0.533 |  |  |  |  |
| History of cancer (excluded lung cancer) (Yes) | Hyun Woo Lee (160/208) | 6/19 | 14/121 | 0.118 |  |  |  |  |
| Smoking history (>= 10 pack-years) | Sei Won Lee (114/175) | 23/22 | 33/97 | 0.002 |  |  |  |  |
| Smoking, pack-years | Hyun Woo Lee (160/208) | 7.5(13.2) | 14.9(23.4) | 0.033 |  |  |  |  |
| Bronchiectasis (Yes) | Hyun Woo Lee (160/208) | 3/22 | 20/115 | 0.954 |  |  |  |  |
| Pulmonary fibrosis (Yes) | Shotaro Takahashi (111/150) | 0/19 | 0/131 | N/A |  |  |  |  |
| Surgical intervention (Yes) | Zhe Shi (59/101) | 5/11 | 3/40 | 0.015 |  |  |  |  |
| WBC | Hyun Woo Lee (160/208) | 5242(1239) | 5741(1460) | 0.080 |  |  |  |  |
| Eosinophil count | Hyun Woo Lee (160/208) | 126(99) | 169(165) | 0.086 |  |  |  |  |
| Neutrophil to lymphocyte ratio | Hyun Woo Lee (160/208) | 1.8(0.7) | 1.8(0.8) | 0.595 |  |  |  |  |
| CRP | Hyun Woo Lee (160/208) | 0.1(0.1) | 0.1(0.2) | 0.674 |  |  |  |  |
| CEA | Hyun Woo Lee (160/208) | 1(1) | 1.2(1.4) | 0.352 |  |  |  |  |
| CA19-9 | Hyun Woo Lee (160/208) | 8.5(9.6) | 6.7(7.8) | 0.390 |  |  |  |  |
| CA125 | Hyun Woo Lee (160/208) | 8.1(7.5) | 8.4(7.1) | 0.899 |  |  |  |  |
| p53 LI prediction | Xianqun Xu (69/69) | 7.8(1.29) | 5.9(1.12) | <0.001 |  |  |  |  |
| Type of relation between lesion and vessel (Type 1 vs. Type 2) | Bixiong Wang (169/203) | 37/24 | 129/13 | <0.001 |  |  |  |  |
| Perifissure distribution (Yes) | Hyun Woo Lee (160/208) | 3/24 | 16/165 | 0.981 |  |  |  |  |
| Follow-up duration, years |  |  |  |  | En-Kuei Tang (128/128) | 1.716(1.358-2.169) | <0.0001 |  |
| Follow-up duration (>=4 years) | Sei Won Lee (114/175) | 25/20 | 65/65 | 0.520 |  |  |  |  |
| Nodule size, mm |  |  |  |  | En-Kuei Tang (128/128) | 0.915(0.832-1.006) | 0.066 |  |
| Nodule size (> 8mm) |  |  |  |  | Jaeyoung Cho (218/453) | 5.74(1.58-20.92) | 0.008 |  |
| No. of nodules (Multiple) |  |  |  |  | Miyako Hiramatsu (125/125) | 0.53(0.14-2.05) | 0.360 |  |
| Solid part size, mm |  |  |  |  | En-Kuei Tang (128/128) | 1.071(0.936-1.224) | 0.318 |  |
| Air bronchogram (Yes) |  |  |  |  | Jaeyoung Cho (218/453) | 5.83(1.41-24.19) | 0.015 |  |
| Bubble like appearance (Yes) |  |  |  |  | Jaeyoung Cho (218/453) | 9.88(0.67-146.14) | 0.096 |  |
| Lesion location (Below major fissure) |  |  |  |  | Miyako Hiramatsu (125/125) | 0.62(0.17-2.29) | 0.470 |  |
| Spiculated margin (Yes) |  |  |  |  | Jaeyoung Cho (218/453) | 4.36(0.18-103.35) | 0.362 |  |
| Pleural tag (Yes) | Shotaro Takahashi (111/150) | 1/18 | 2/129 | 0.336 |  |  |  |  |
| Marginal hyper-attenuation (Yes) | Shotaro Takahashi (111/150) | 0/19 | 12/119 | 0.364 |  |  |  |  |
| Involvement (Present vs. Absent) | Masaya Tamura (53/63) | 15/14 | 15/19 | 0.547 |  |  |  |  |
| Vessel change (Yes) | Wu Fang (100/108) | 8/7 | 24/69 | 0.030 |  |  |  |  |
| Subpleural distribution (Yes) | Hyun Woo Lee (160/208) | 13/14 | 77/104 | 0.734 |  |  |  |  |
| Total number of CT scans | Hyun Woo Lee (160/208) | 8(4 17) | 9(3 17) | 0.605 |  |  |  |  |
| Mean of CT attenuation, HU |  |  |  |  | Takashi Eguchi (124/124) | 1.015(1.01-1.021) | < 0.001 |  |
| Mean of CT attenuation (> -500HU) | Miyako Hiramatsu (125/125) | 16/10 | 42/57 | 0.080 | Miyako Hiramatsu (125/125) | 1(0.99-1.005) | 0.700 |  |
| Mean of CT attenuation(>=-677HU) | Masaya Tamura (53/63) | 18/11 | 7/27 | 0.001 | Masaya Tamura (53/63) | 7.572(2.021-8.102) | 0.002 |  |
| Min of CT attenuation, HU | Zhe Shi (59/101) | 932.8 (30.5) | -906.1 (44.7) | 0.136 |  |  |  |  |
| Density, mg/mm3 | So Hyeon Bak (49/54) | 0.3 (0.1) | 0.4 (0.1) | 0.001 |  |  |  |  |
| 2.5th percentile, HU | So Hyeon Bak (49/54) | -882.1 (50.7) | -884.8 (44.8) | 0.840 |  |  |  |  |
| 10th percentile, HU | Zhe Shi (59/101) | −878 (28.9) | −866.2 (70.1) | 0.184 |  |  |  |  |
| 25th percentile, HU | So Hyeon Bak (49/54) | -761.6 (64) | -744.4 (49.8) | 0.280 |  |  |  |  |
| 50th percentile, HU | So Hyeon Bak (49/54) | -686.4 (87.3) | -655.7 (71.5) | 0.170 |  |  |  |  |
| 75th percentile, HU | So Hyeon Bak (49/54) | -599.9 (115.7) | -551.4 (112.8) | 0.140 |  |  |  |  |
| 90th percentile, HU | Zhe Shi (59/101) | -532 (406) | −716.8 (203.8) | 0.001 |  |  |  |  |
| 97th percentile, HU | So Hyeon Bak (49/54) | -381.9 (188.4) | -393.3 (126.1) | 0.790 |  |  |  |  |
| Slope,2.5th to 97.5th percentiles | So Hyeon Bak (49/54) | 5.5 (15.7) | 5.7 (1.9) | 0.940 |  |  |  |  |
| Surface area, mm^2^ | Zhe Shi (59/101) | 525.5 (238.1) | 227.1 (124.1) | 0.001 |  |  |  |  |
| Sphericity | Zhe Shi (59/101) | 0.84 (0.04) | 0.88 (0.03) | 0.058 |  |  |  |  |
| Compactness | Zhe Shi (59/101) | 0.041 (0.003) | 0.044 (0.002) | 0.084 |  |  |  |  |
| Elongation | Zhe Shi (59/101) | 0.86 (0.05) | 0.88 (0.07) | 0.737 |  |  |  |  |
| Flatness | Zhe Shi (59/101) | 0.73 (0.11) | 0.79 (0.09) | 0.155 |  |  |  |  |
| Median CT attenuation, HU | Zhe Shi (59/101) | -817.6 (81.1) | -815.1 (136.5) | 0.405 |  |  |  |  |
| Skewness | Zhe Shi (59/101) | 2.5 (1.1) | 1.7 (1.1) | 0.906 |  |  |  |  |
| Entropy | Zhe Shi (59/101) | 2.2 (0.8) | 1.6 (0.6) | 0.096 |  |  |  |  |
| Kurtosis | Zhe Shi (59/101) | 13.5 (9.5) | 8.8 (7.7) | 0.130 |  |  |  |  |
| Homogeneity | Zhe Shi (59/101) | 0.32 (0.11) | 0.44(0.12) | 0.670 |  |  |  |  |

| **Table S3. Categorization of clinical and CT features.** | | |
| --- | --- | --- |
| Descriptors | Synonym merge | Number of studies |
| Age (years) | Age (years) | # |
| Age (> 65) | Age (> 65) | > 1 |
| Age (> 60) | Age (> 60) | # |
| Sex (Male VS. Female) | Sex (Male VS. Female) | > 1 |
| History of lung cancer (Yes) | History of lung cancer (Yes) | > 1 |
| History of cancer (Yes) | History of cancer (Yes) | 1 |
| History of cancer (excluded lung cancer) (Yes) | History of cancer (excluded lung cancer) (Yes) | 1 |
| Smoking history (Yes) | **Smoking history (Yes)** | > 1 |
| Smoking history (Never, former, current, unknown) (Yes vs. No) |  |  |
| Smoking history (>= 10 pack-years) | Smoking history (>= 10 Packy) | 1 |
| smoking (pack-years) | smoking (pack-years) | 1 |
| Emphysema (Yes) | Emphysema (Yes) | > 1 |
| Bronchiectasis (Yes) | Bronchiectasis (Yes) | 1 |
| Pulmonary fibrosis (Yes) | Pulmonary fibrosis (Yes) | 1 |
| surgical intervention (Yes) | surgical intervention (Yes) | 1 |
| WBC | WBC | 1 |
| Eosinophil count | Eosinophil count | 1 |
| Neutrophil to lymphocyte ratio | Neutrophil to lymphocyte ratio | 1 |
| CRP | CRP | 1 |
| CEA | CEA | 1 |
| CA19-9 | CA19-9 | 1 |
| CA125 | CA125 | 1 |
| p53 LI prediction | p53 LI prediction | 1 |
| Type of relation between lesion and vessel (Type 1 vs. Type 2) | Type of relation between lesion and vessel (Type 1 vs. Type 2) | 1 |
| Follow-up duration, months | **Follow-up duration, months** | # |
| Follow-up duration, years |  |  |
| Follow-up duration (>=4 years) | Follow-up duration (>=4 years) | 1 |
| Homogeneity | Homogeneity | 1 |
| Nodule size (mm) | Nodule size (mm) | # |
| Air bronchogram (Yes) | Air bronchogram (Yes) | # |
| Emergence of solid part | Emergence of solid part | * |
| Bubble like appearance (Yes) | Bubble like appearance (Yes) | # |
| Nodule size (> 10mm) | **Nodule size (> 10mm)** | > 1 |
| size grouping (> 5, 5-7, 8-9, > 10mm) |  |  |
| Diameter (1, 1.1-2, 2.1-3cm) |  |  |
| size grouping (< 5, 5-7.9, 8-9.9, 10-14.9, >15mm) |  |  |
| Diameter (2; 2-3.9; 4-5.9; 6-7.9; 8-9.9; 10mm) |  |  |
| Nodule size (> 8mm) | Nodule size (> 8mm) | 1 |
| No. of nodules (Multiple VS. Solitary) | **No. of nodules (Multiple)** | # |
| Number of lesions (1,2,3,4) |  |  |
| Solid part size, mm | Solid part size, mm | # |
| existence of solid part (Yes) | **Nodule type (PSN)** | > 1 |
| Solid component (0; 1-25; 26-50) |  |  |
| solid component (0; 1-50) |  |  |
| Lesion location (Below major fissure VS. Above) | **Lesion location (Below major fissure** | # |
| Lesion location (RUL, RML, RLL, LUL, LLL) |  |  |
| Spiculated margin (YES) | **Spiculated margin (YES)** | # |
| Margin (smooth, lobular, spiculated, lobular & spiculated) |  |  |
| Nodule shape (round vs. polygonal) | Nodule shape (round vs. polygonal) | > 1 |
| Well-defined border (Yes) | Well-defined border (Yes) | > 1 |
| Lobulated margin (Yes) | Lobulated margin (Yes) | > 1 |
| Pleural tag (Yes) | Pleural tag (Yes) | 1 |
| Marginal hyper-attenuation (Yes) | Marginal hyper-attenuation (Yes) | 1 |
| Perifissure distribution (Yes) | Perifissure distribution (Yes) | 1 |
| Peripheral distribution (Yes) | Peripheral distribution (Yes) | > 1 |
| Involvement (Present vs. Absent) | Involvement (Present vs. Absent) | 1 |
| Vessel change (Yes) | Vessel change (Yes) | 1 |
| Pleural/fissure retraction (Yes) | Pleural/fissure retraction (Yes) | > 1 |
| Subpleural distribution (Yes) | Subpleural distribution (Yes) | 1 |
| STD of CT attenuation, HU | STD of CT attenuation, HU | > 1 |
| Total number of CT scans | Total number of CT scans | 1 |
| Mean of CT attenuation, HU | Mean of CT attenuation, HU | # |
| Mean CT attenuation (> -500HU) | Mean CT attenuation (> -500HU) | 1 |
| Mean CT attenuation (>=-677HU) | Mean CT attenuation(>=-677HU) | 1 |
| Max CT attenuation, HU | Max CT attenuation, HU | > 1 |
| Min CT attenuation, HU | Min CT attenuation, HU | 1 |
| Volume, mm3 | Volume, mm3 | > 1 |
| Mass, mg | Mass, mg | > 1 |
| Density, mg/mm3 | Density, mg/mm3 | 1 |
| 2.5th percentile, HU | 2.5th percentile, HU | 1 |
| 10th percentile, HU | 10th percentile, HU | 1 |
| 25th percentile, HU | 25th percentile, HU | 1 |
| 50th percentile, HU | 50th percentile, HU | 1 |
| 75th percentile, HU | 75th percentile, HU | 1 |
| 90th percentile, HU | 90th percentile, HU | 1 |
| 97th percentile, HU | 97th percentile, HU | 1 |
| Slope,2.5th to 97.5th percentiles | Slope,2.5th to 97.5th percentiles | 1 |
| Surface area, mm2 | Surface area, mm2 | 1 |
| Sphericity | Sphericity | 1 |
| Compactness | Compactness | 1 |
| Elongation | Elongation | 1 |
| Flatness | Flatness | 1 |
| Median CT attenuation, HU | Median CT attenuation, HU | 1 |
| Skewness | Skewness | 1 |
| Entropy | Entropy | 1 |
| Kurtosis | Kurtosis | 1 |
| Note: * Removed from meta-analysis because it is one of the criteria for SSN growth; # 11 features were investigated in one single study for multivariate analysis and more than one study for univariate study | | |


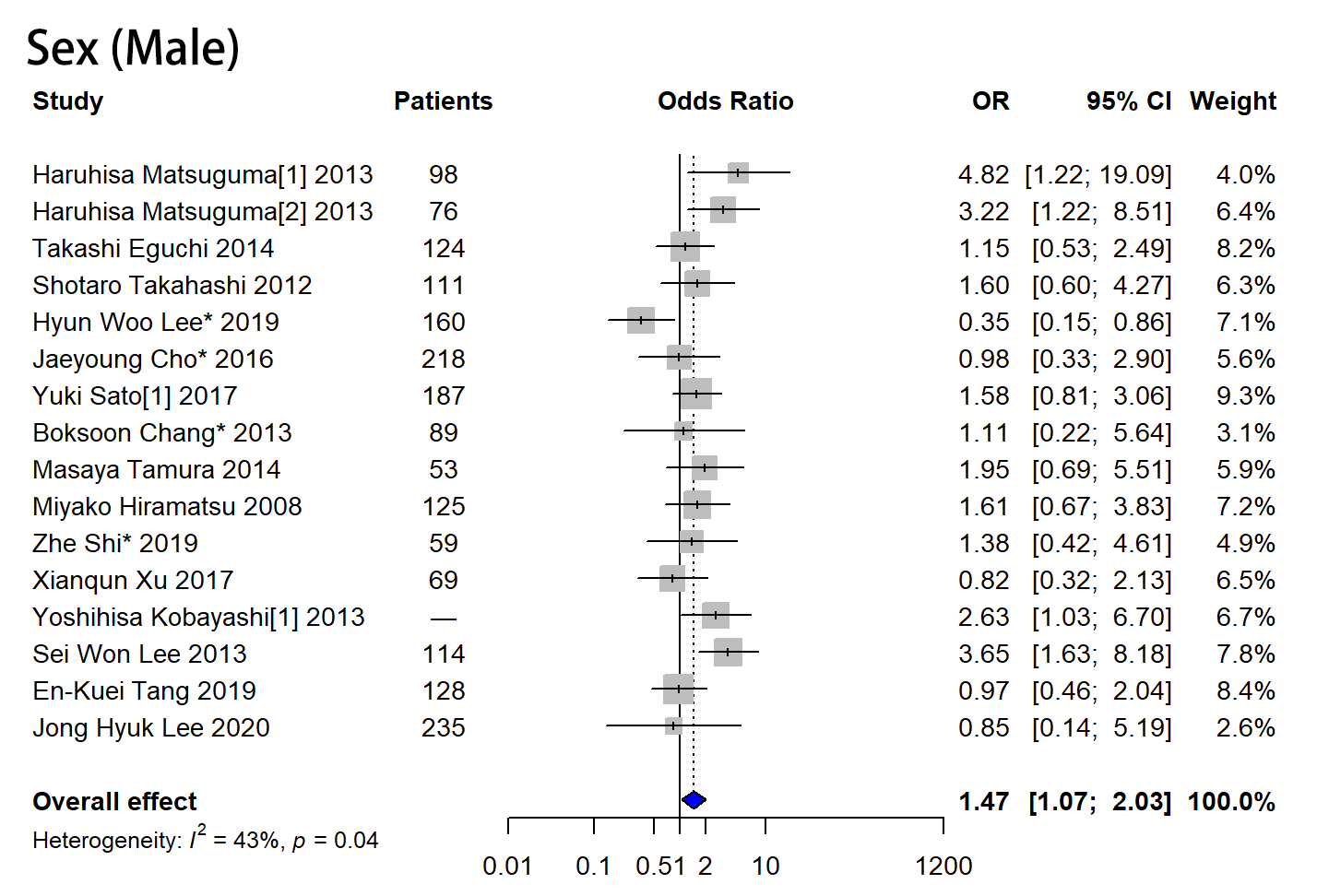

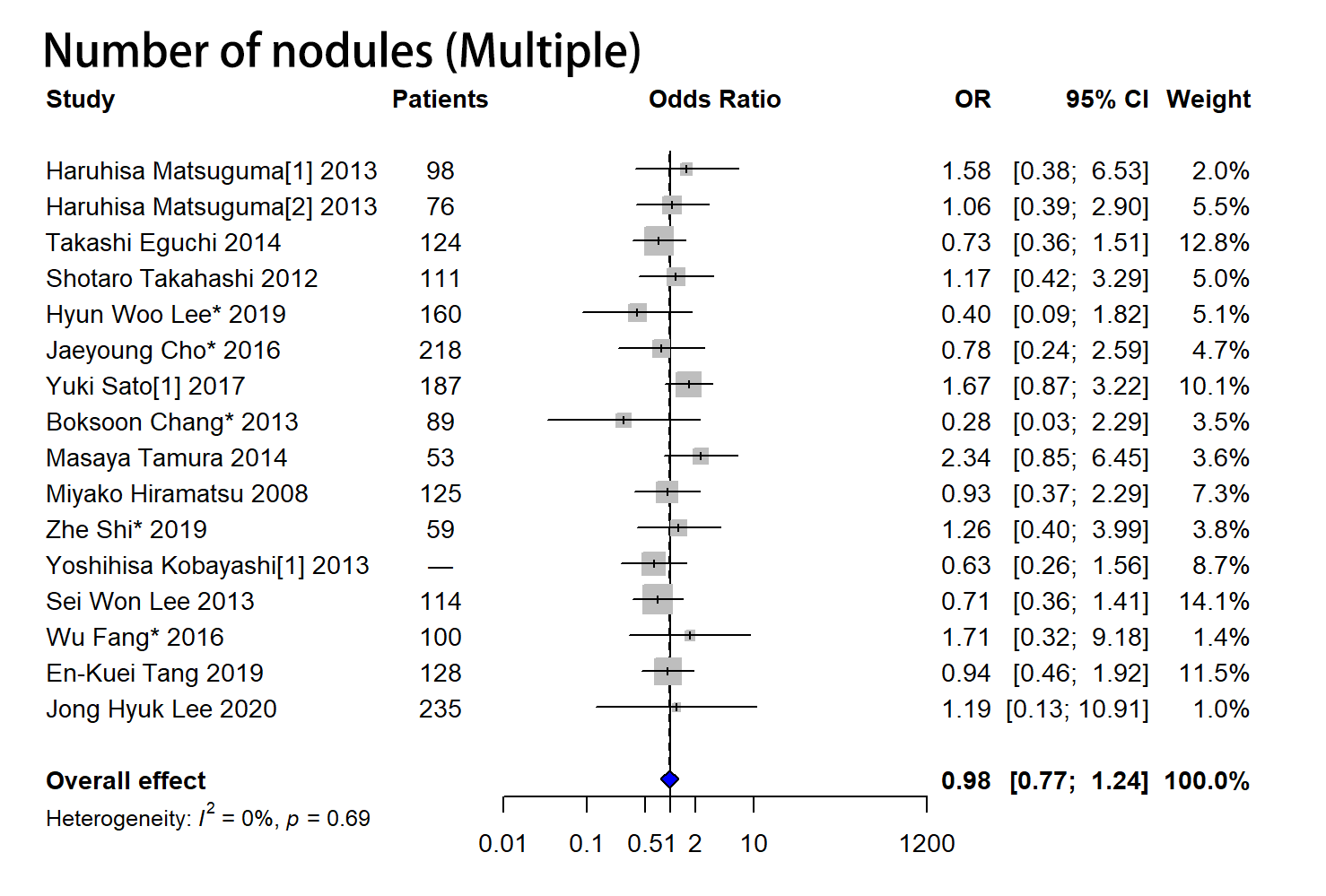

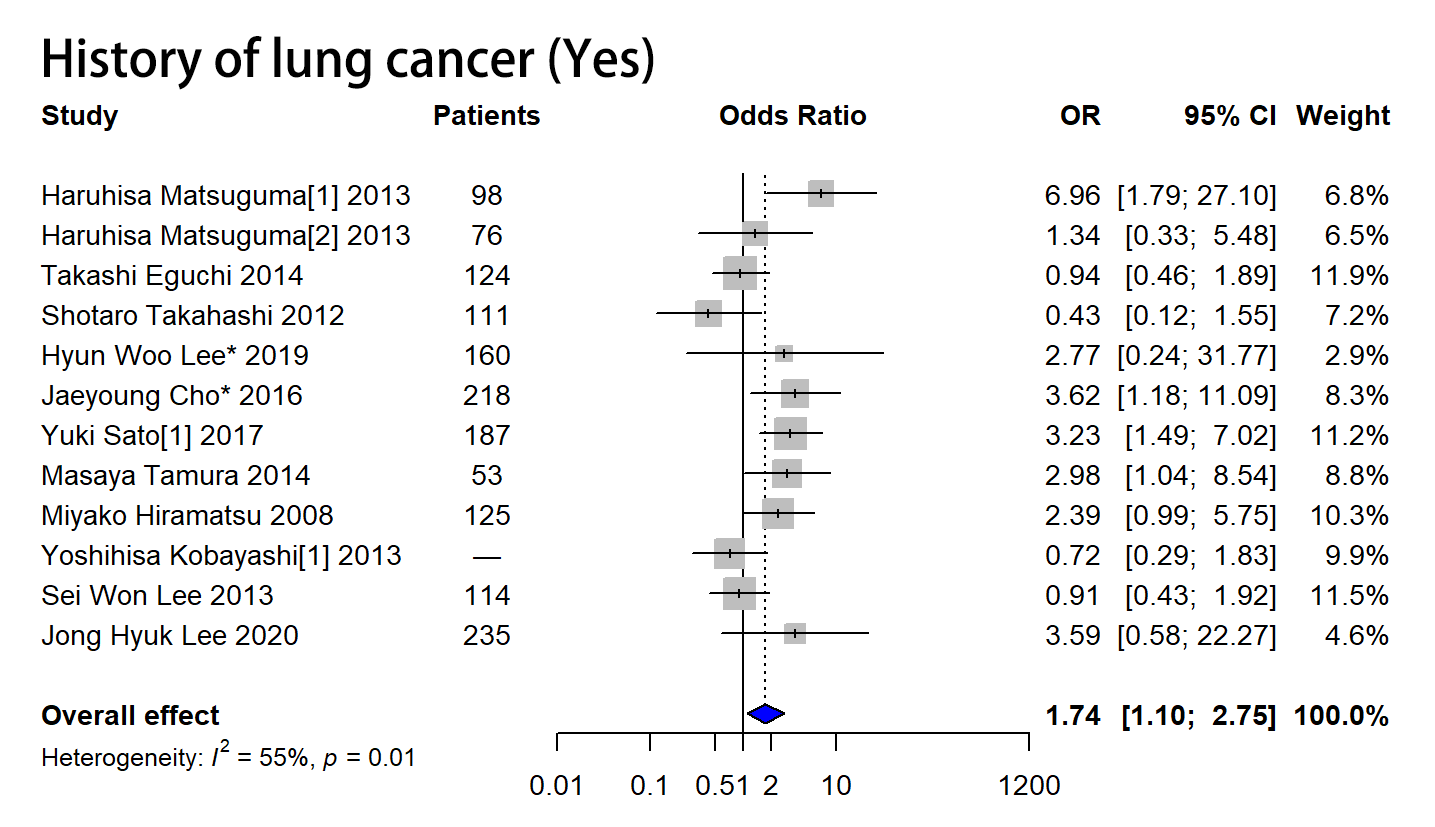

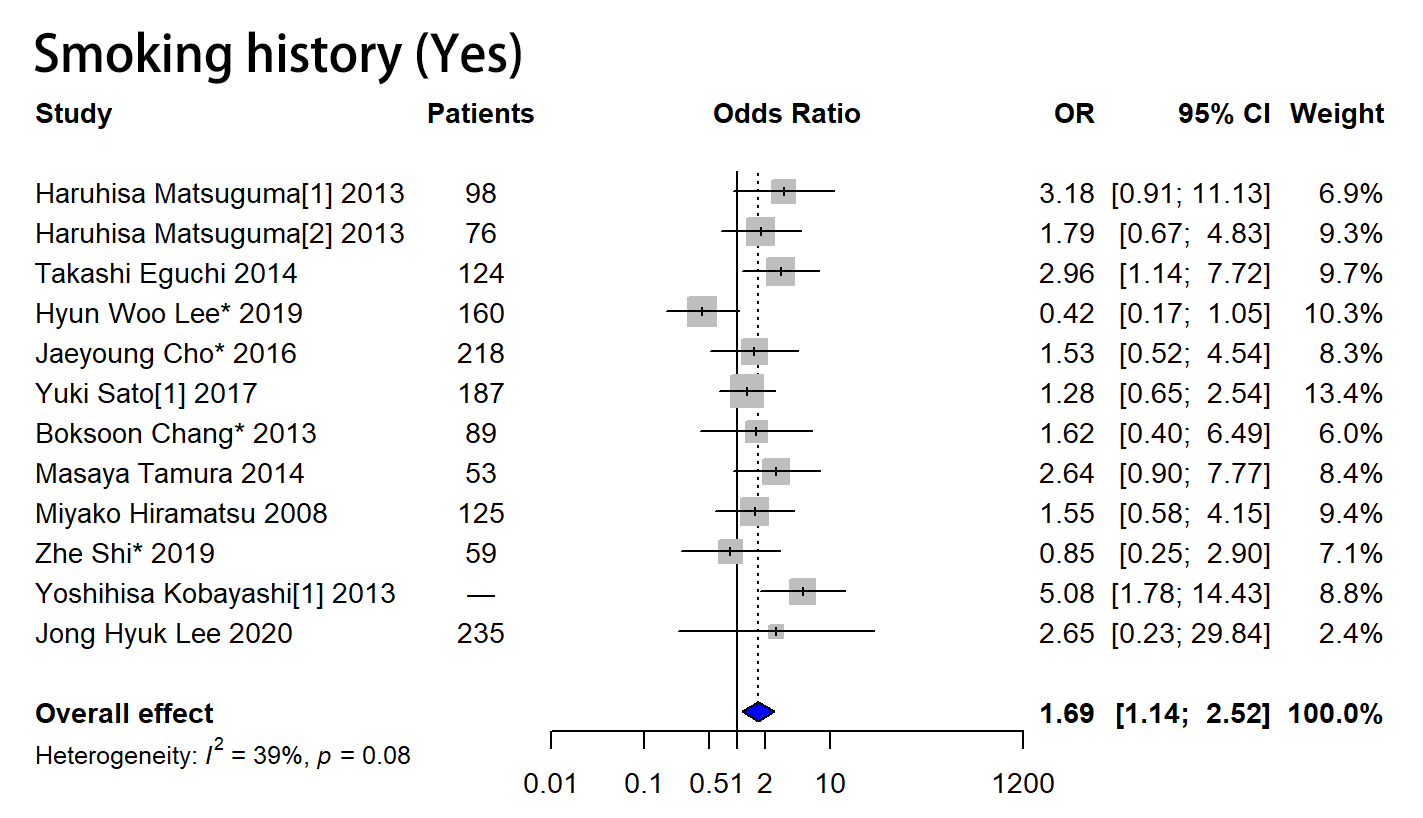

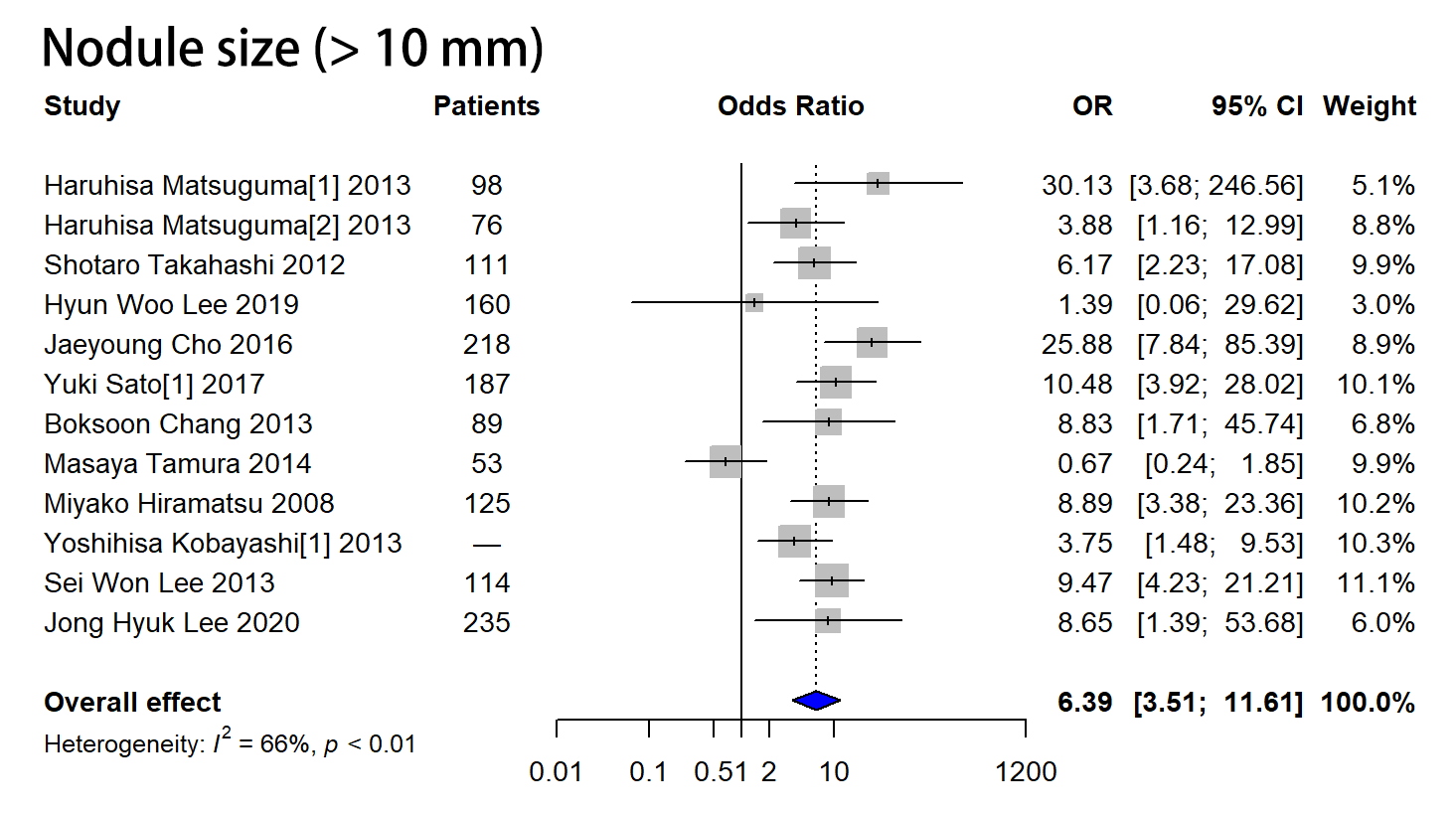

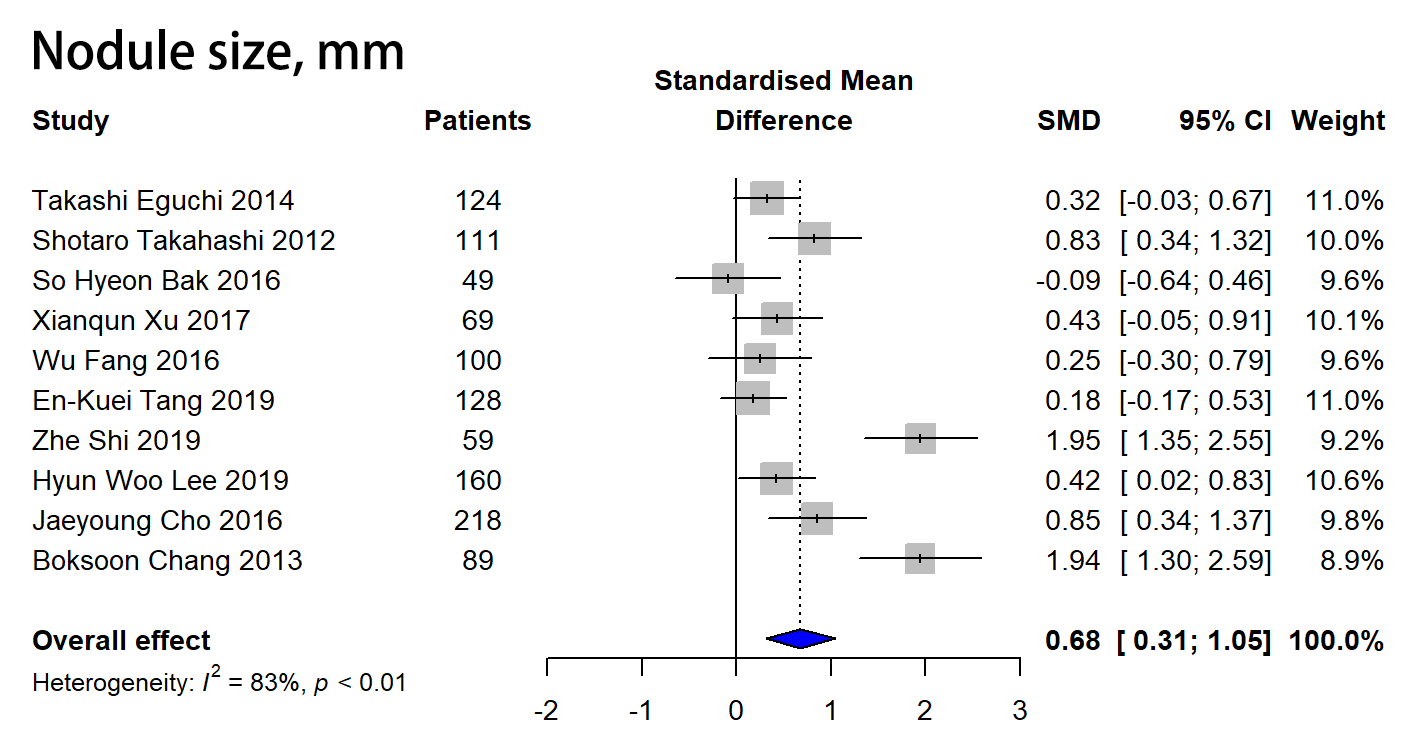

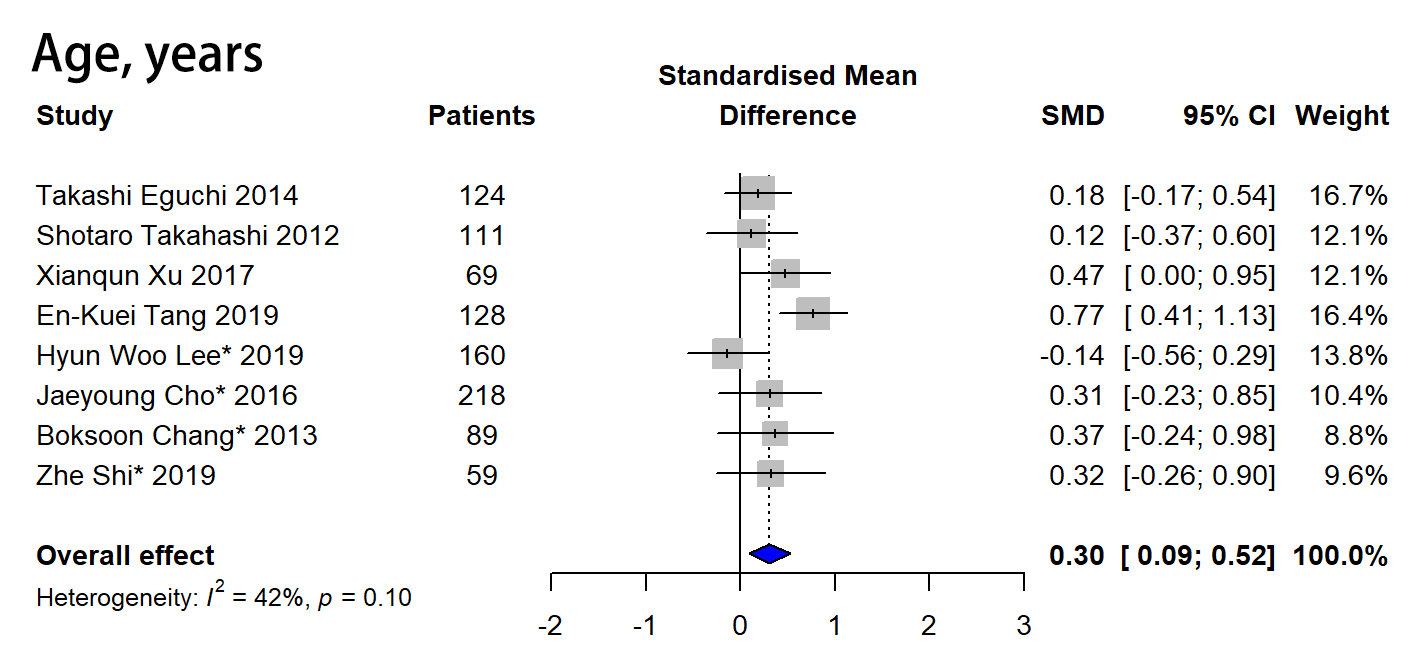

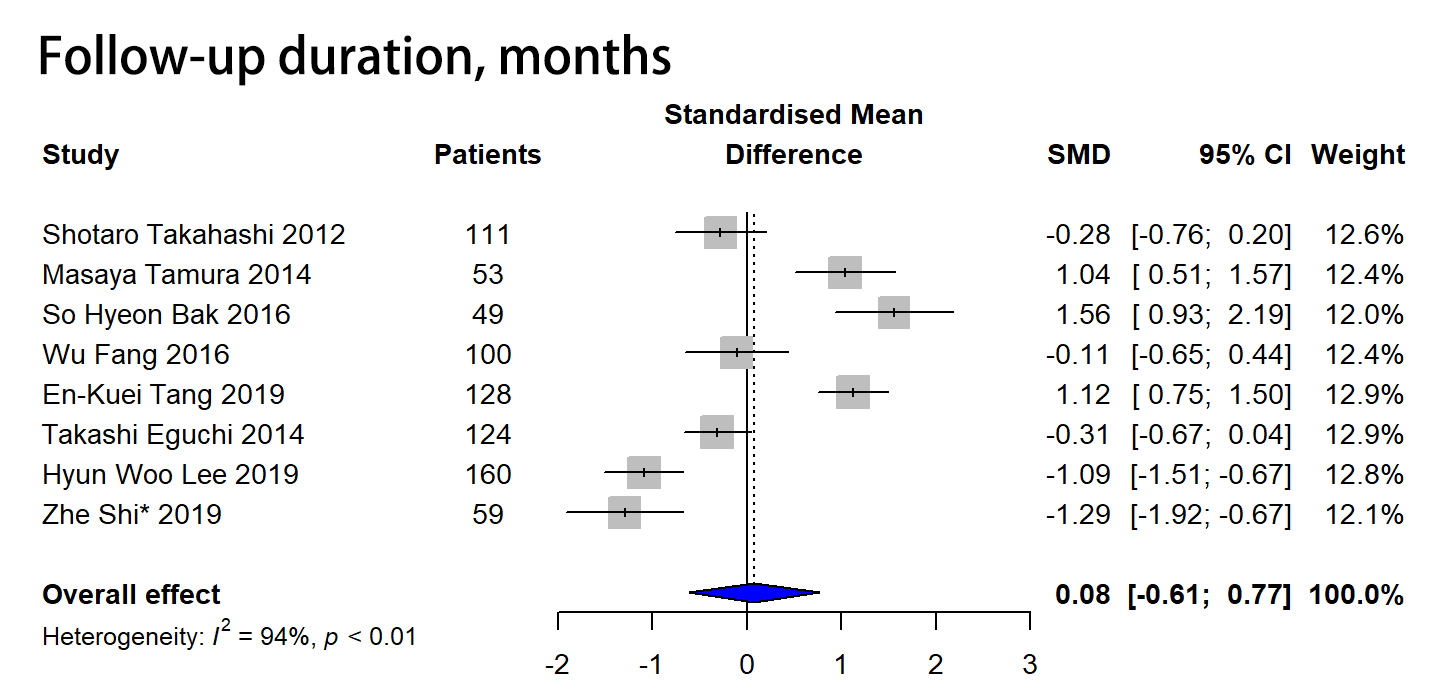

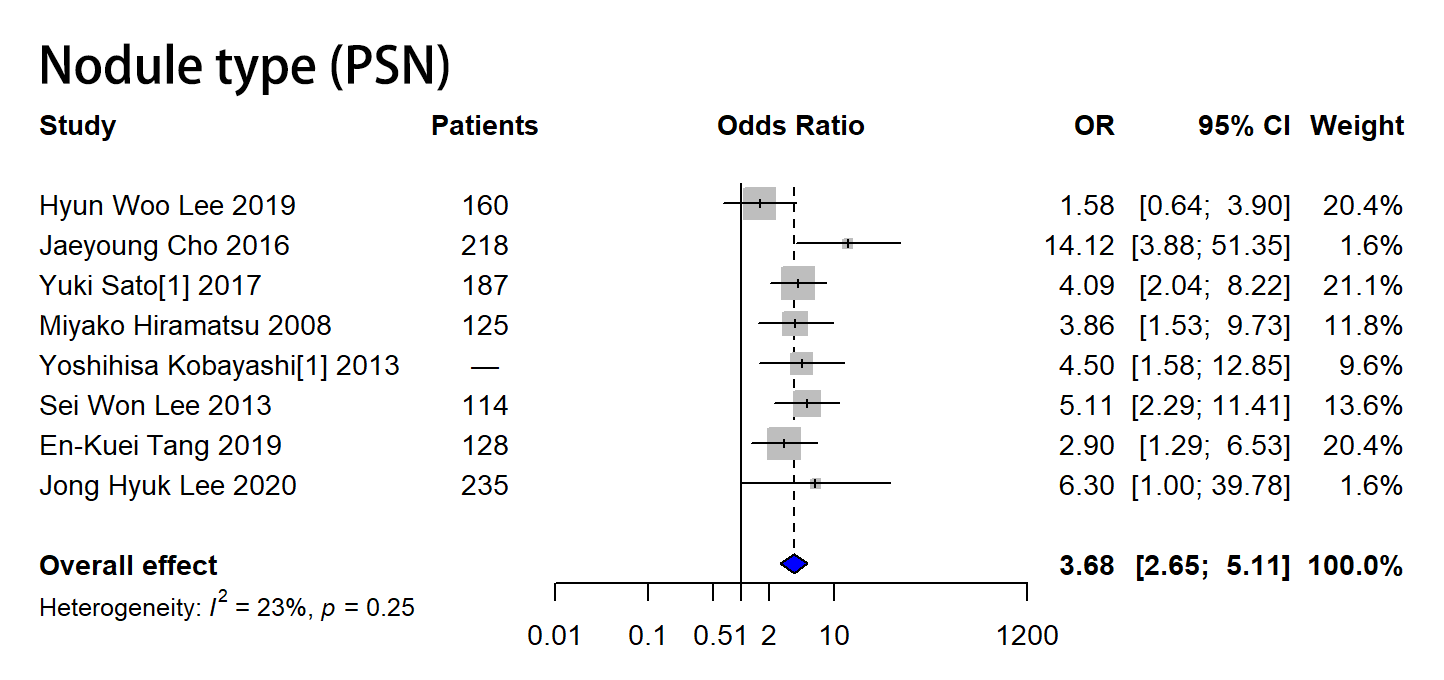

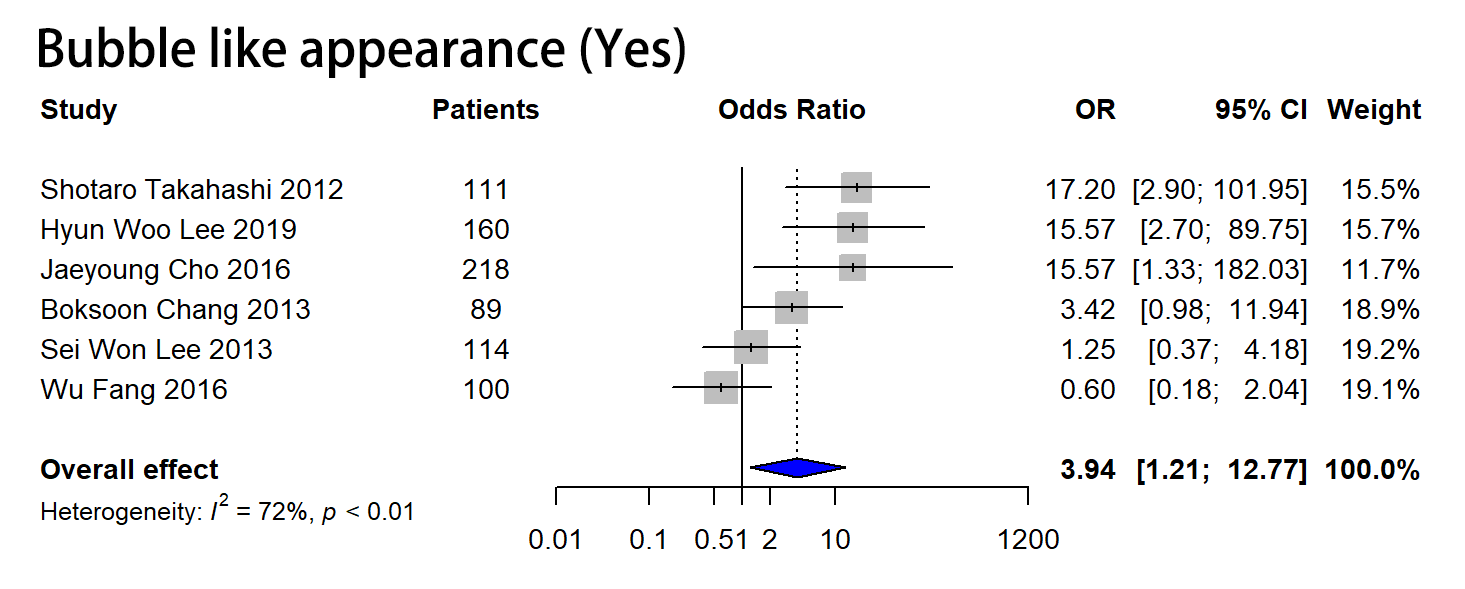

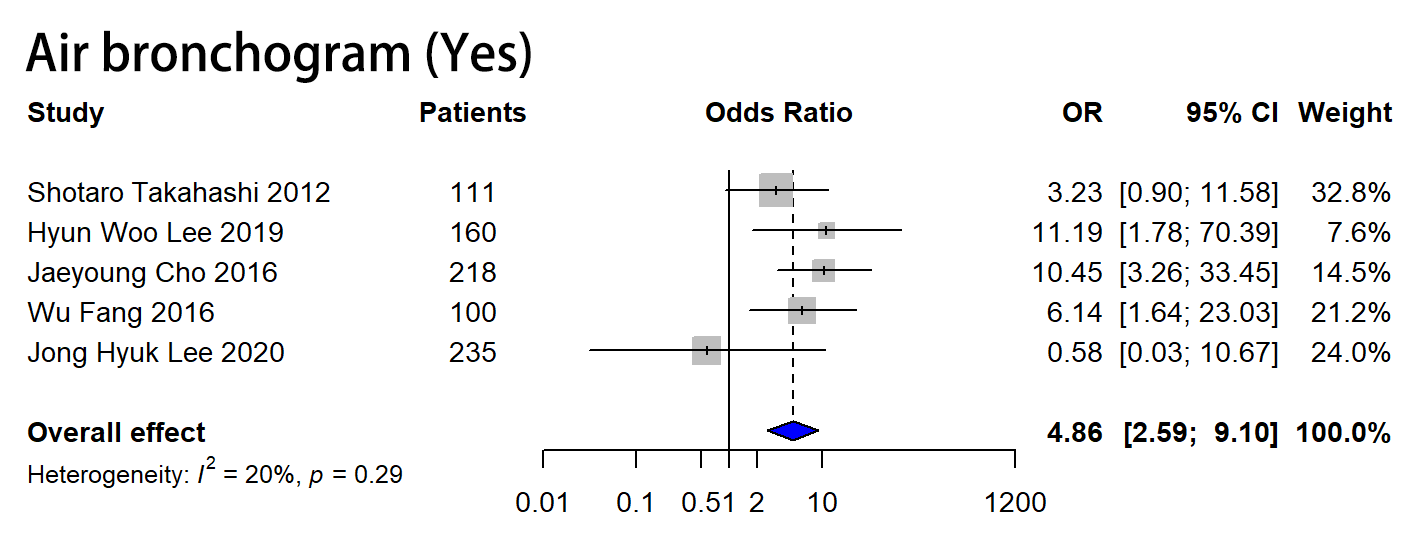

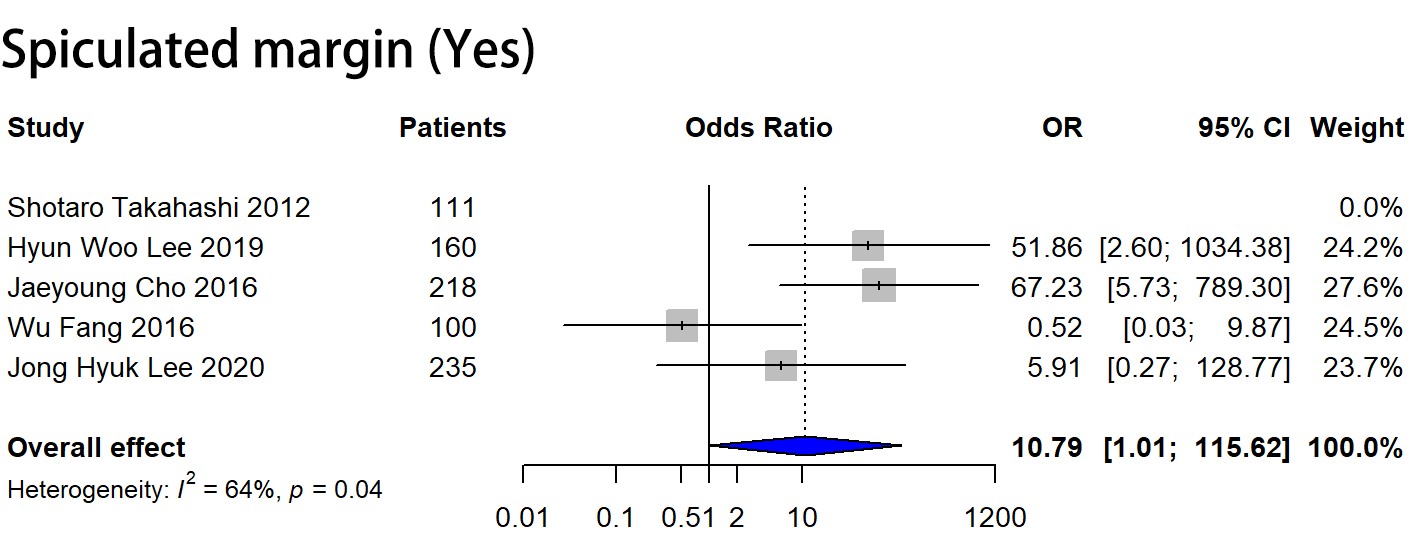

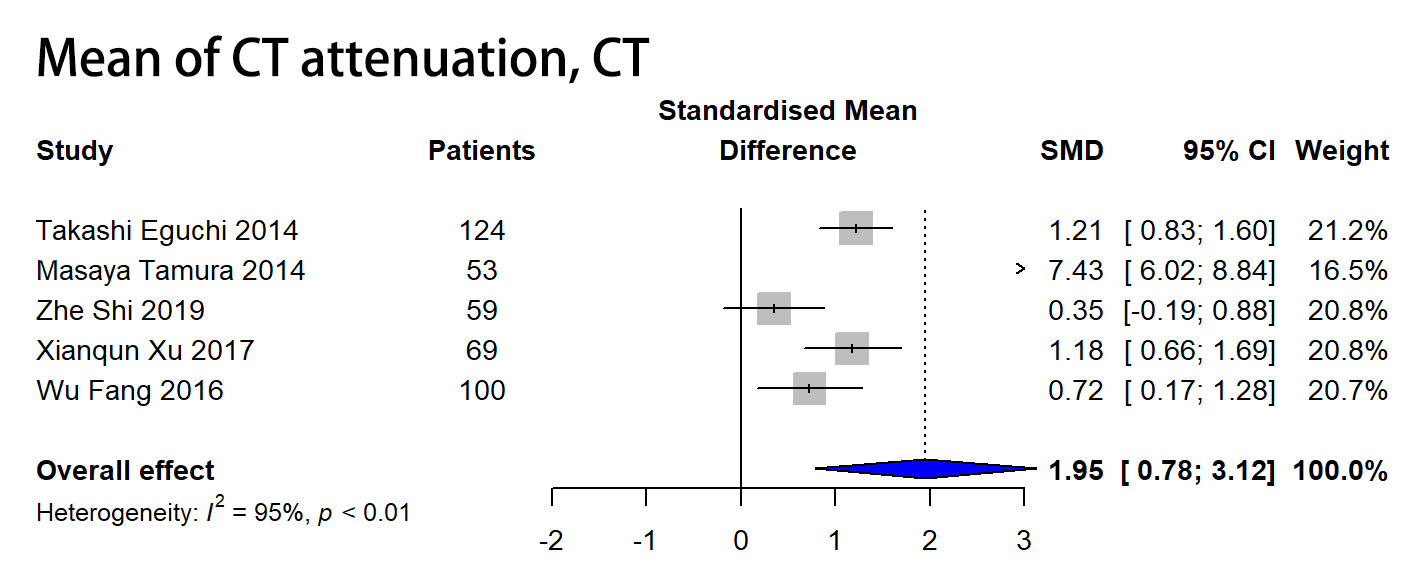

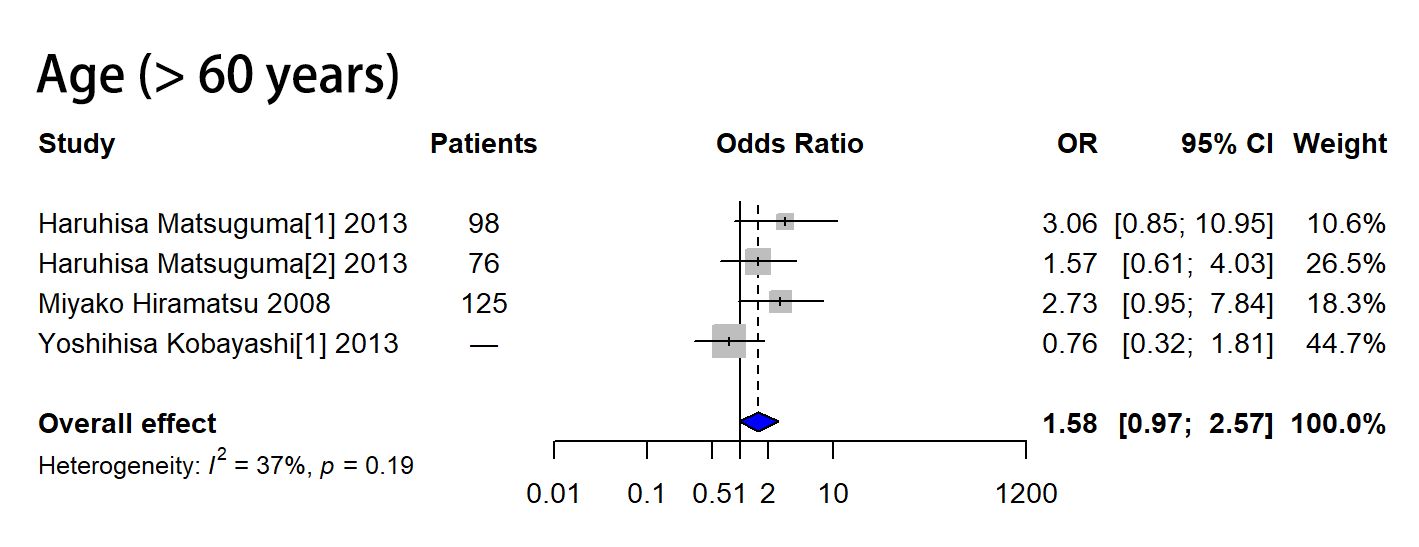

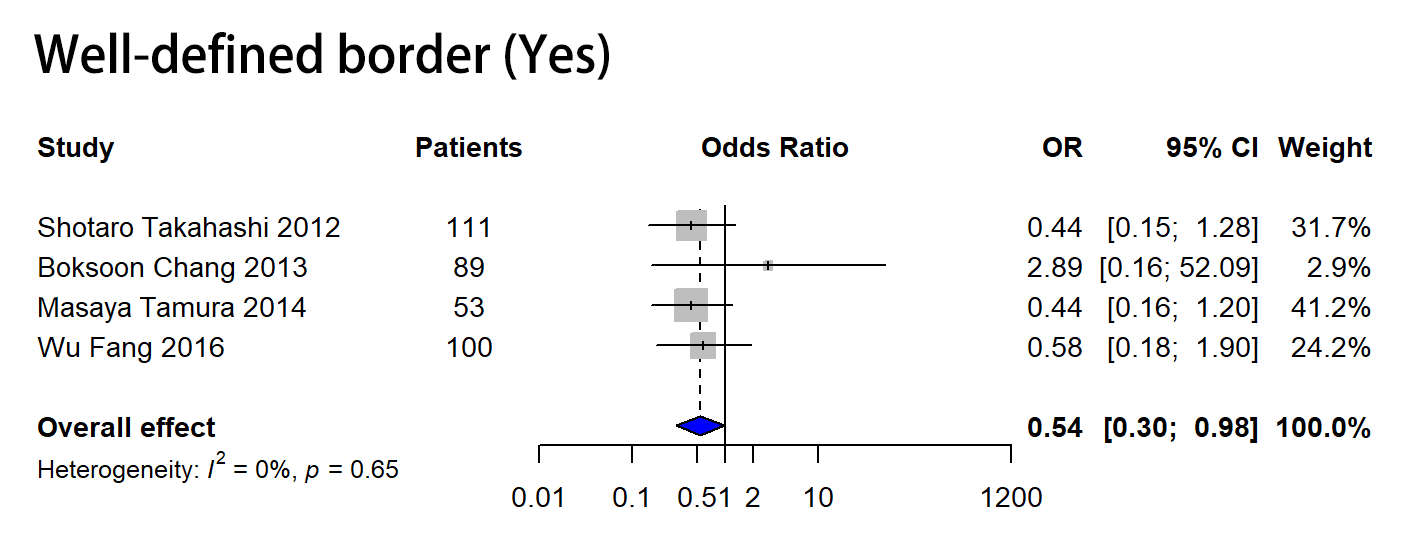

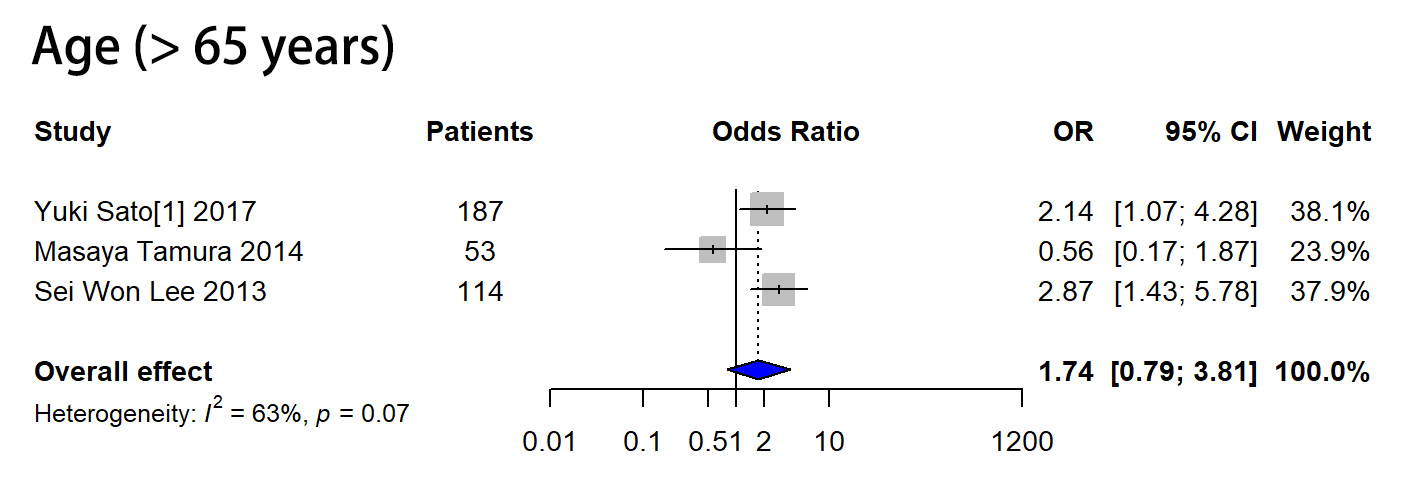

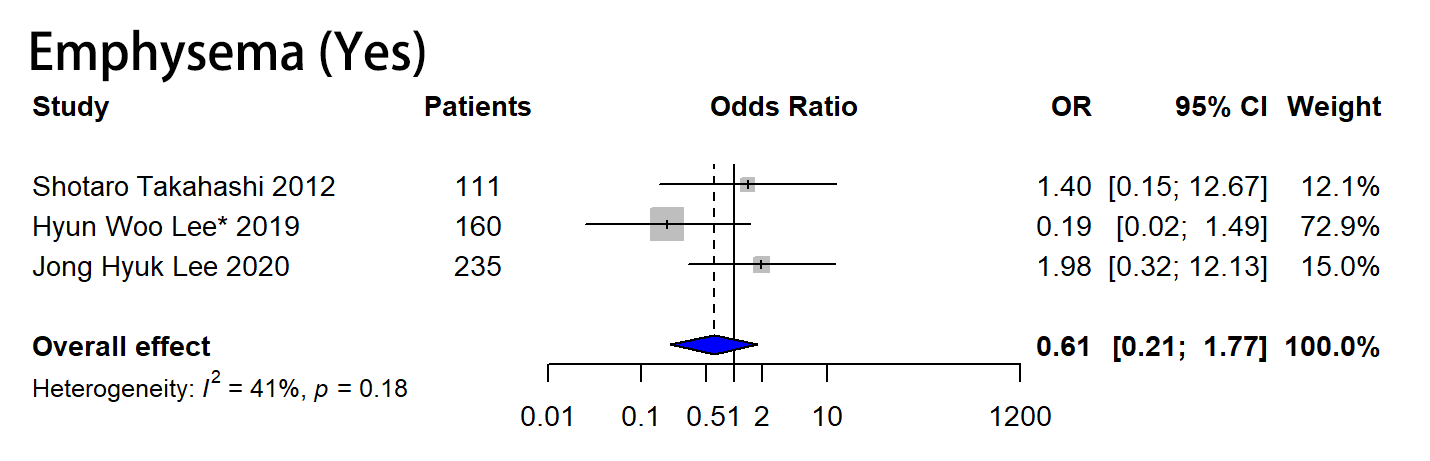

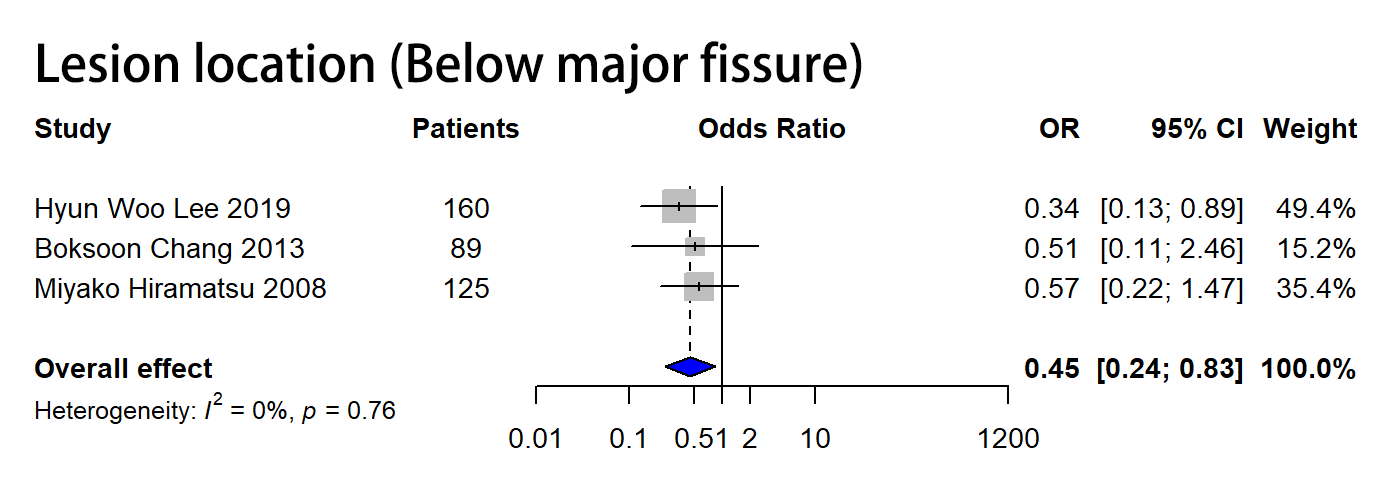

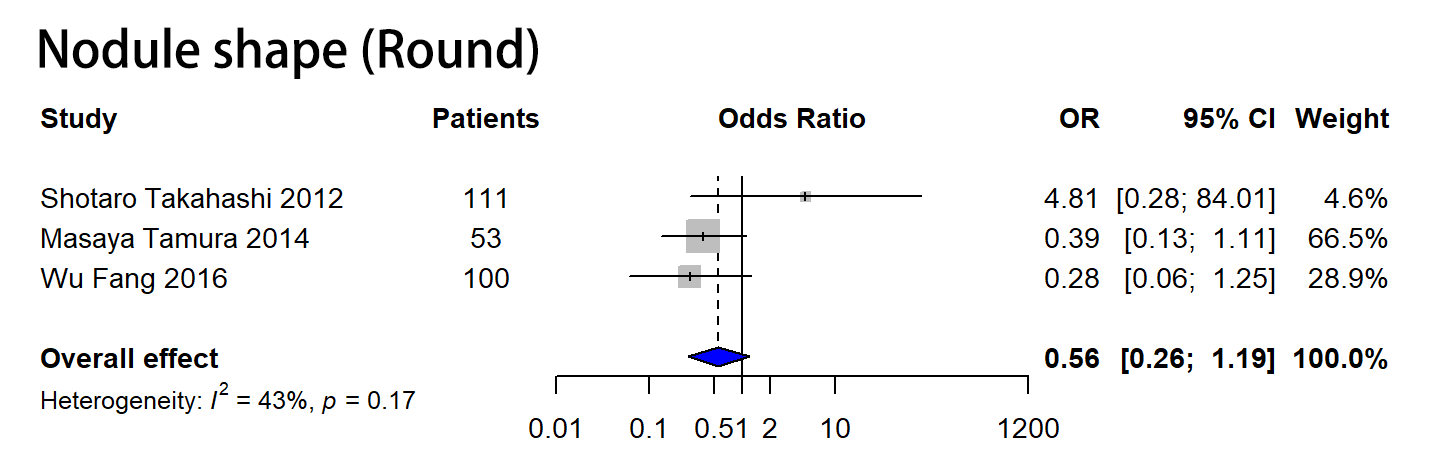

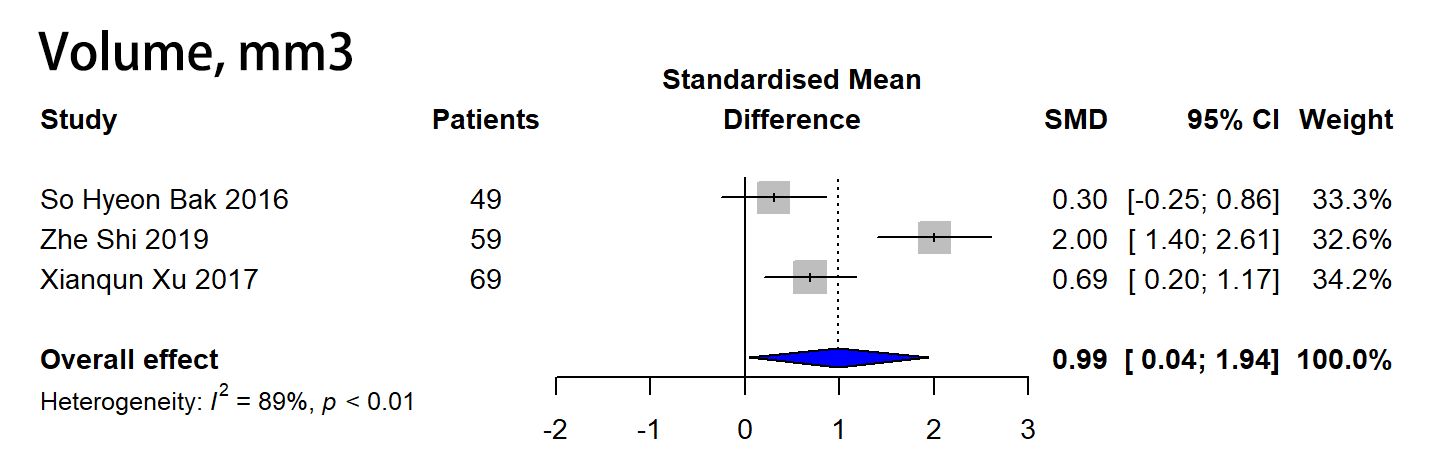

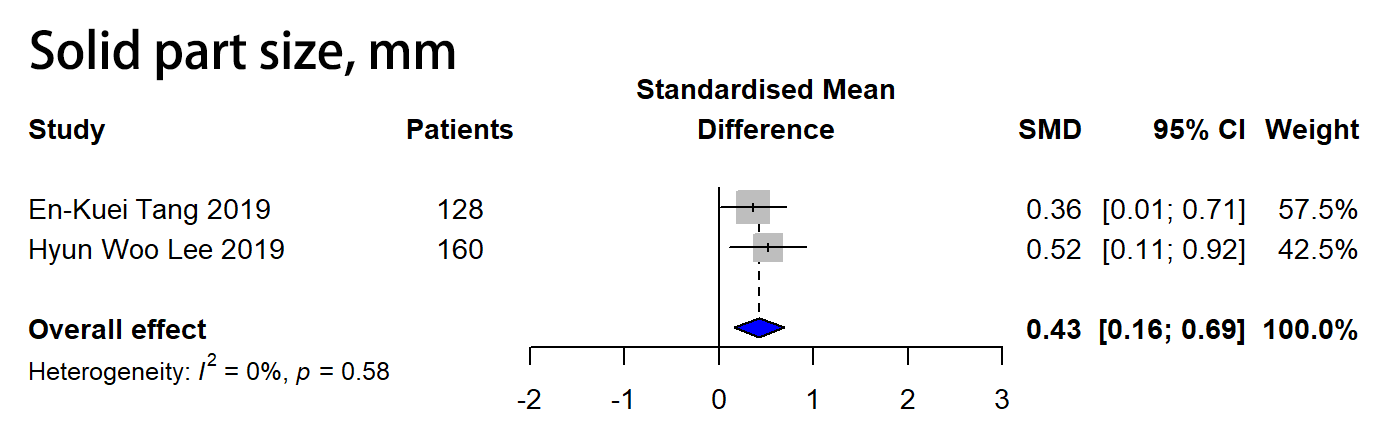

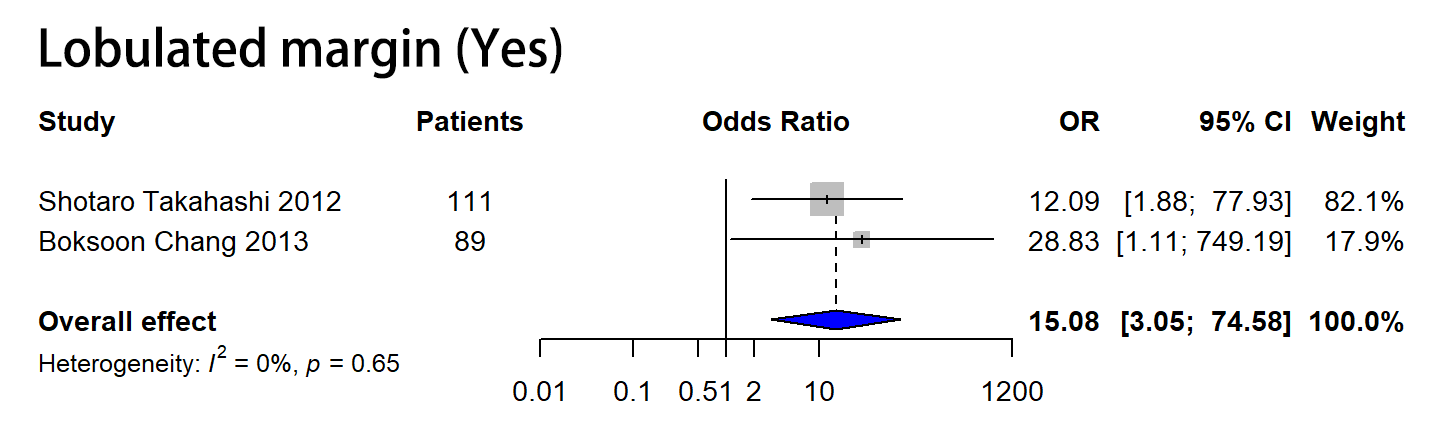

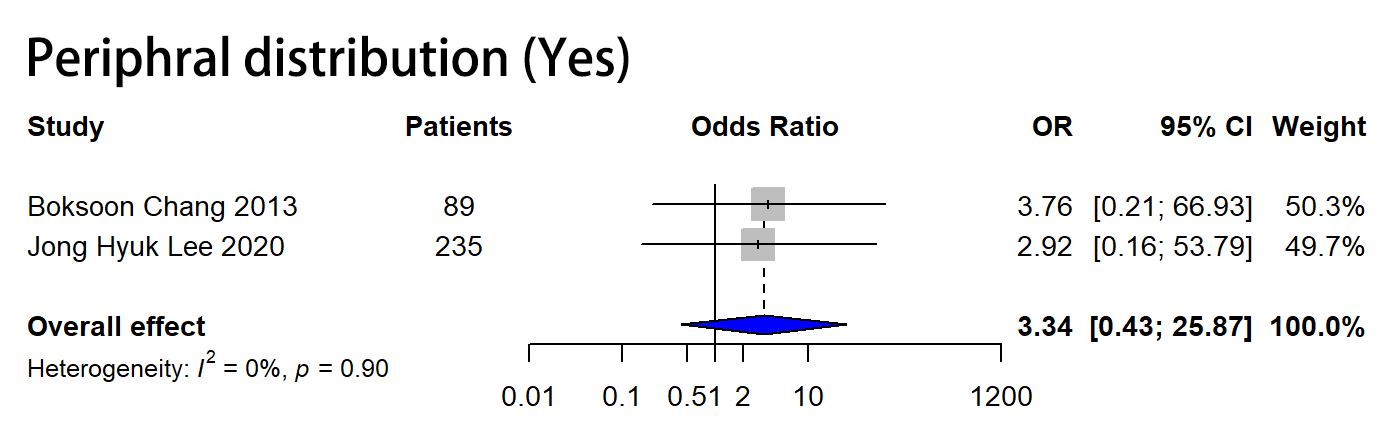

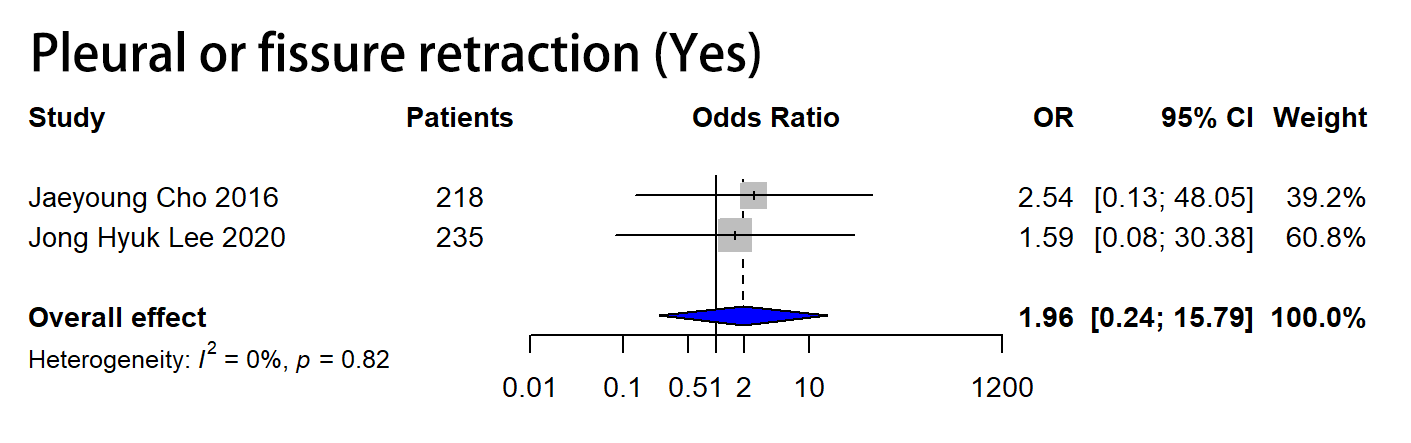

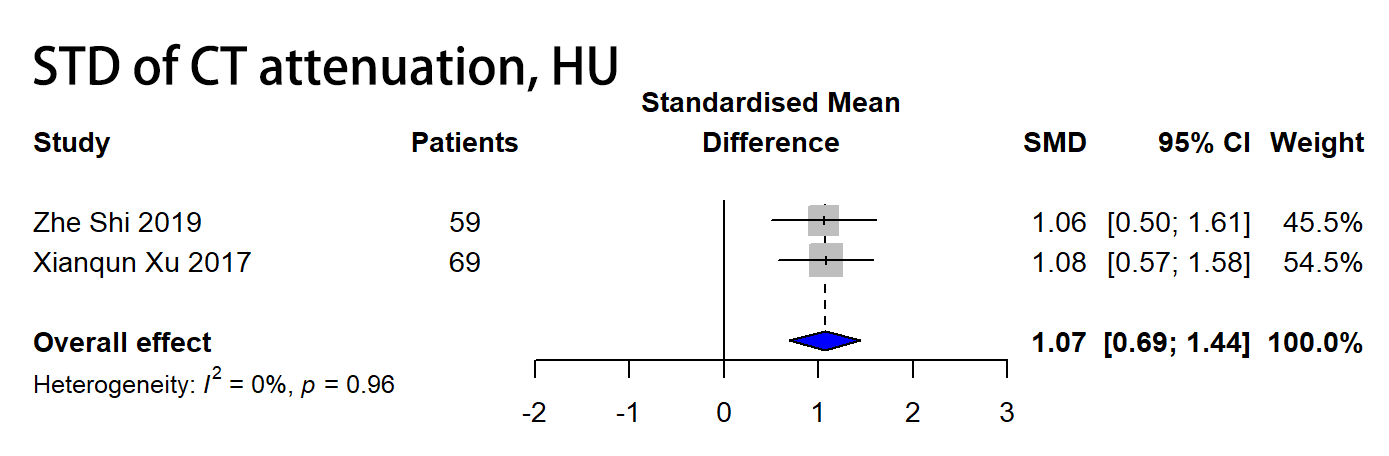

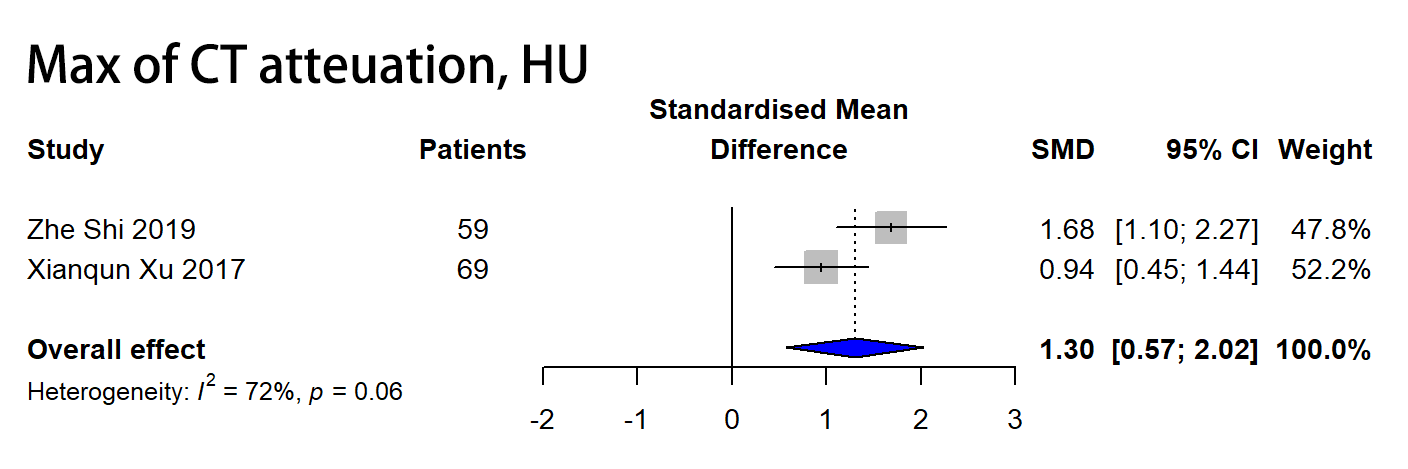

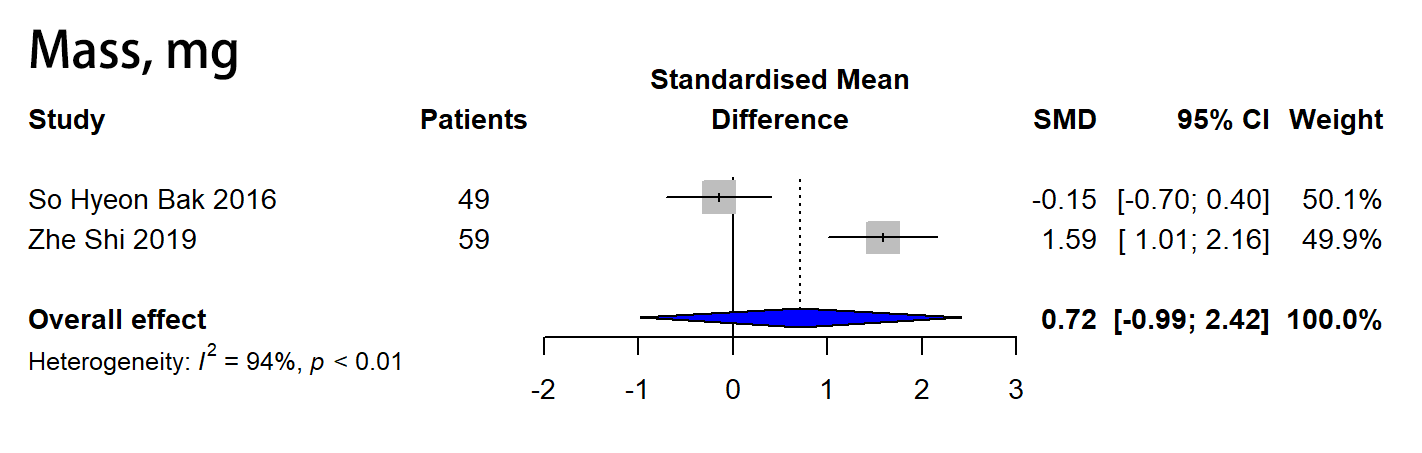


**Figure S1.** Forest plots of 27 features analyzed based on univariate analysis results in SSN.


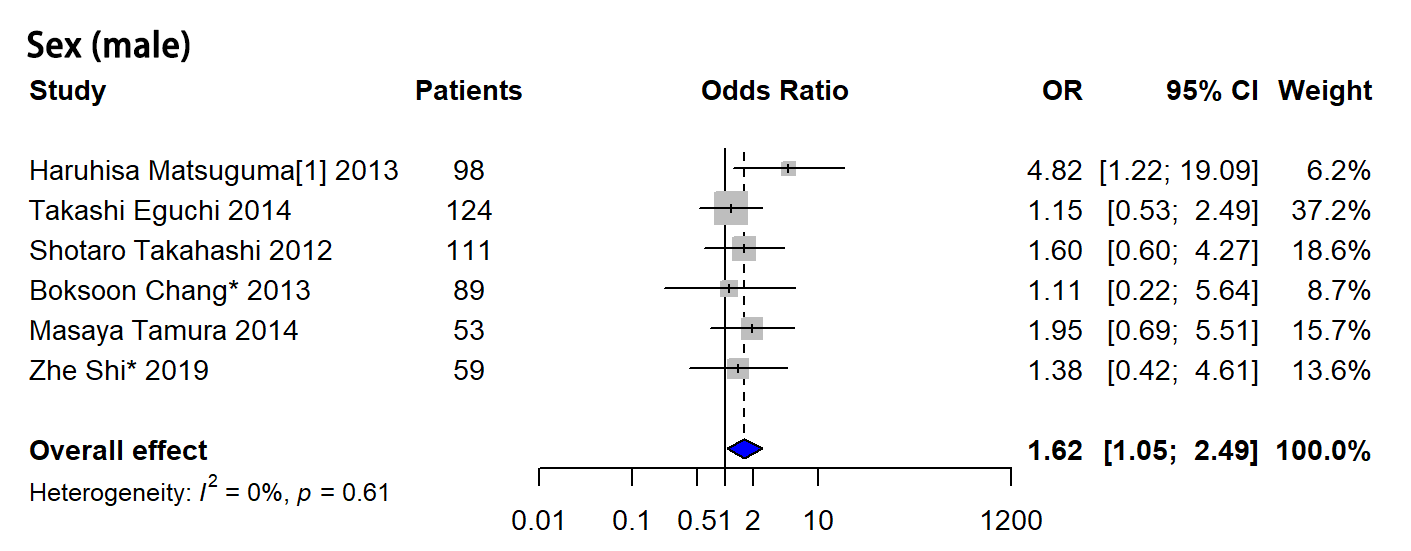

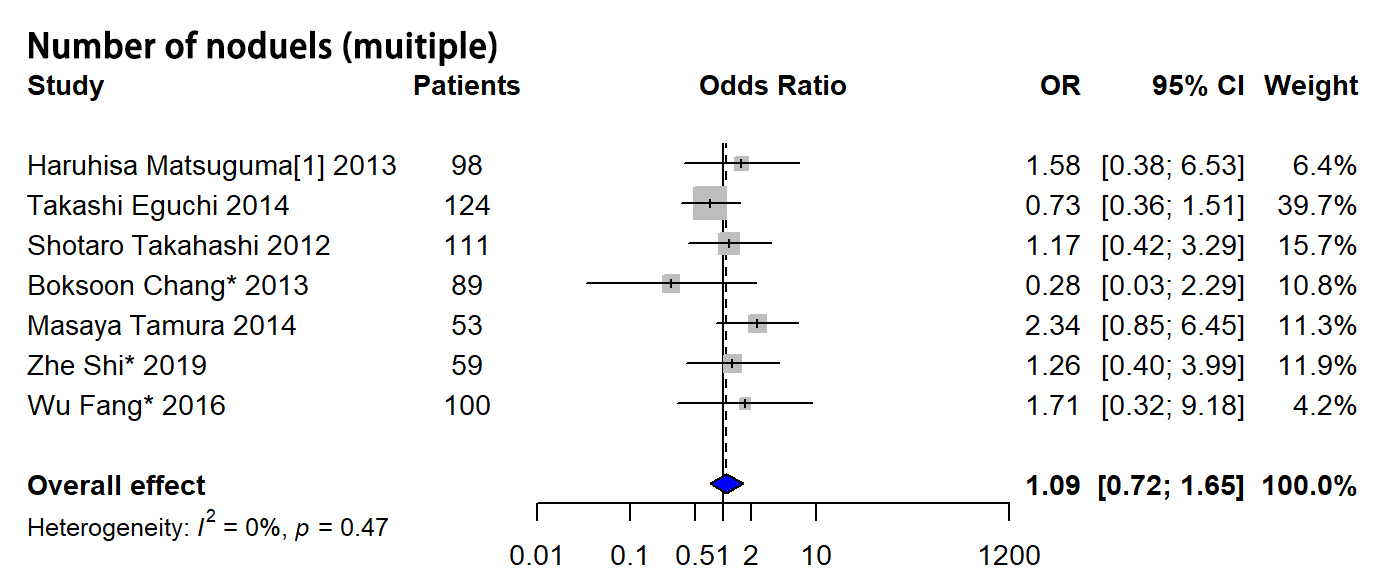

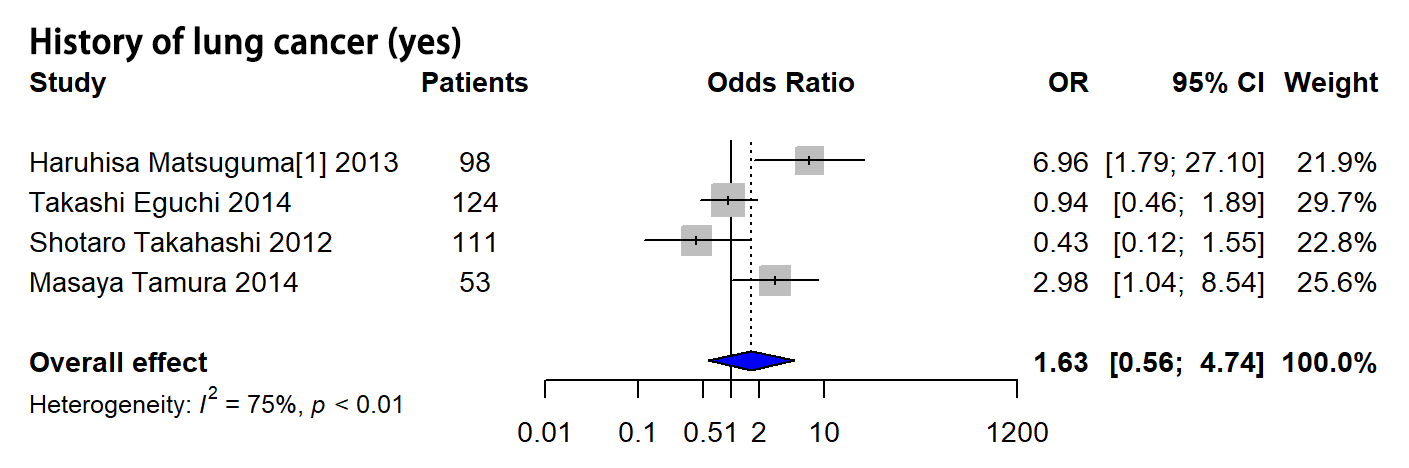

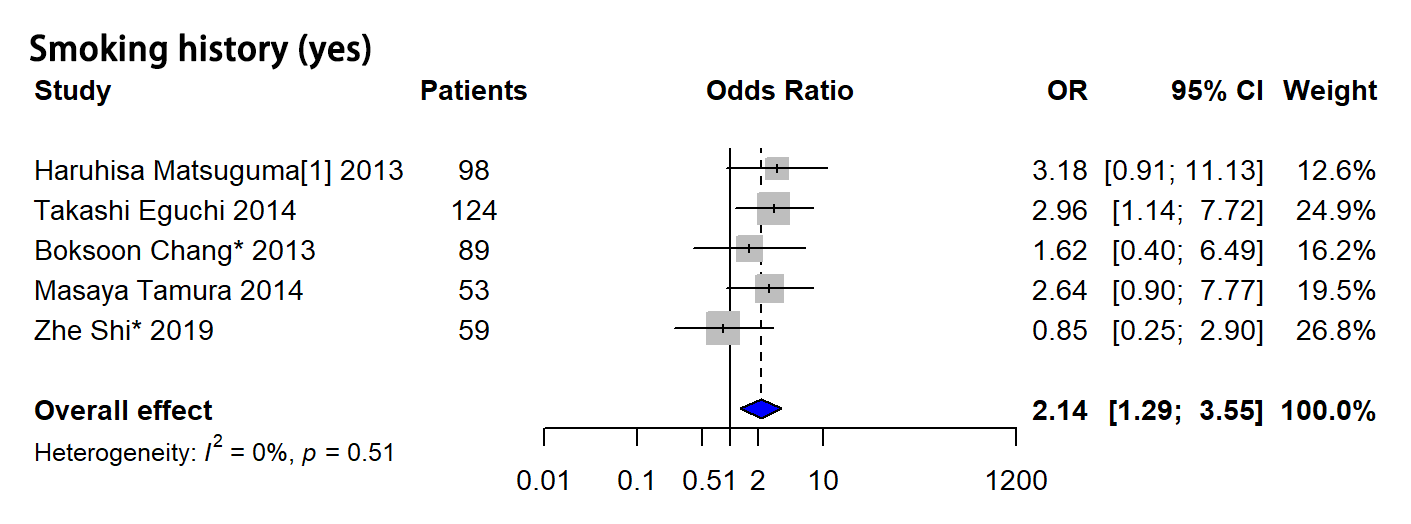

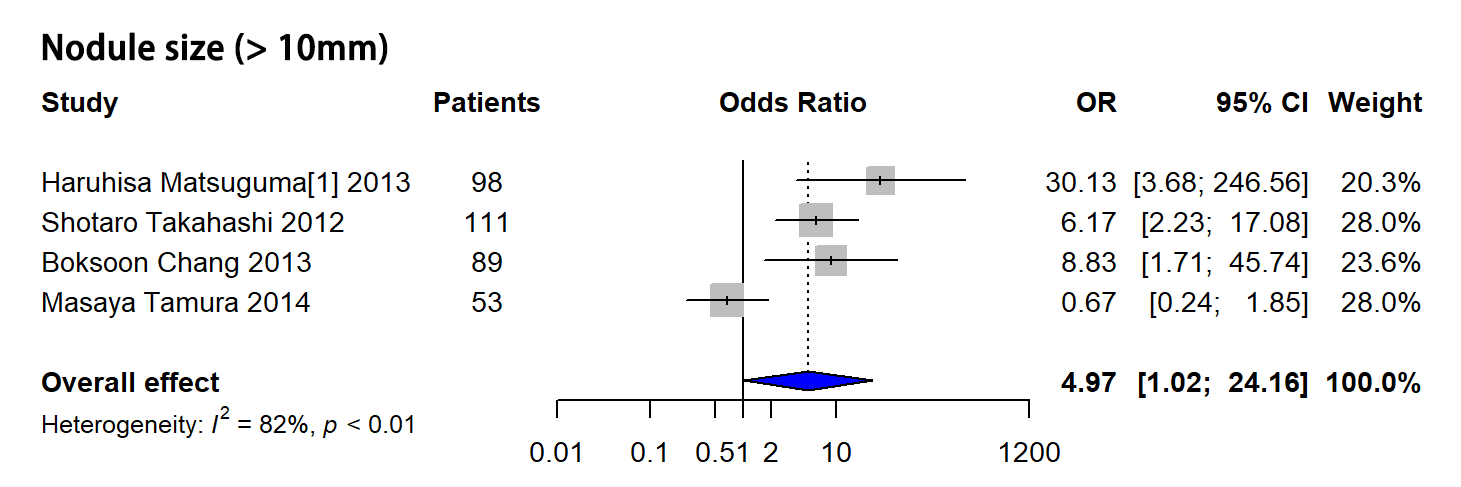

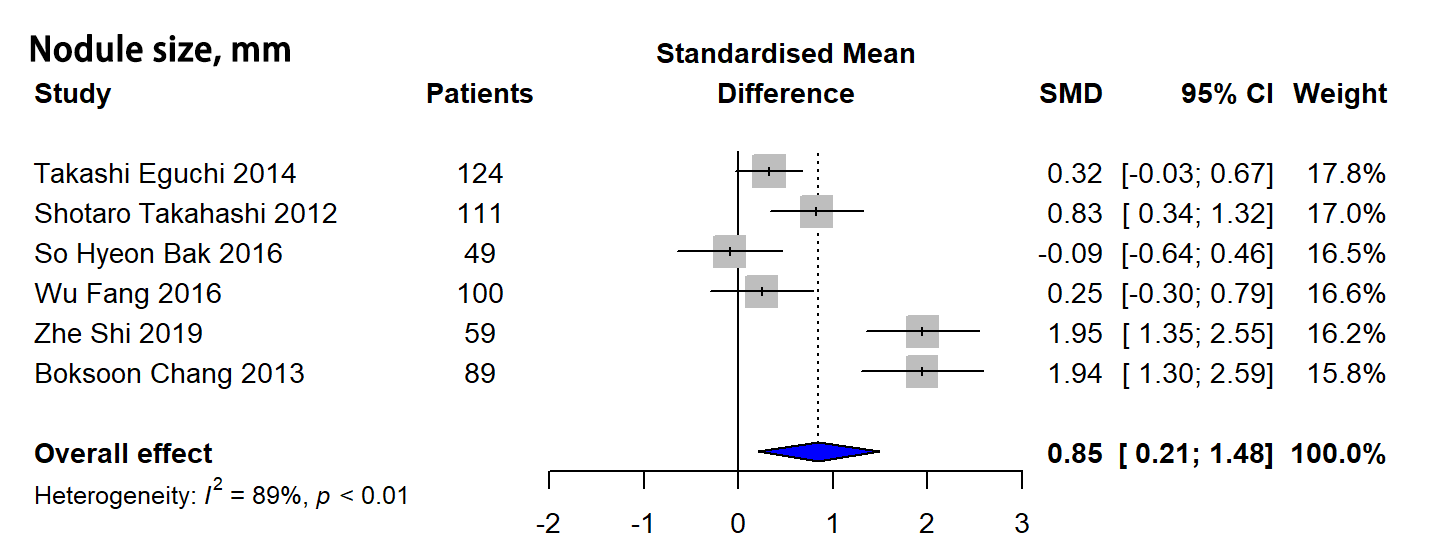

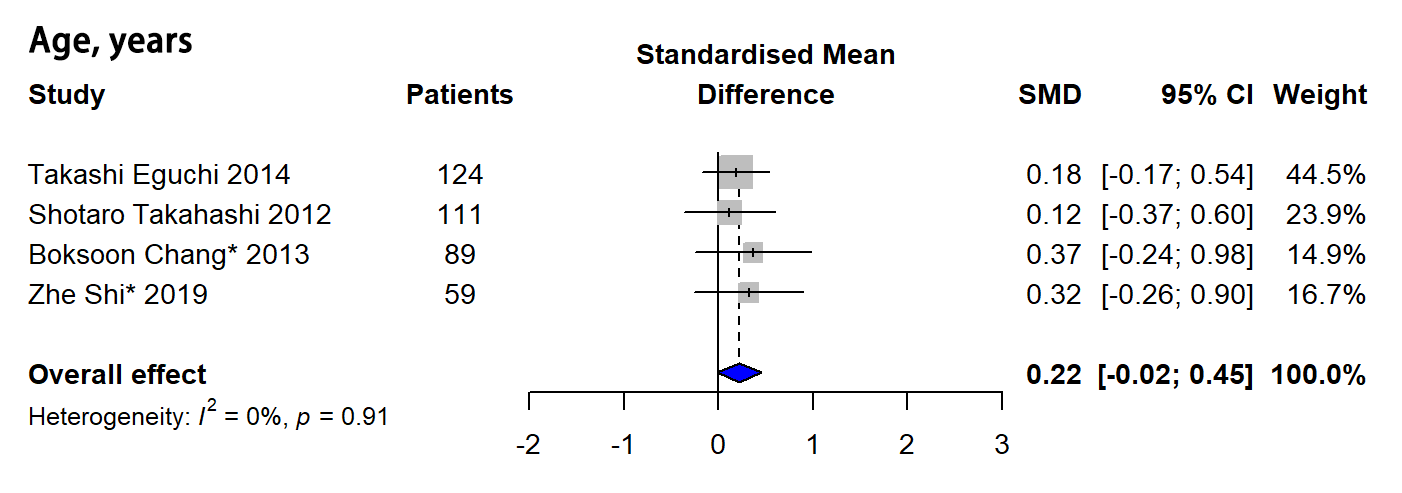

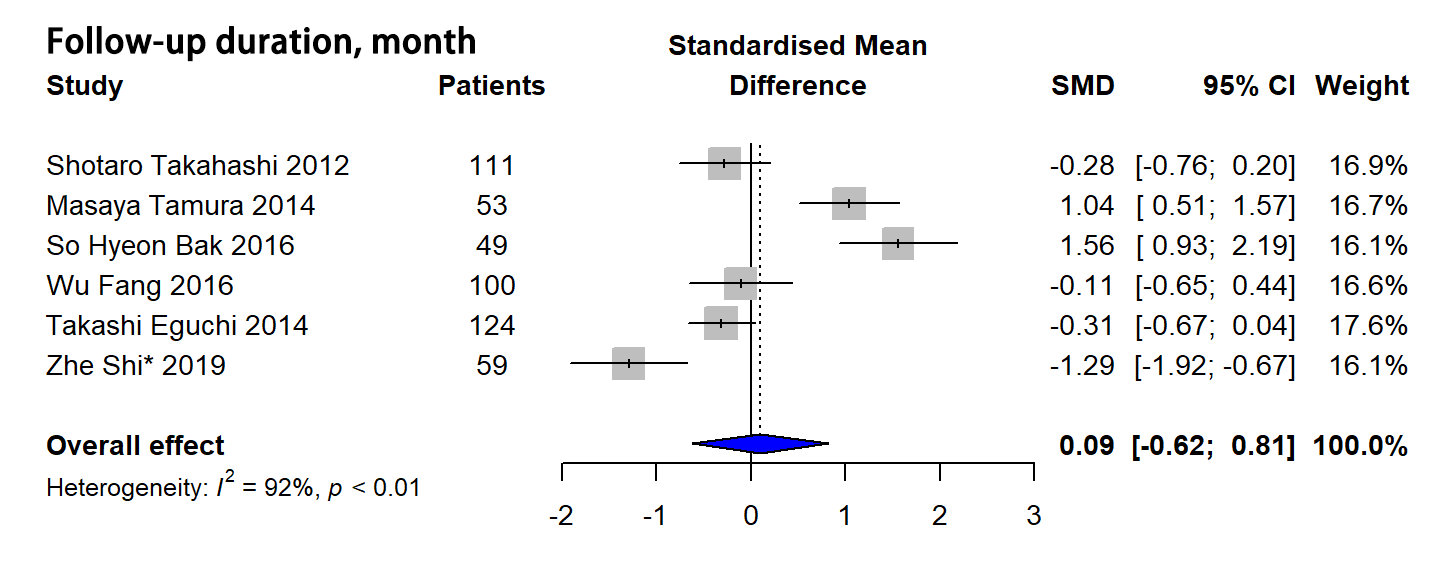

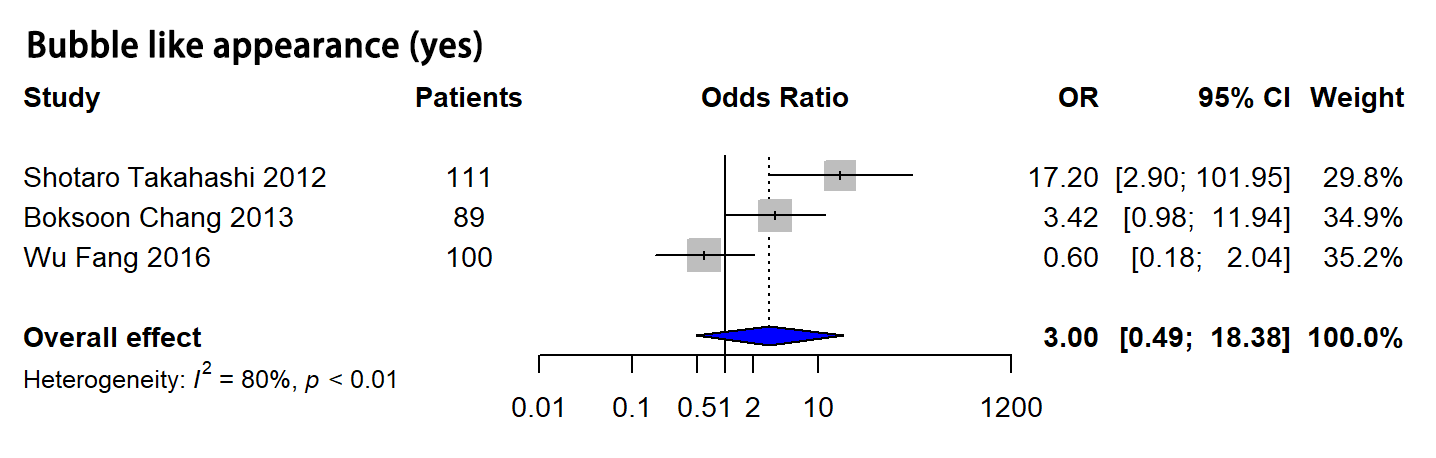

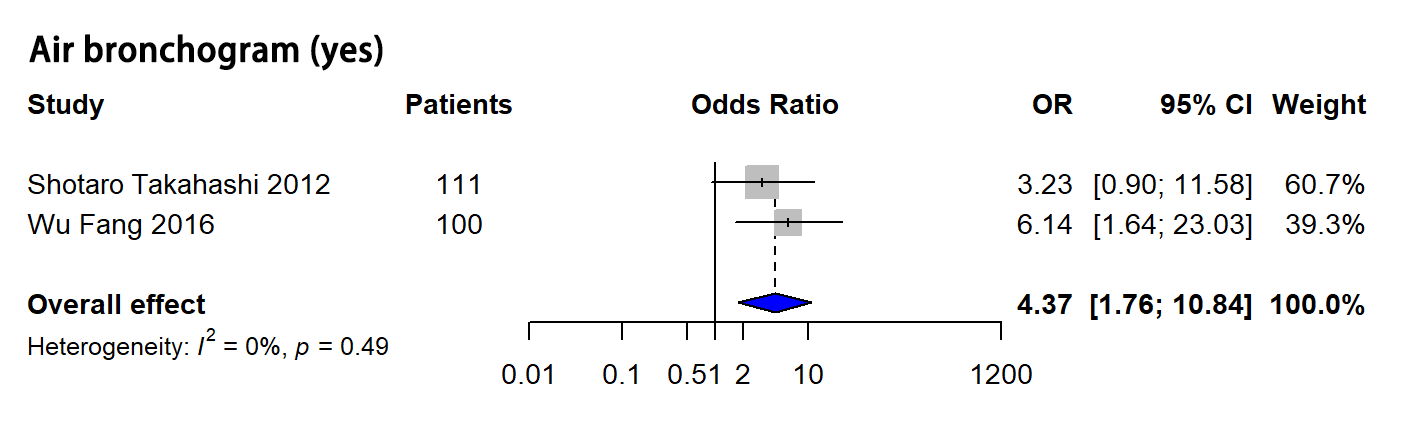

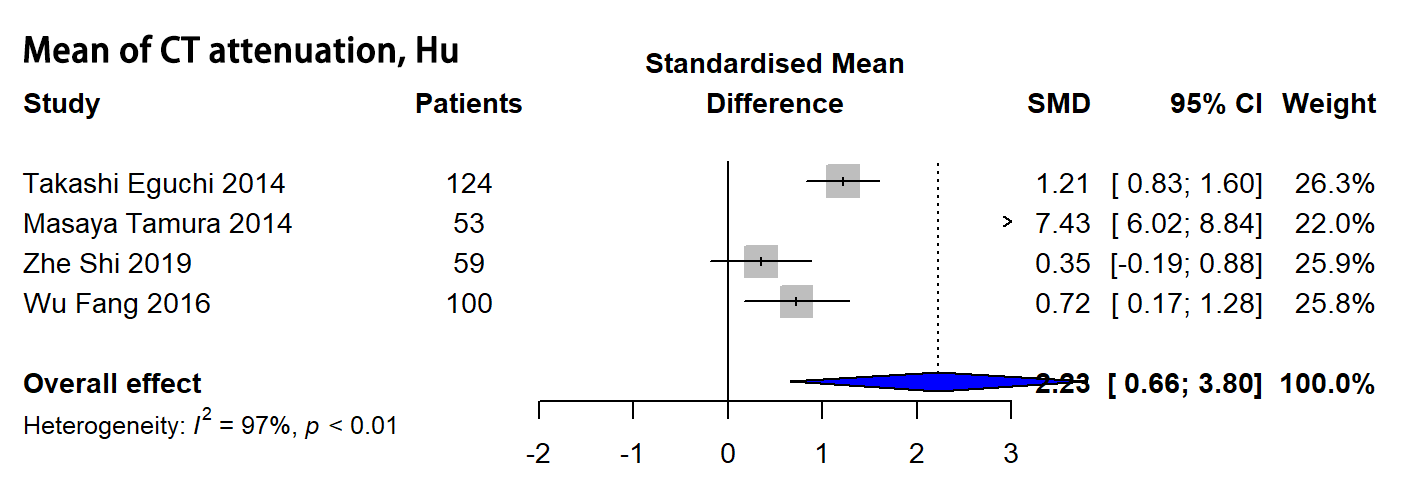

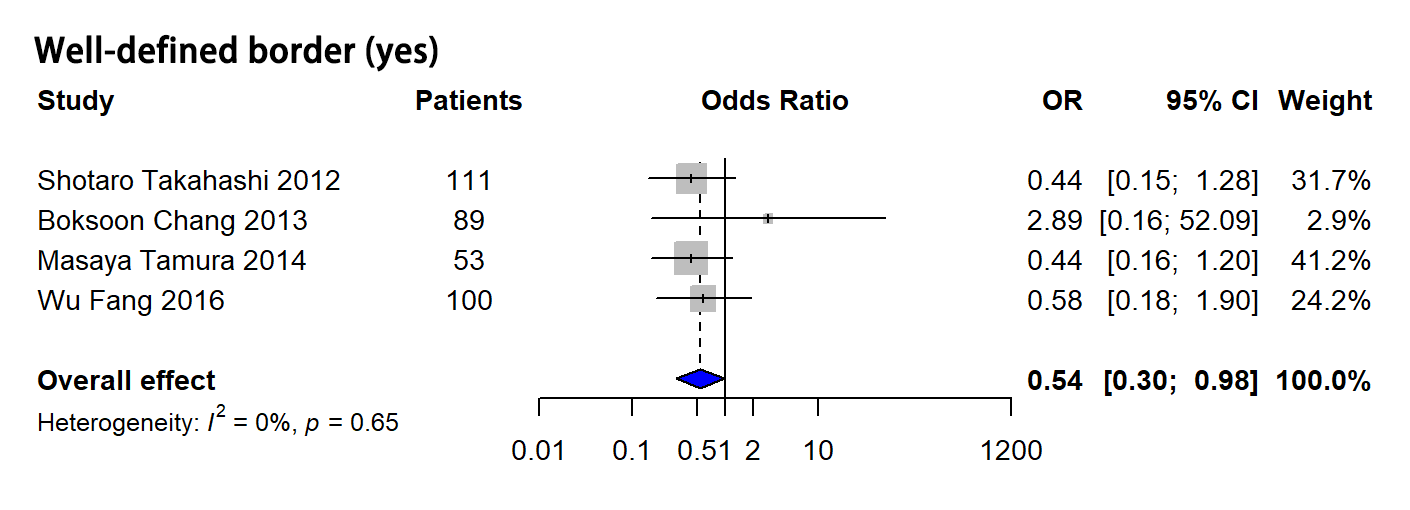

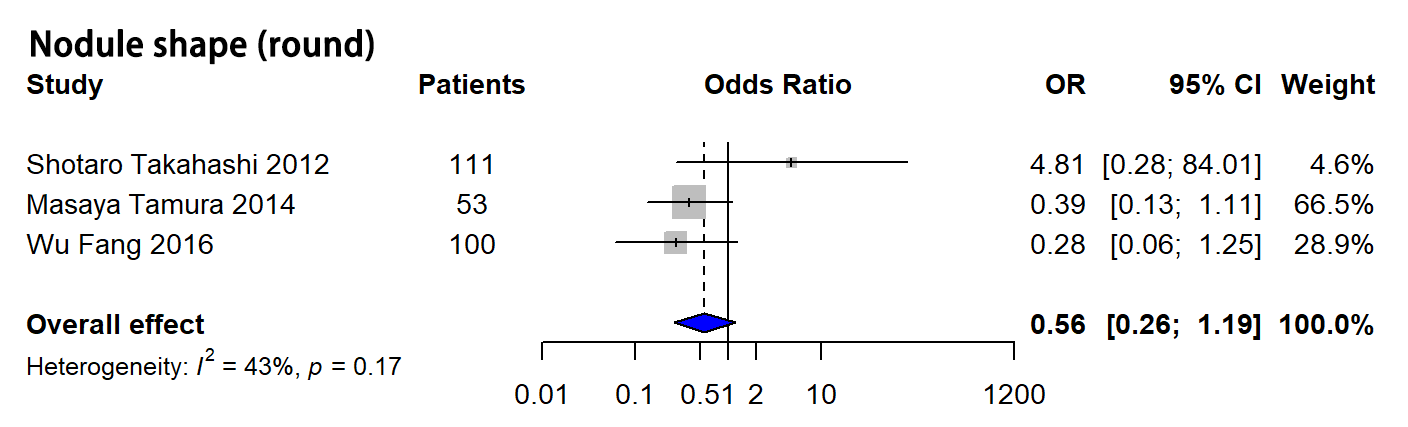

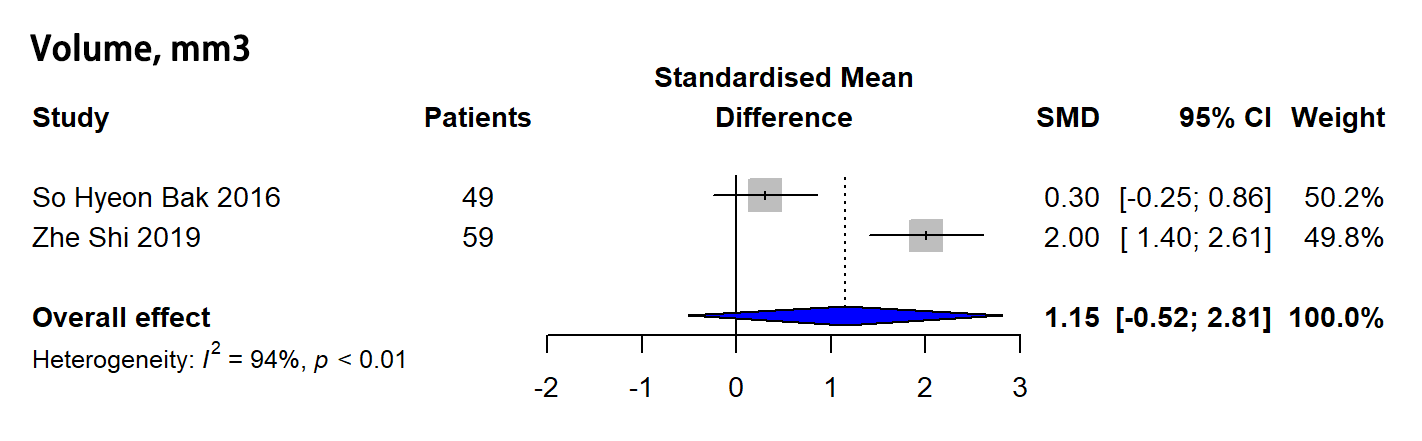

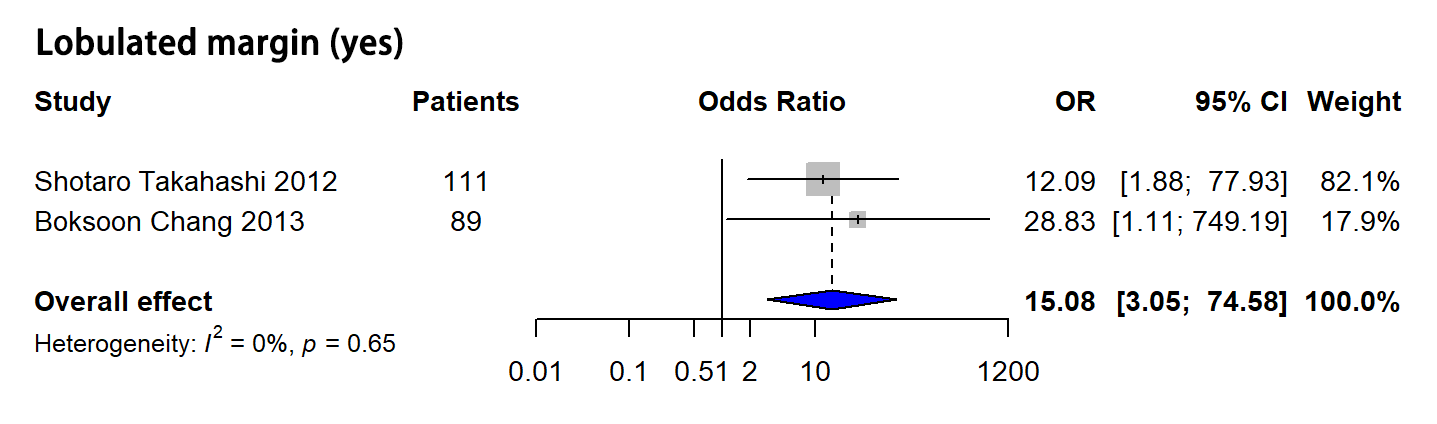

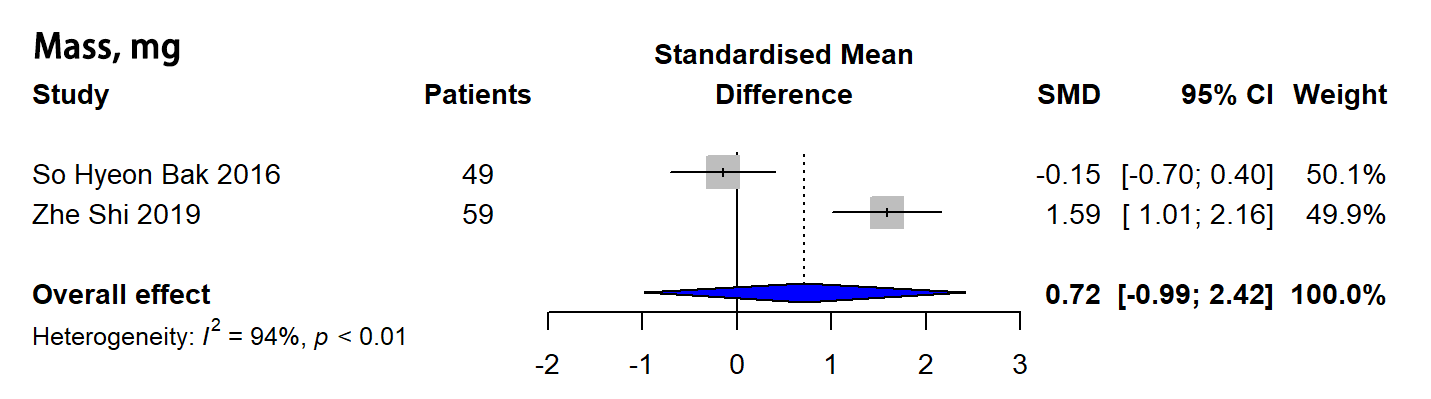


Figure S2. Forest plots of 16 features analyzed based on univariate analysis results in pGGN.


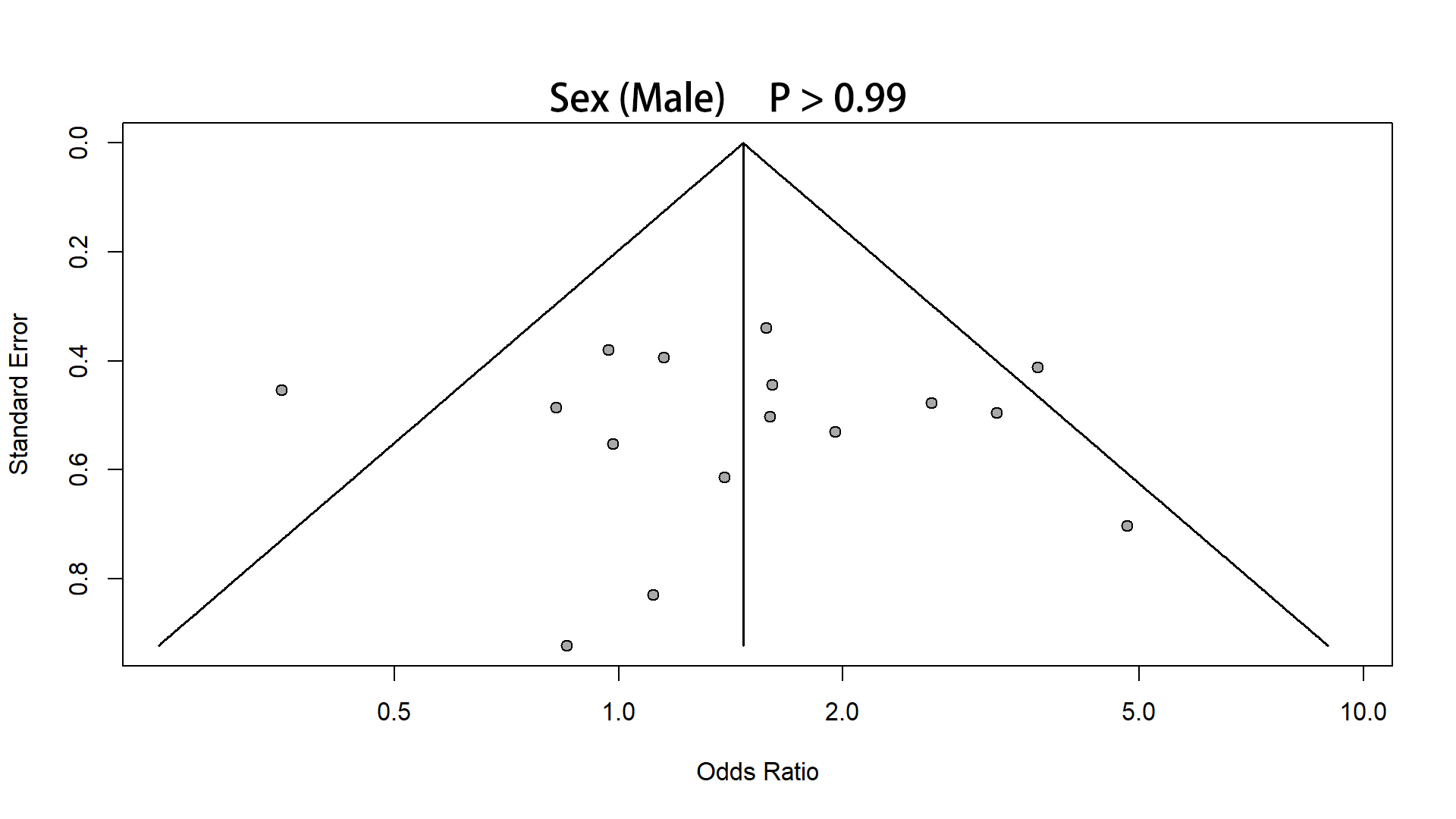

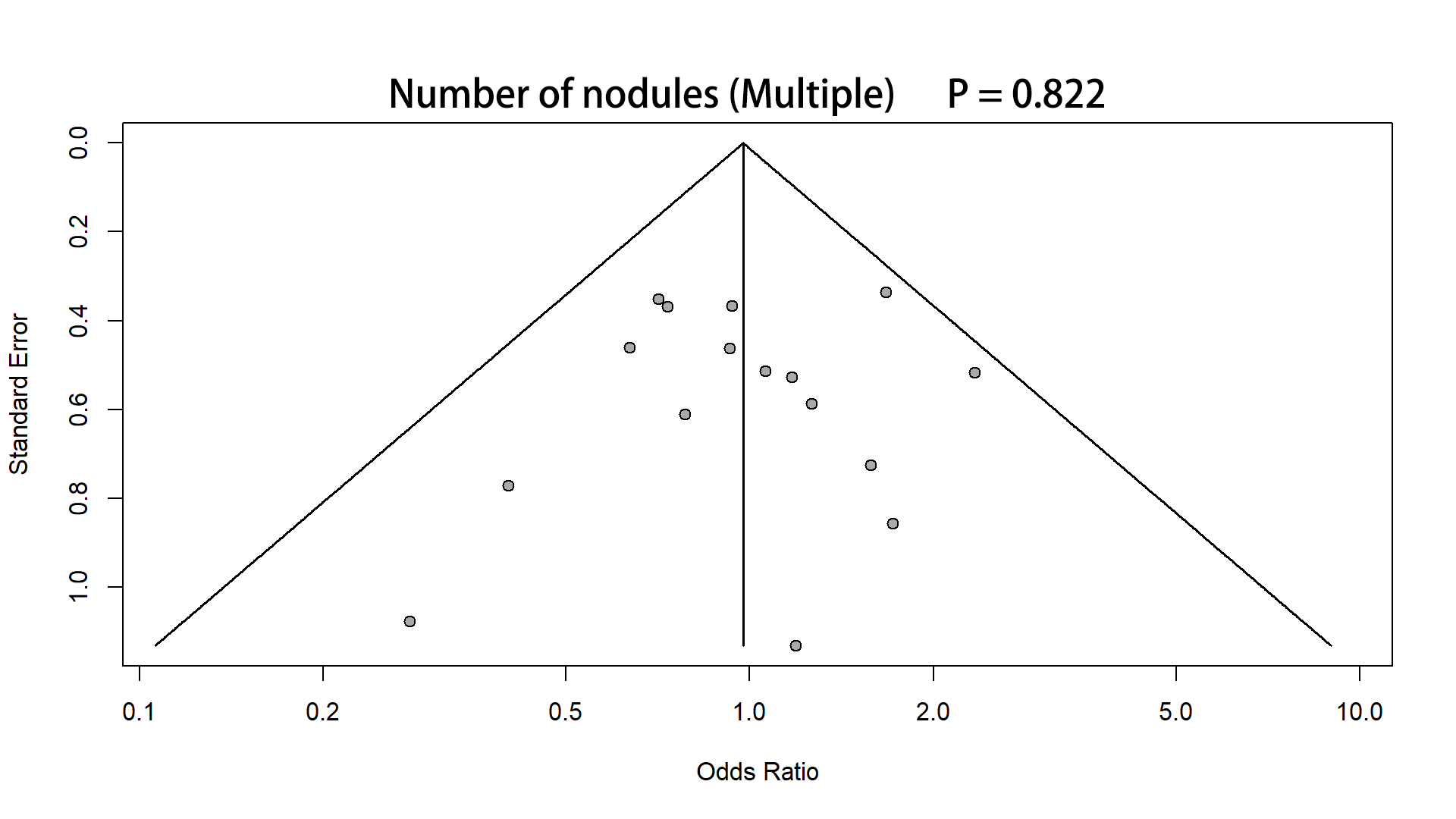

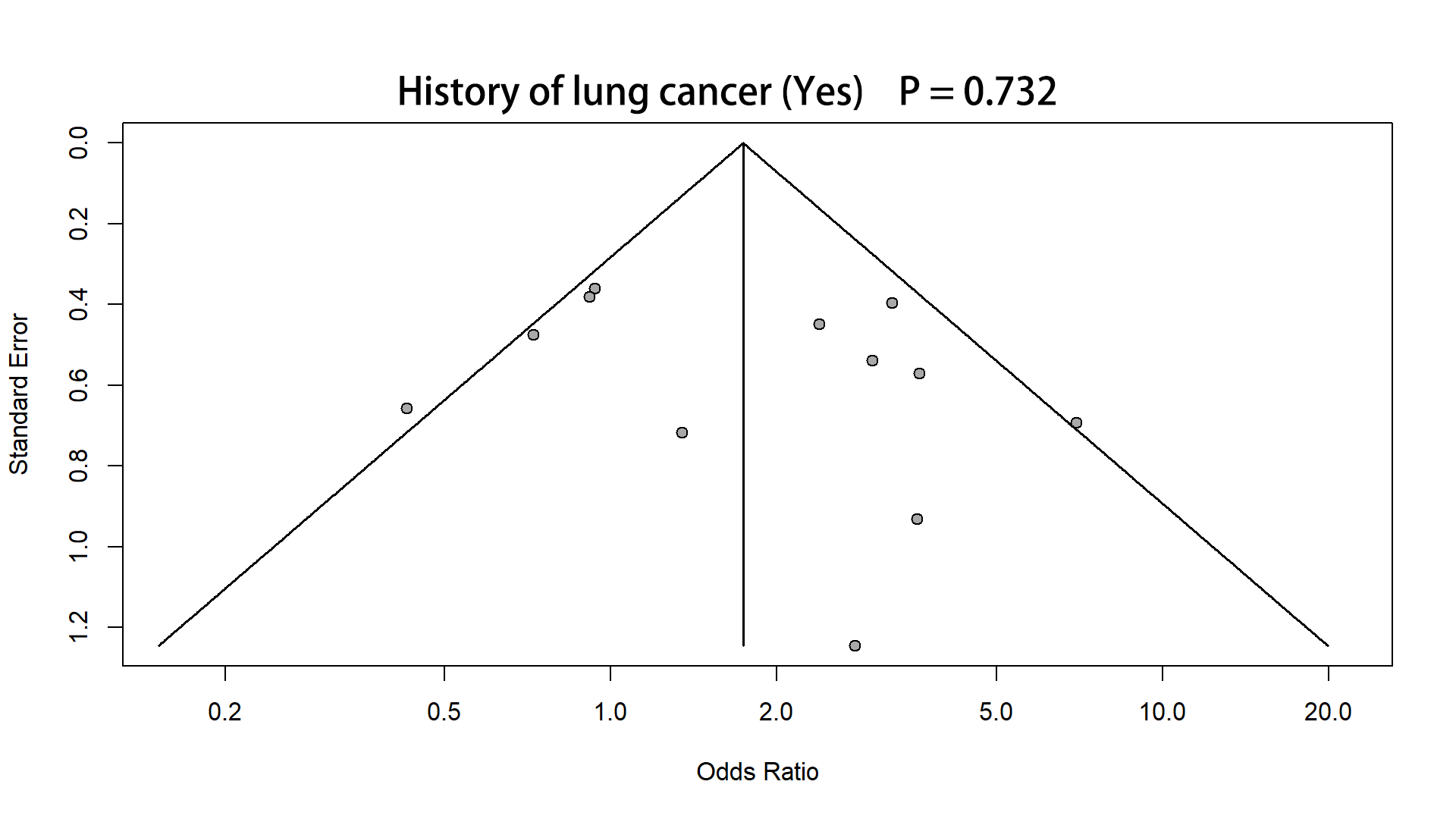

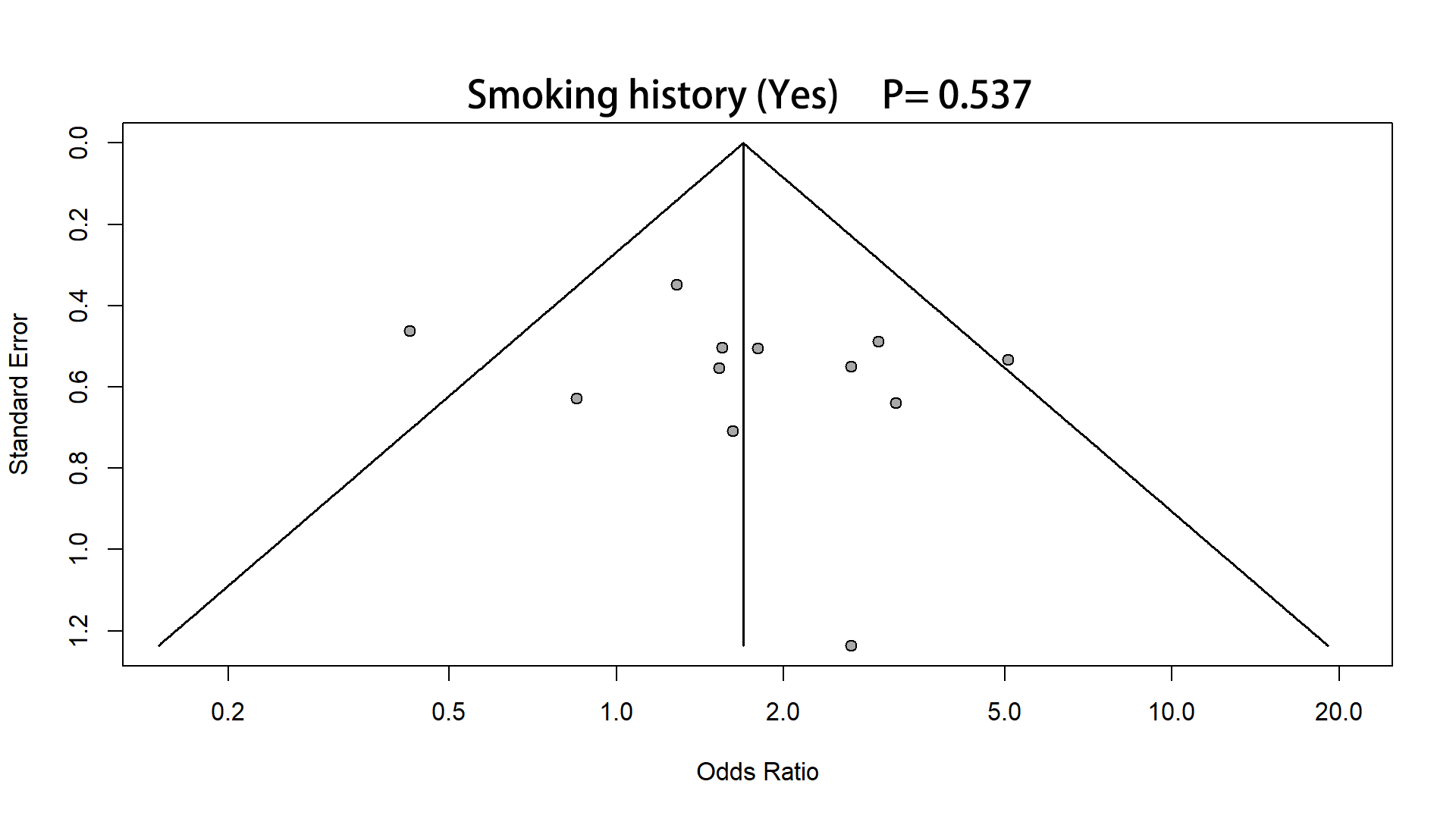

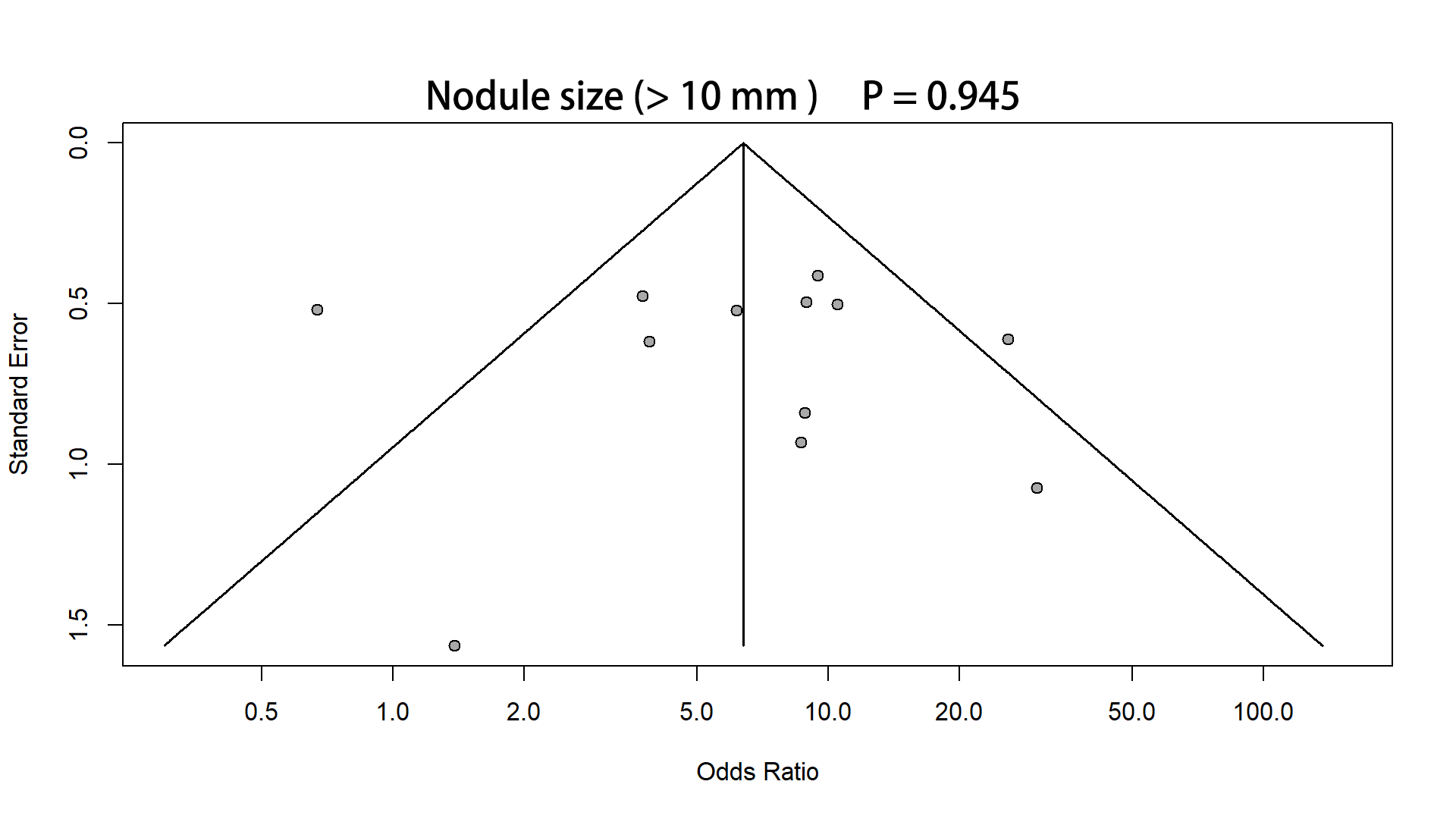

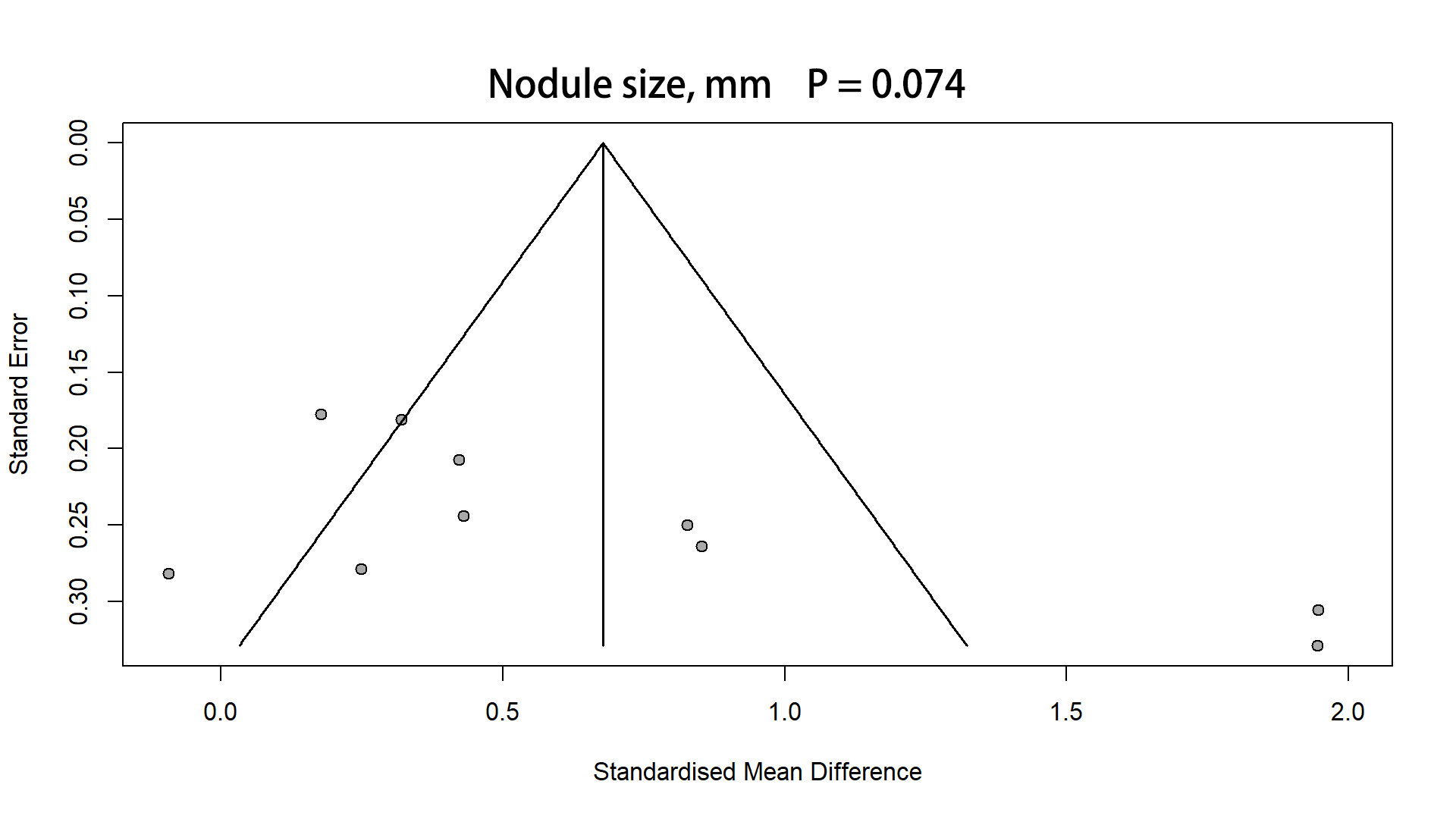

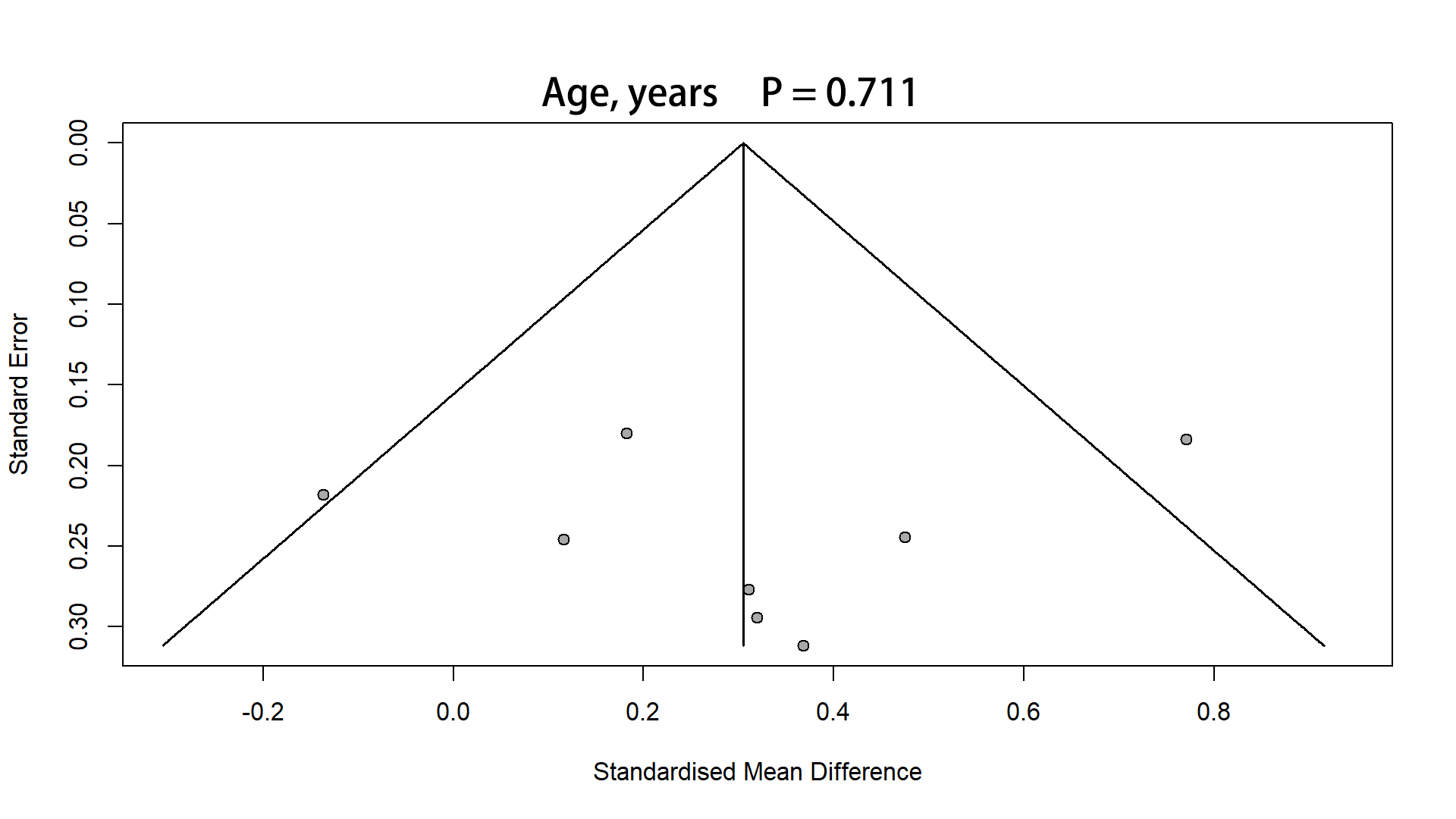

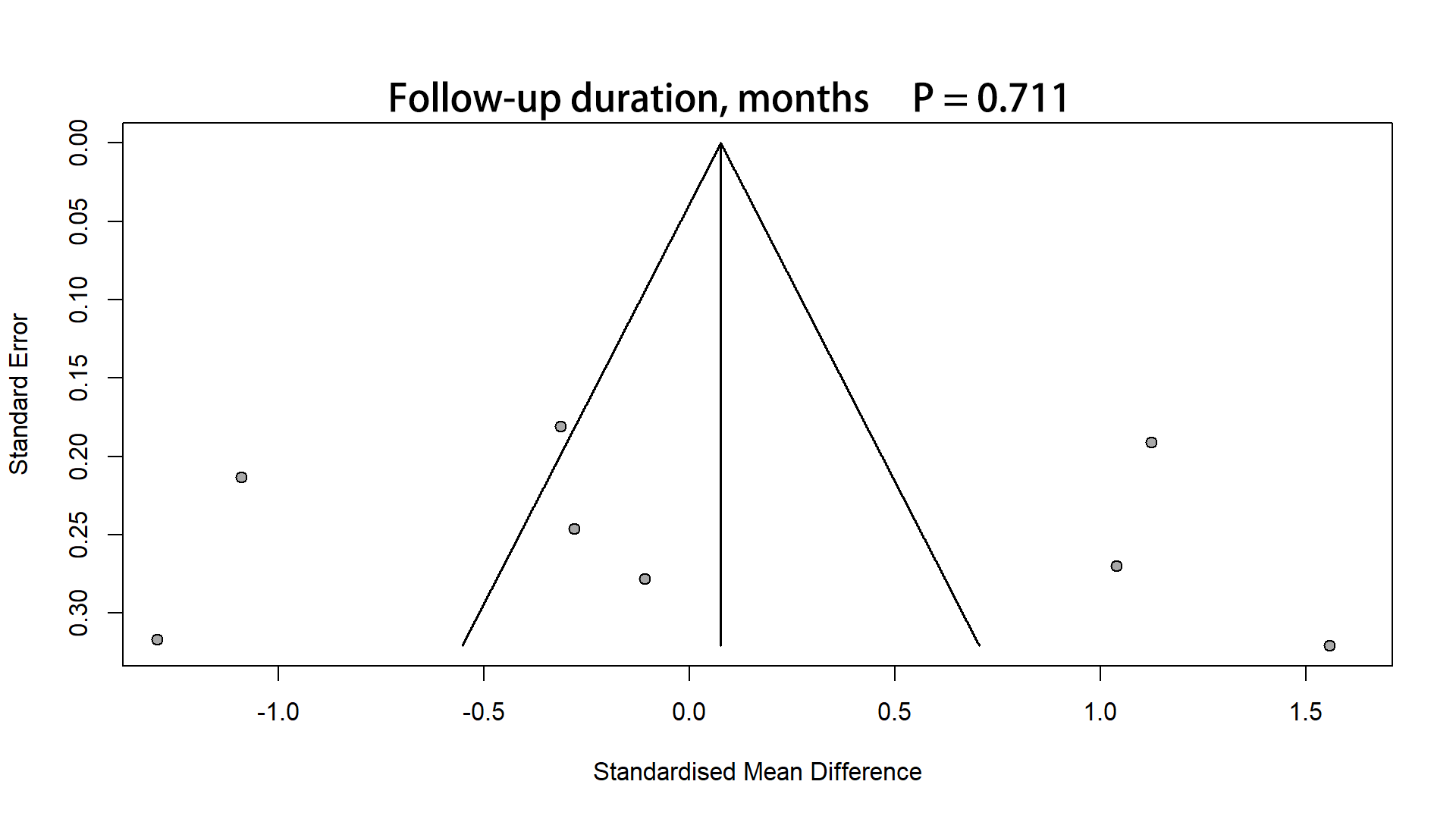

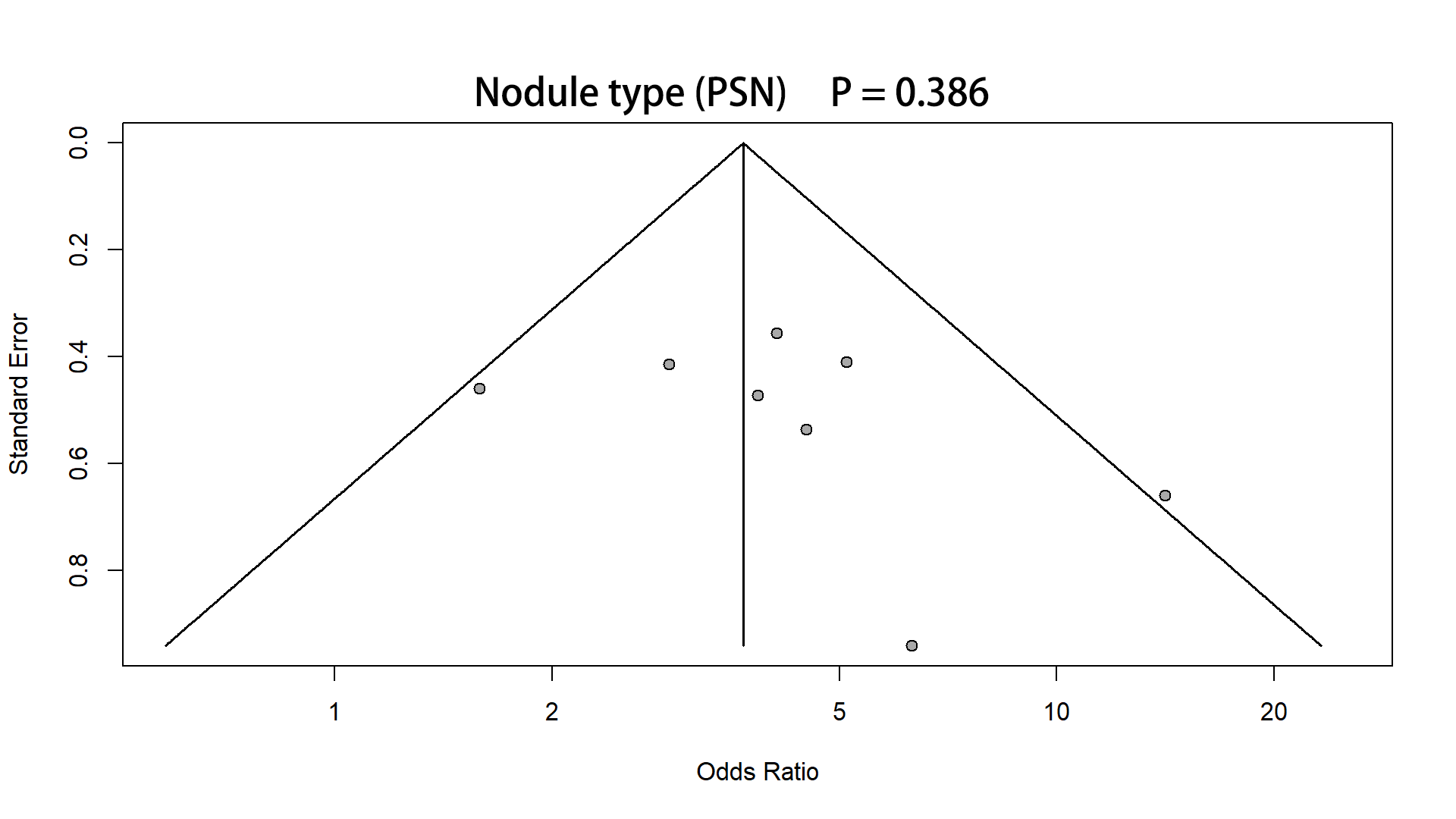

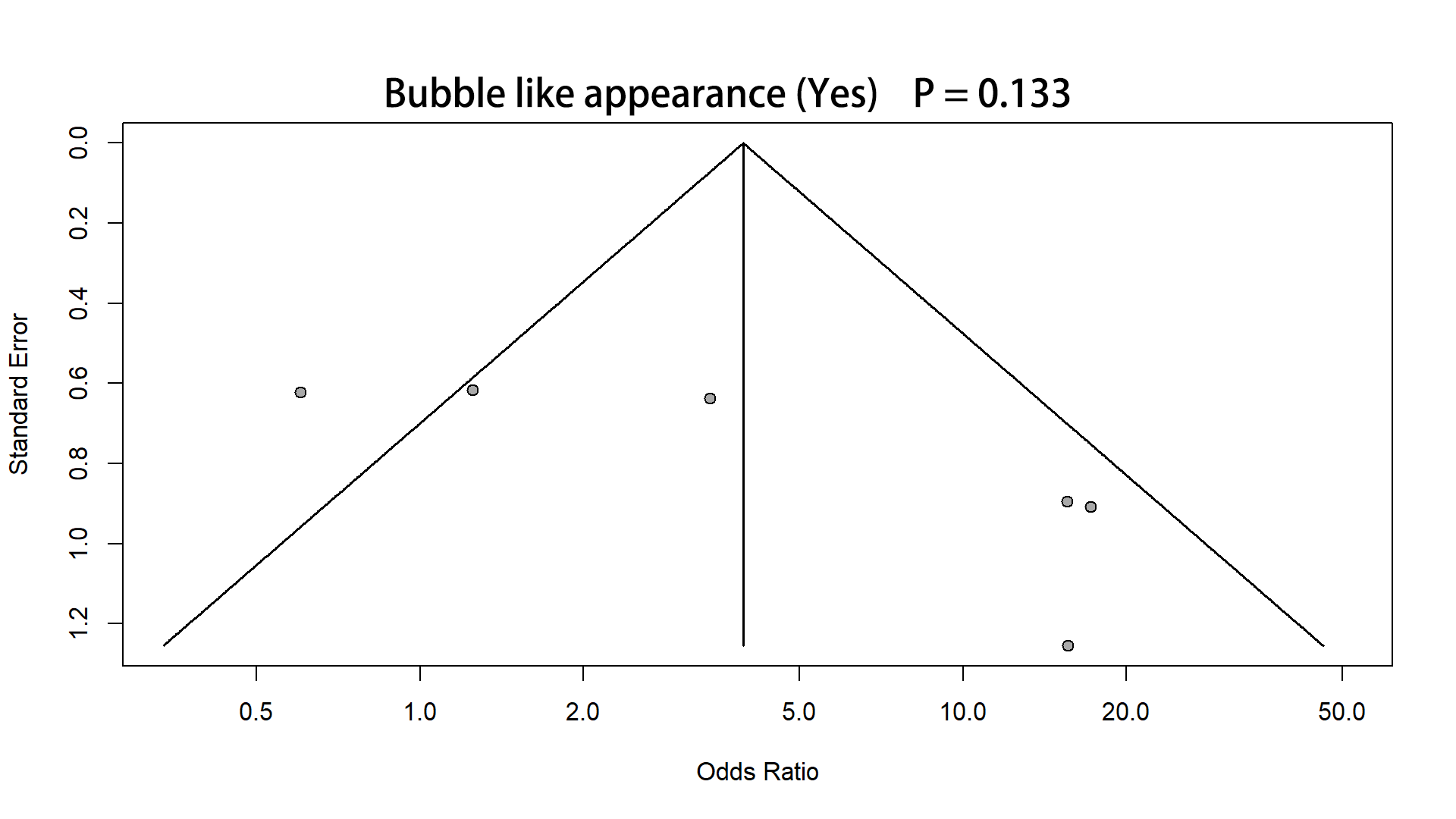

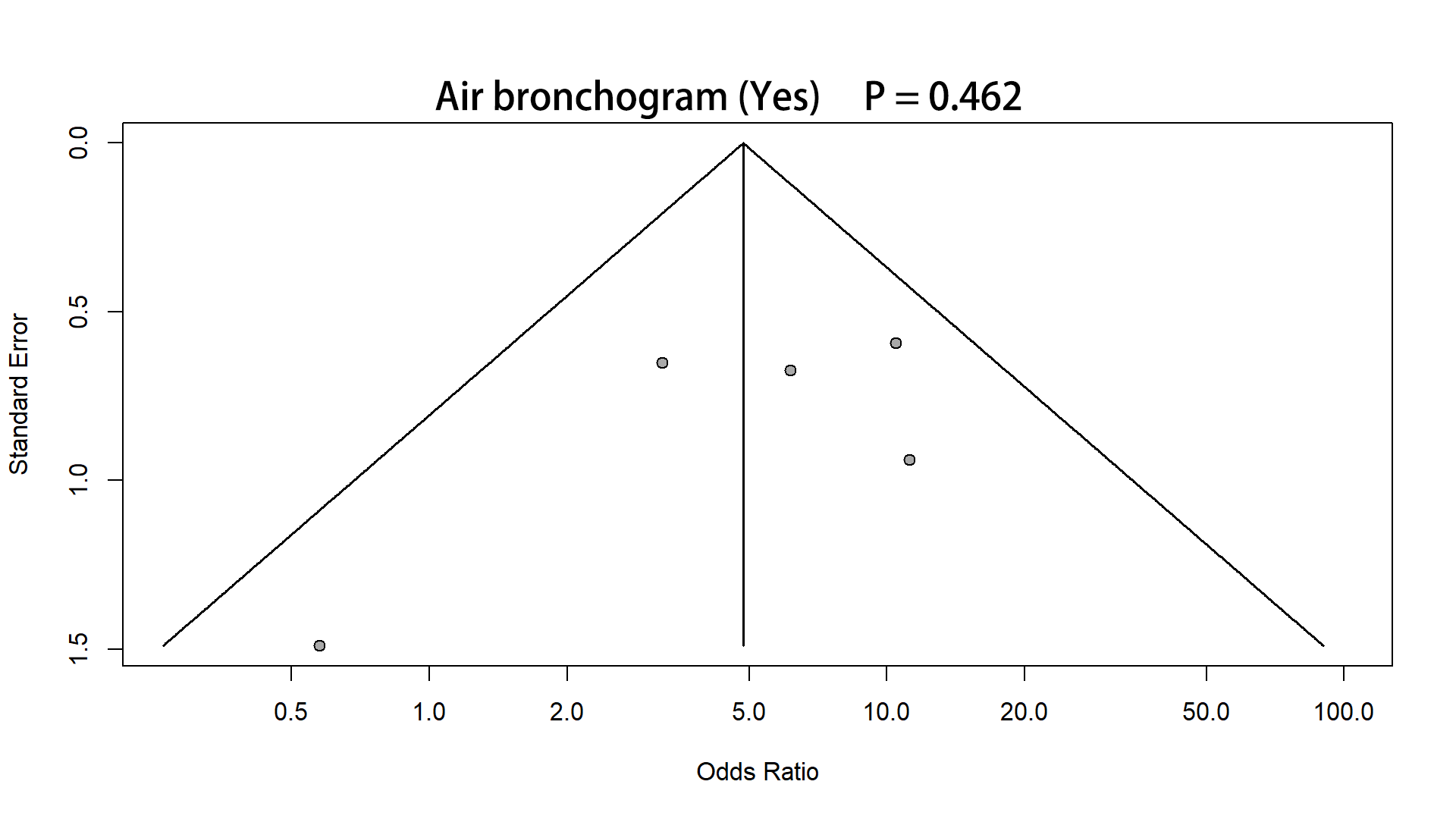

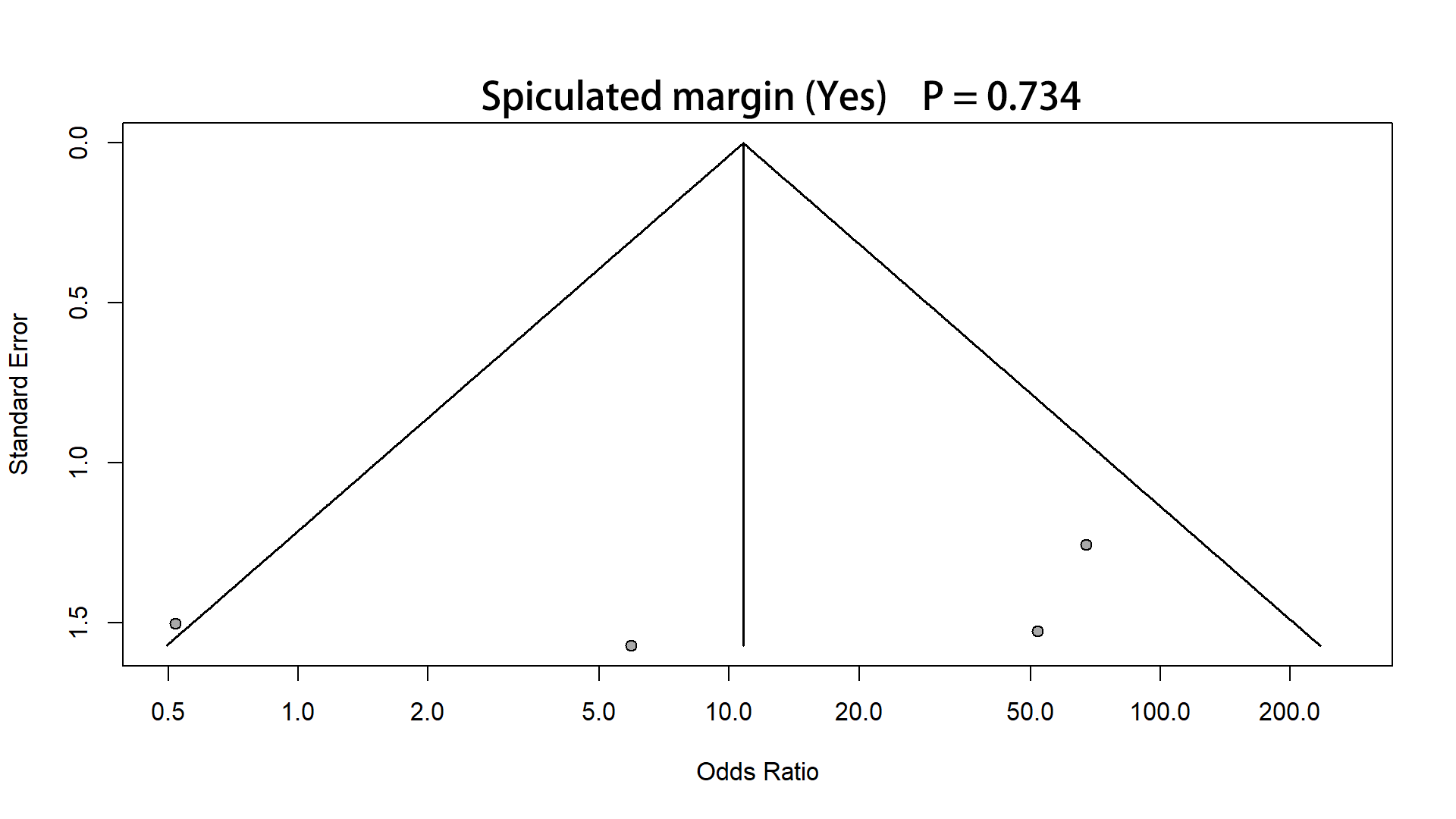

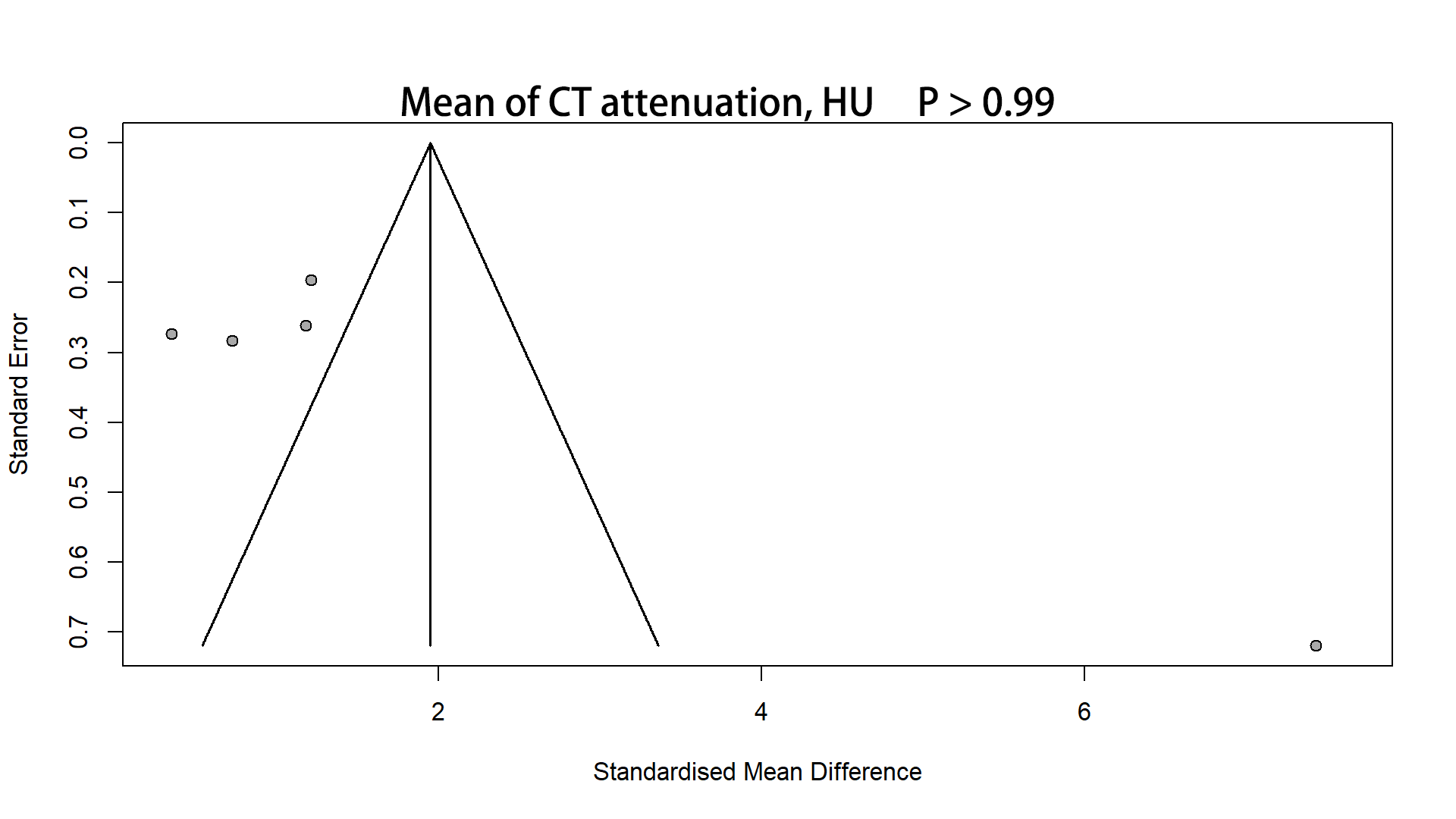

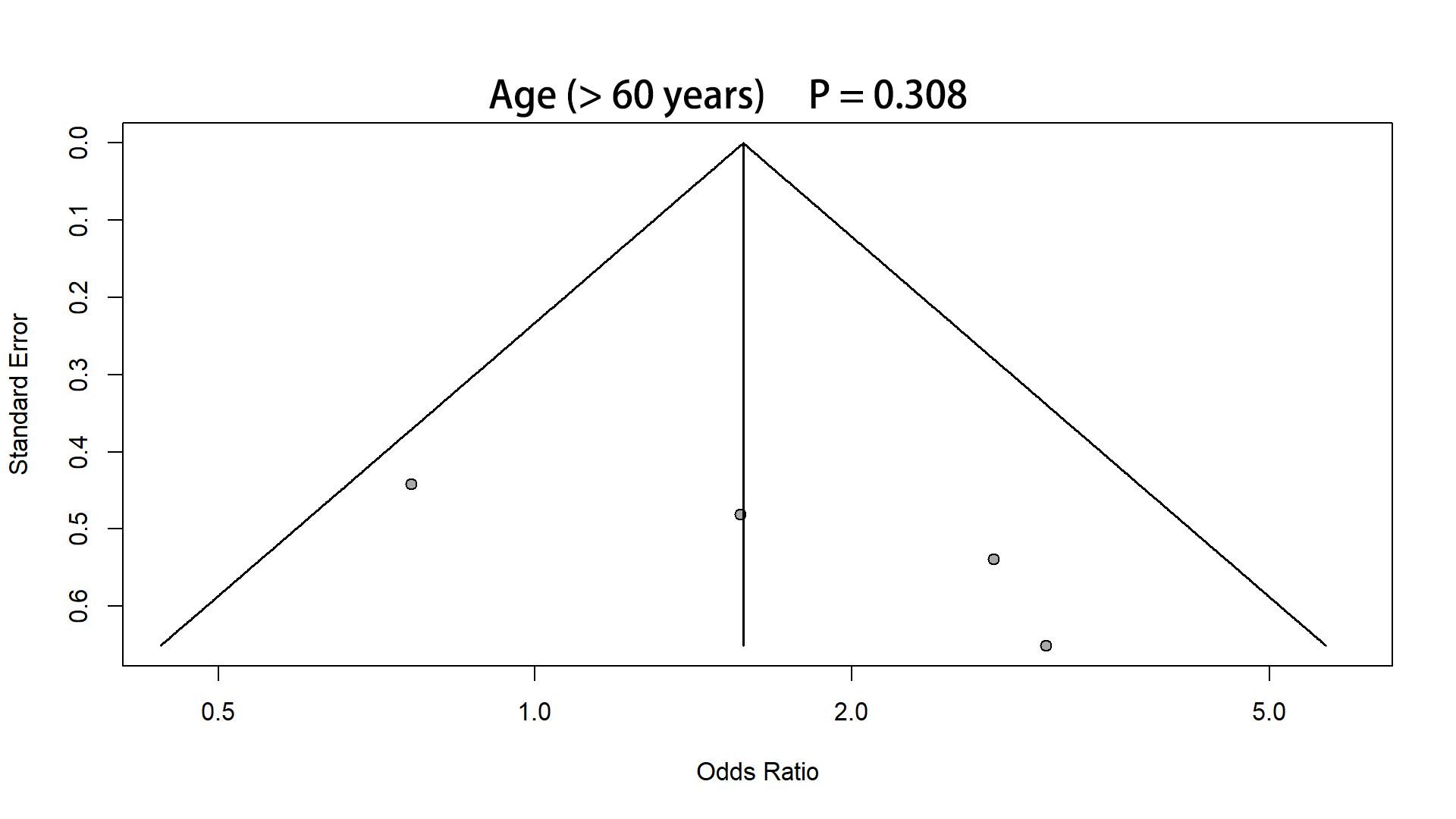

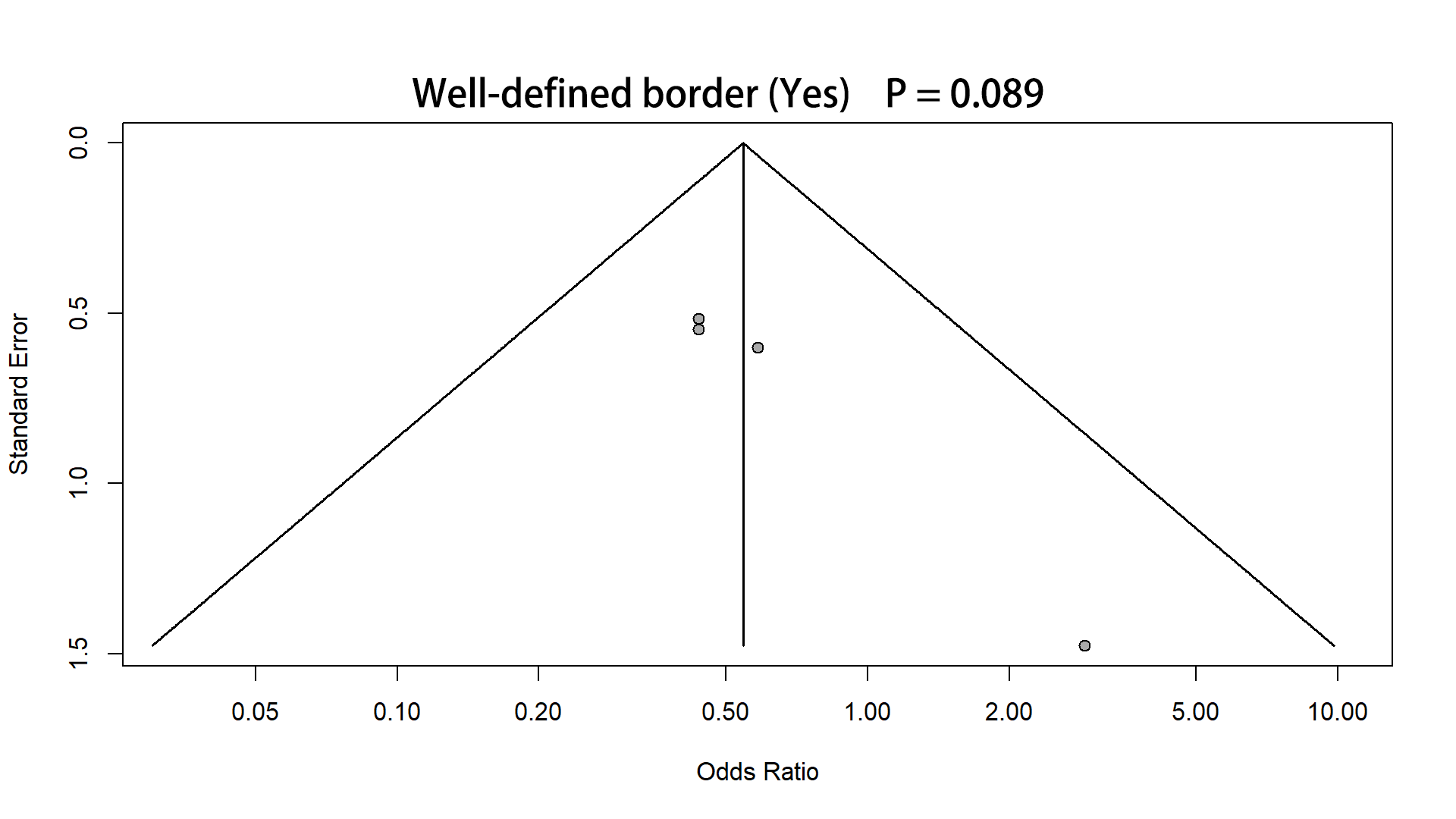

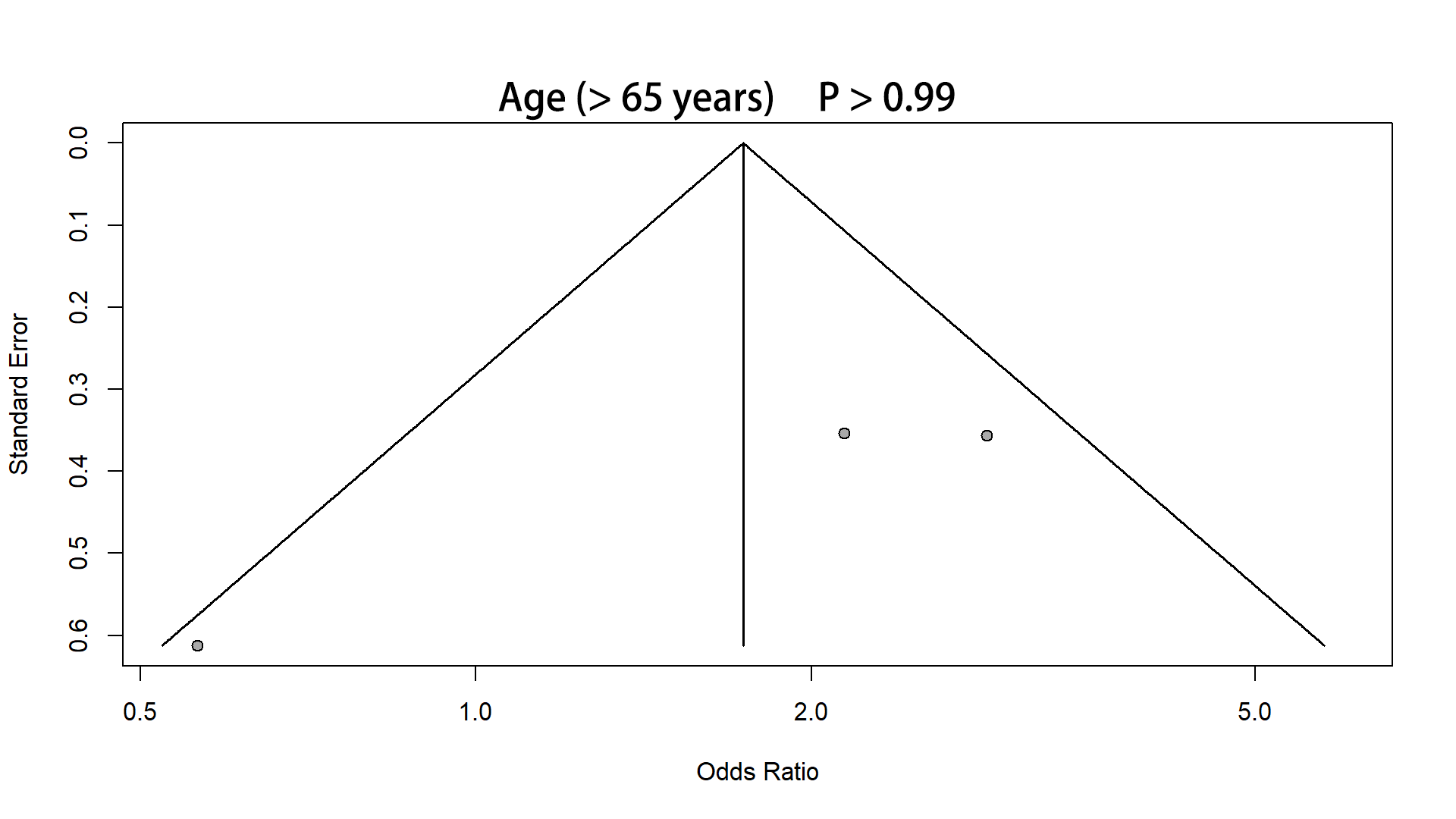

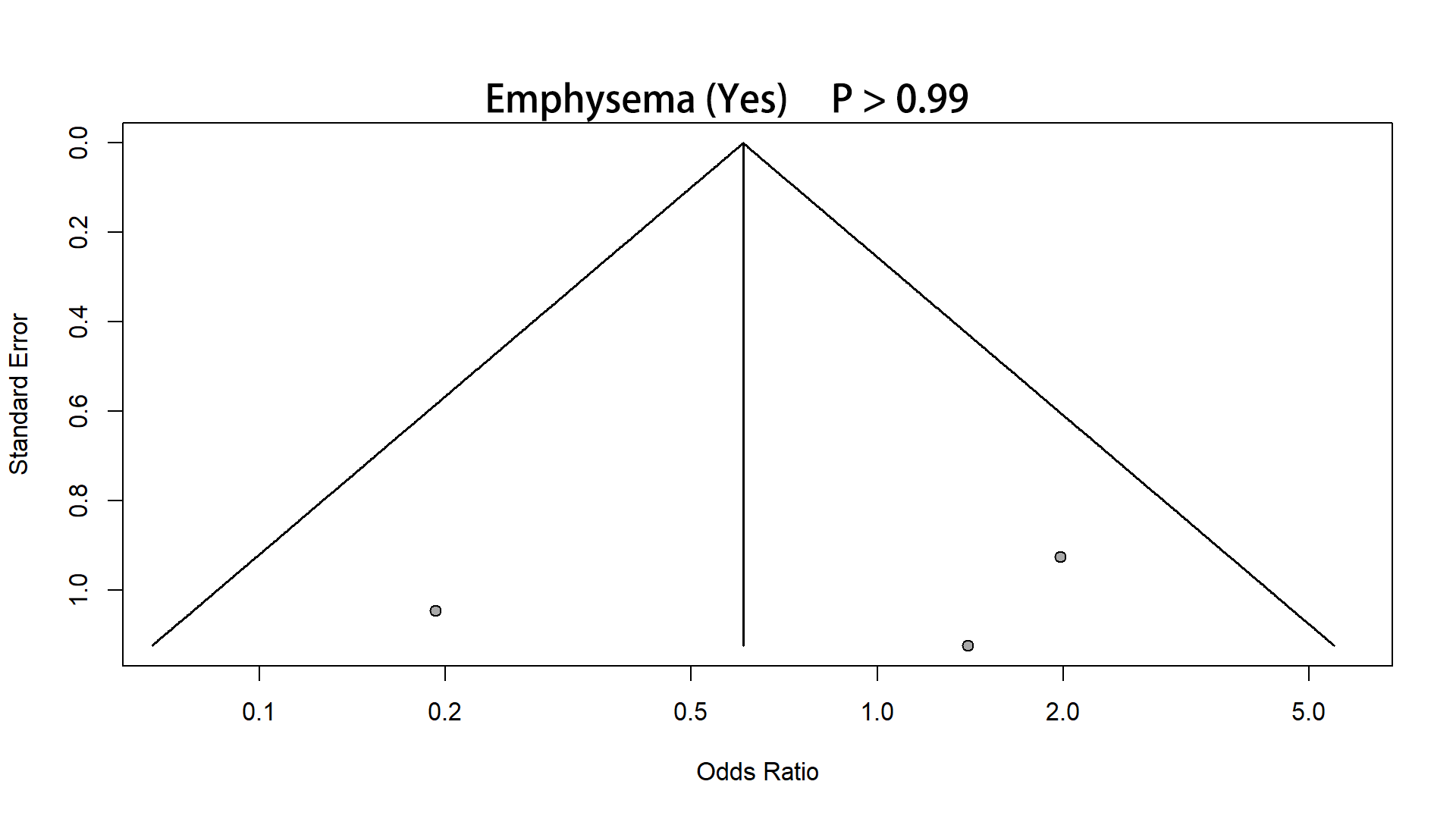

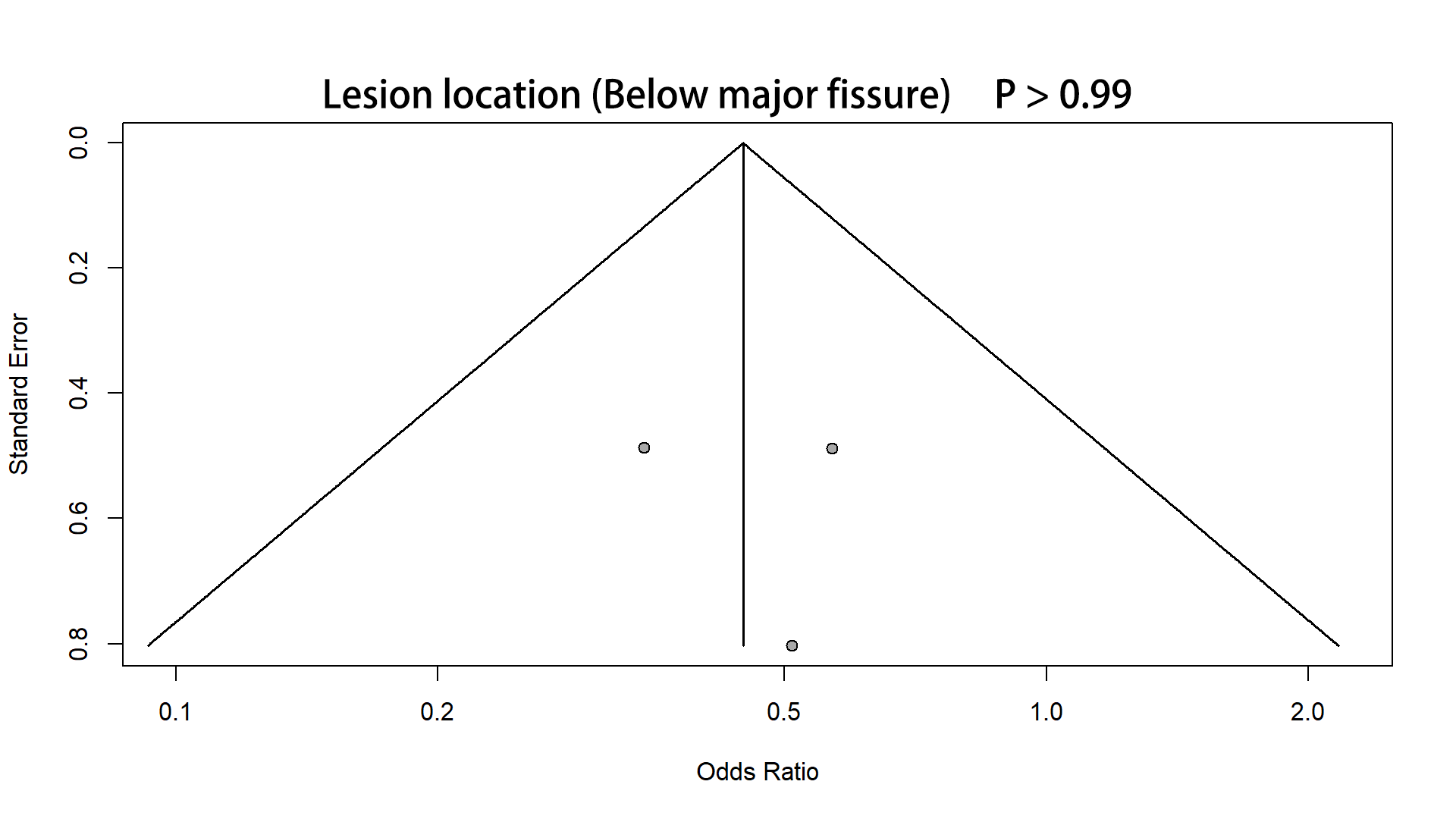

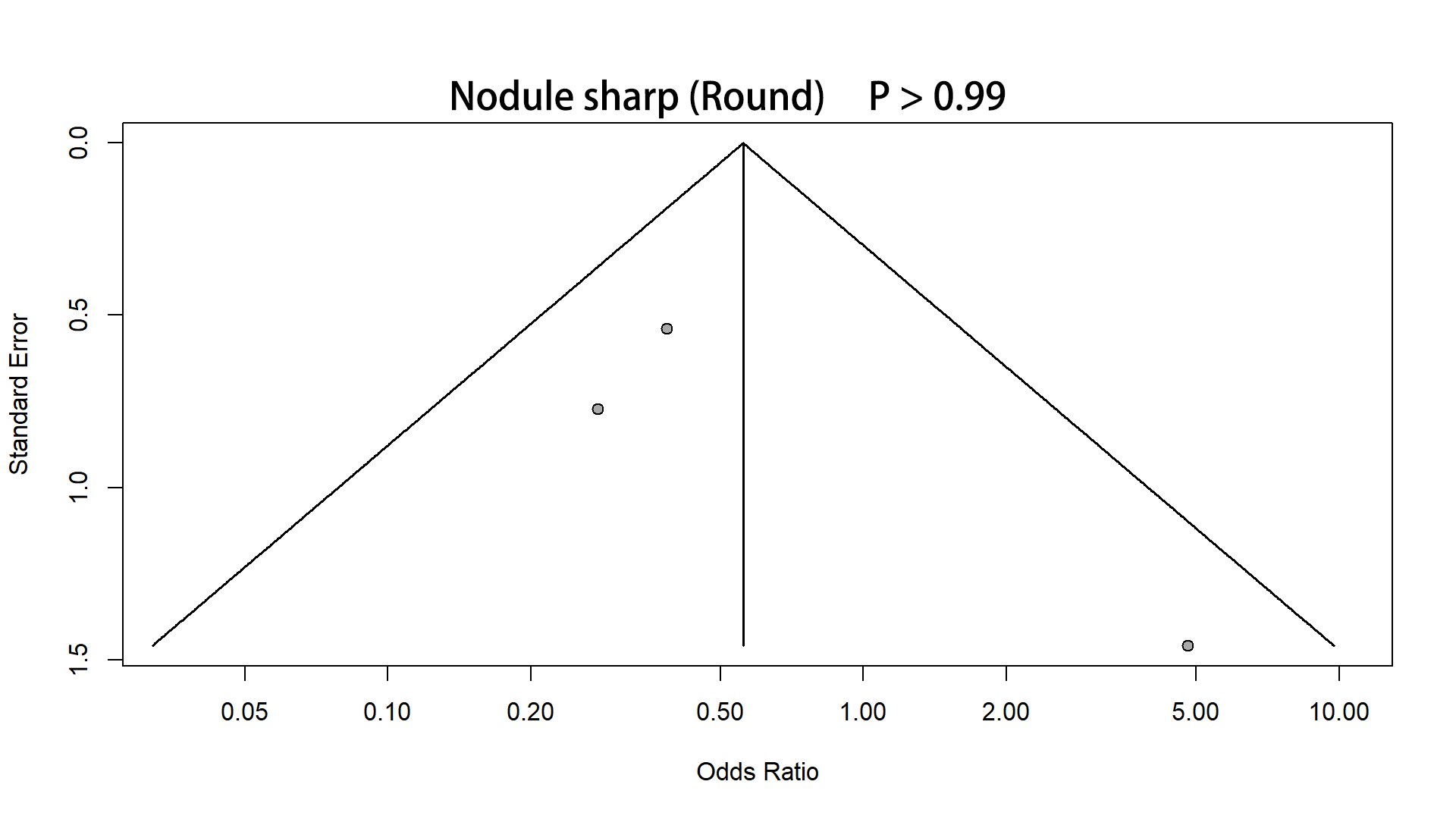

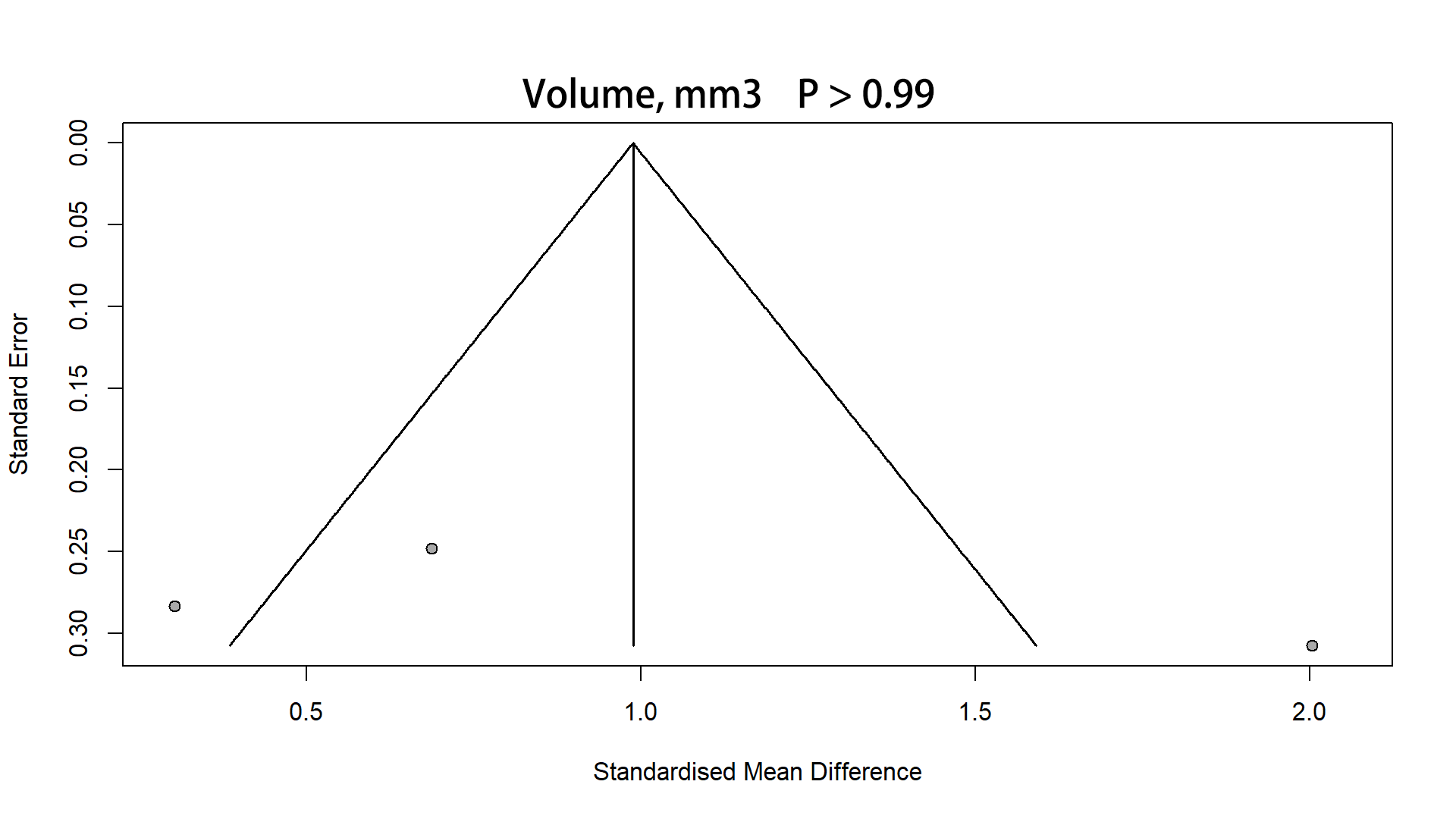

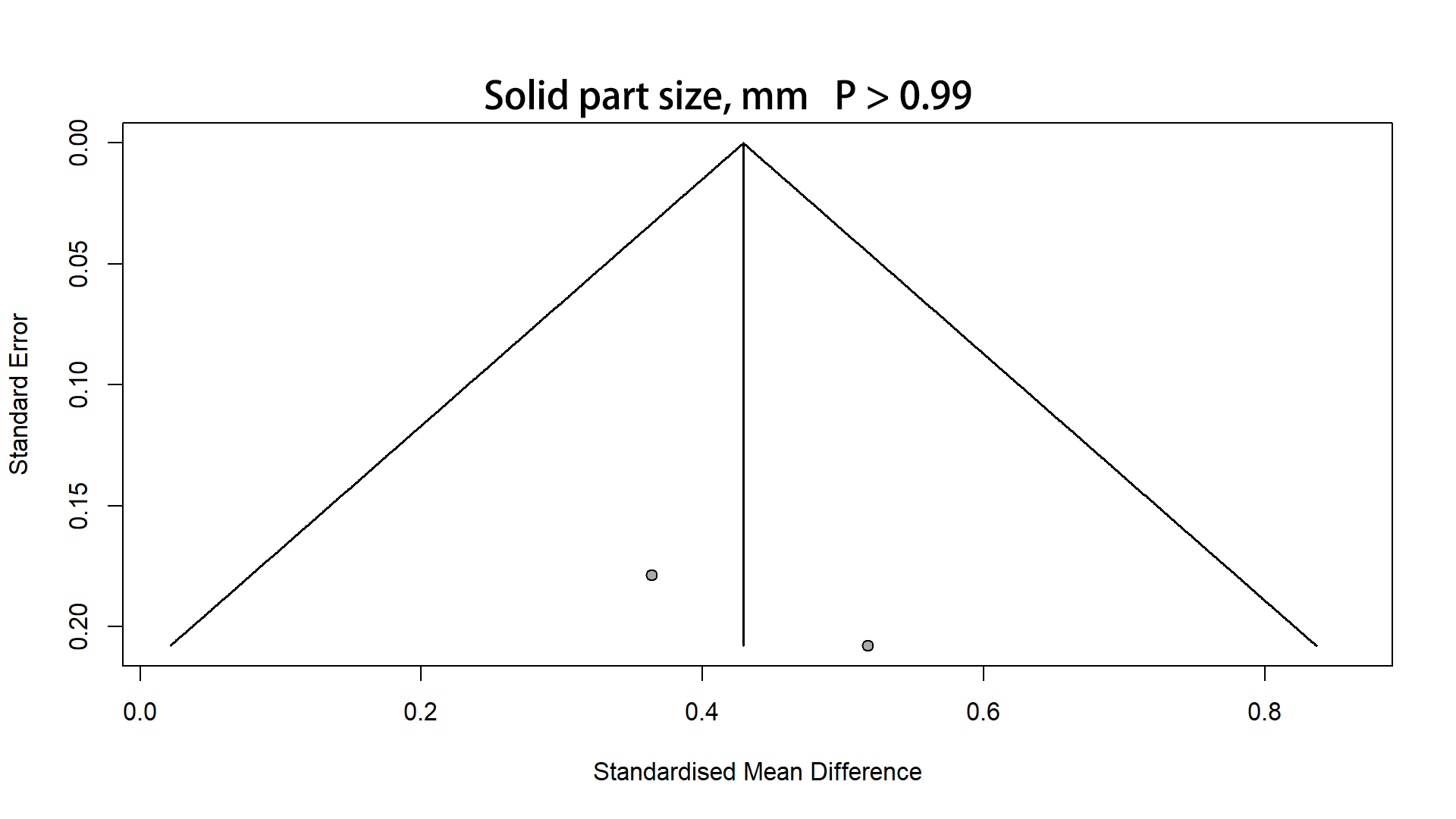

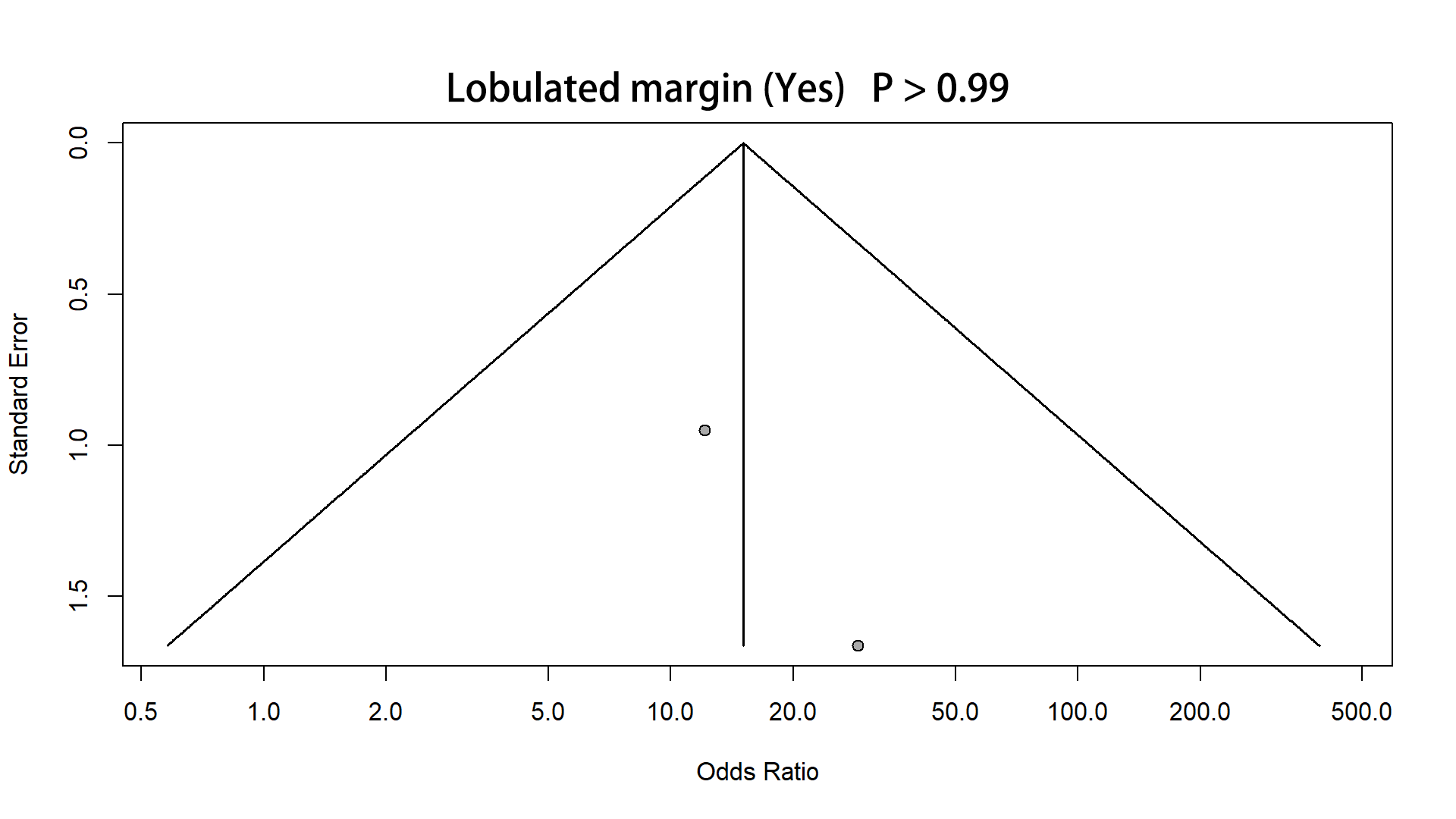

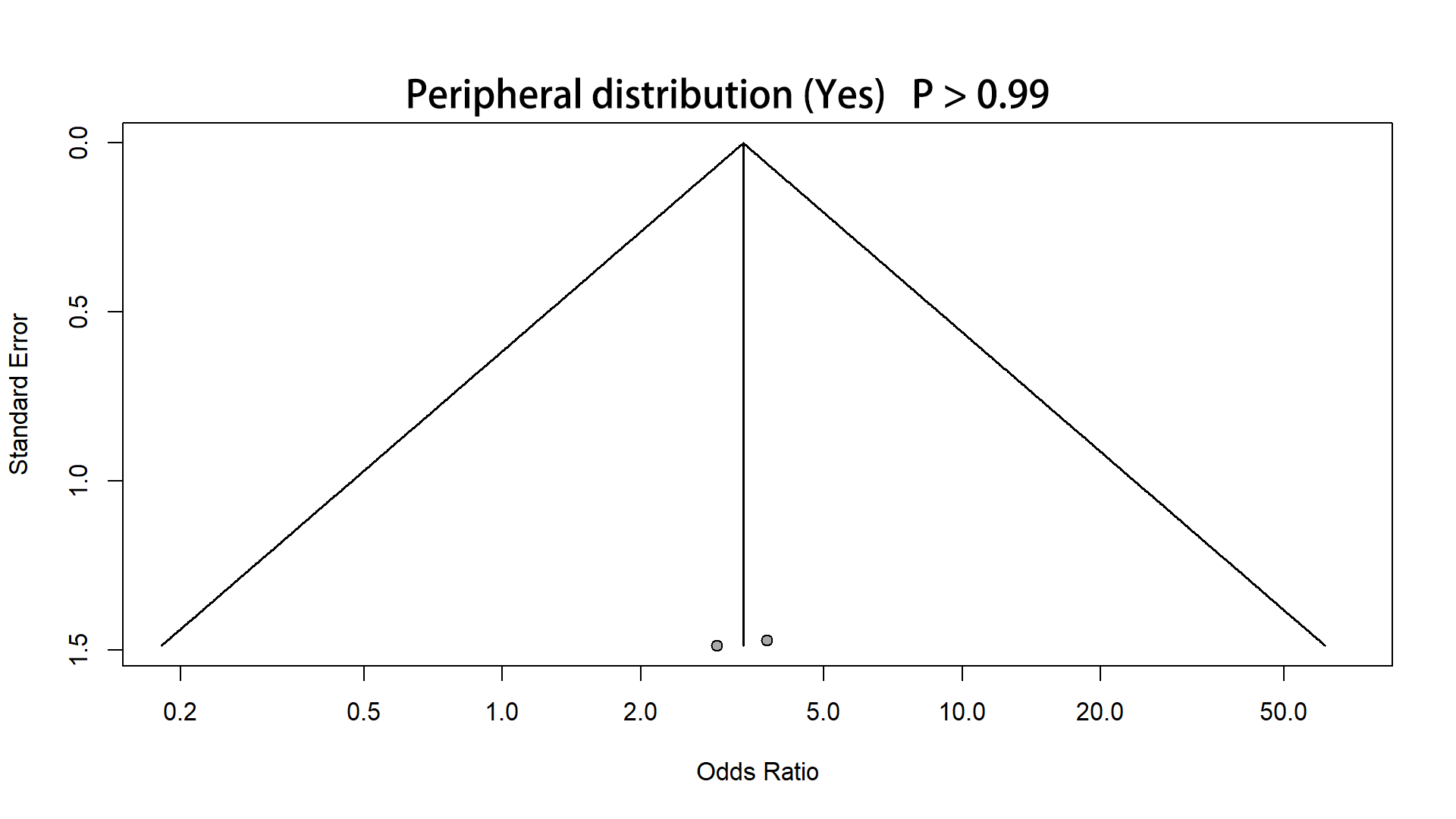

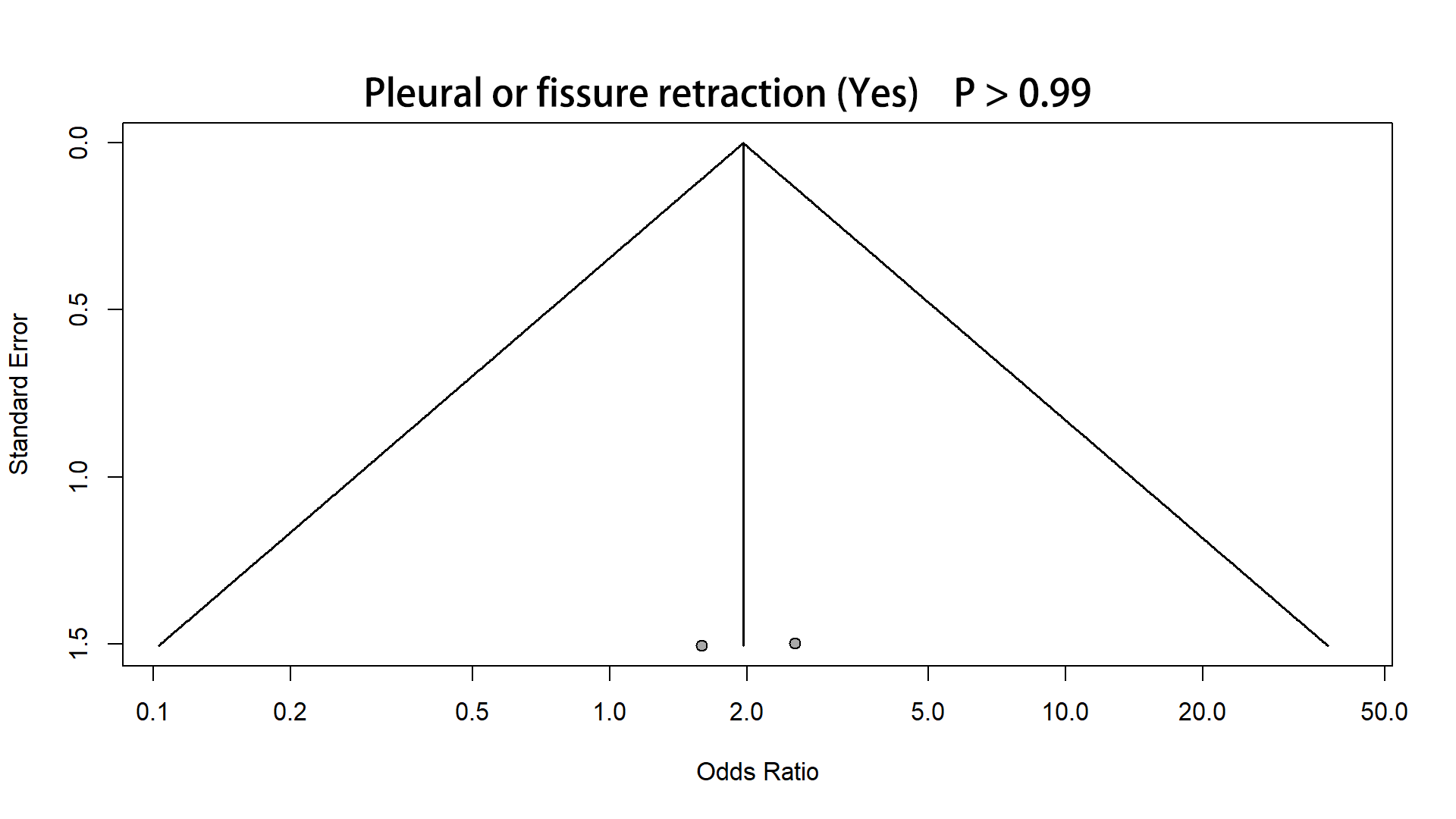

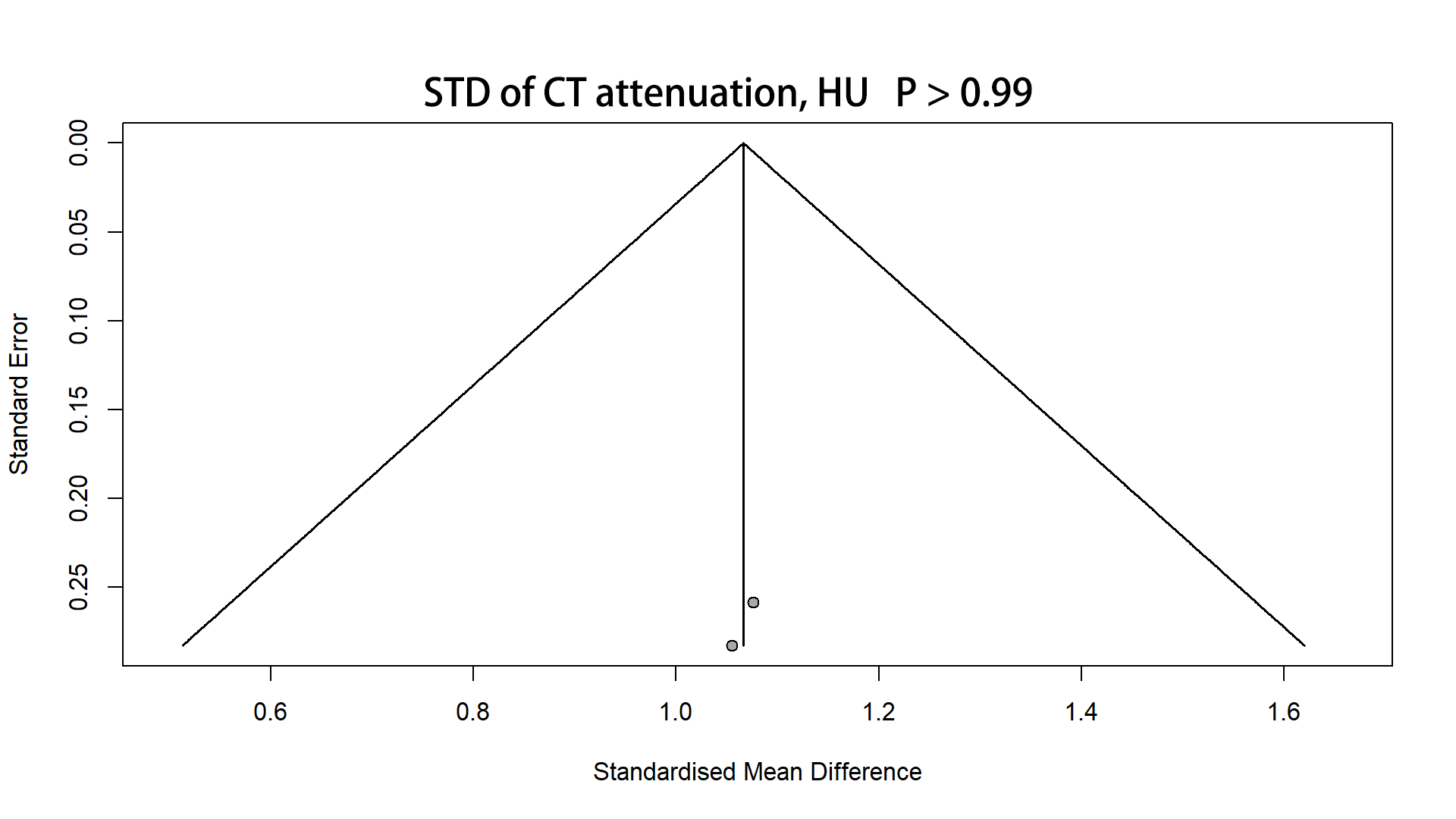

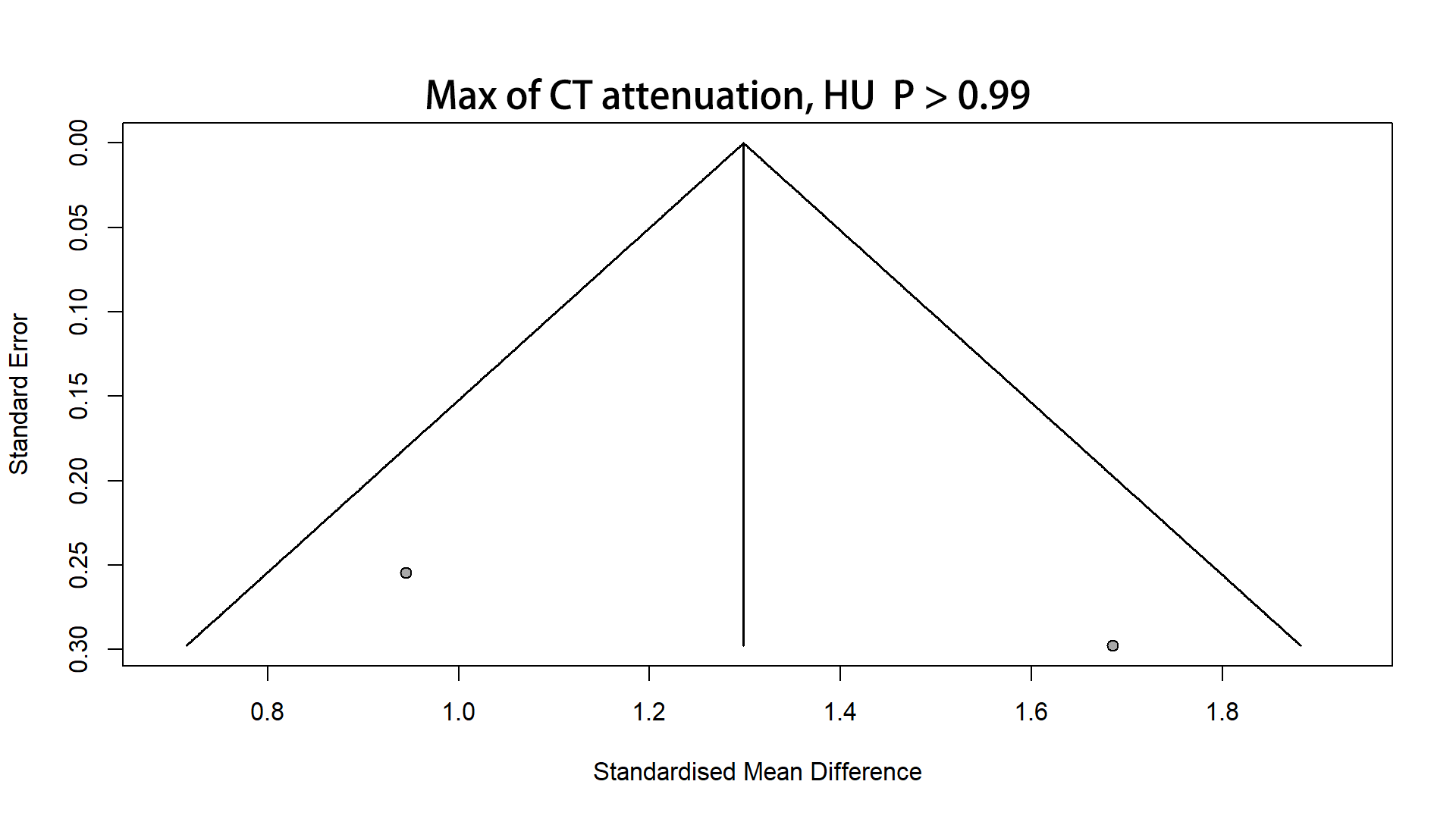

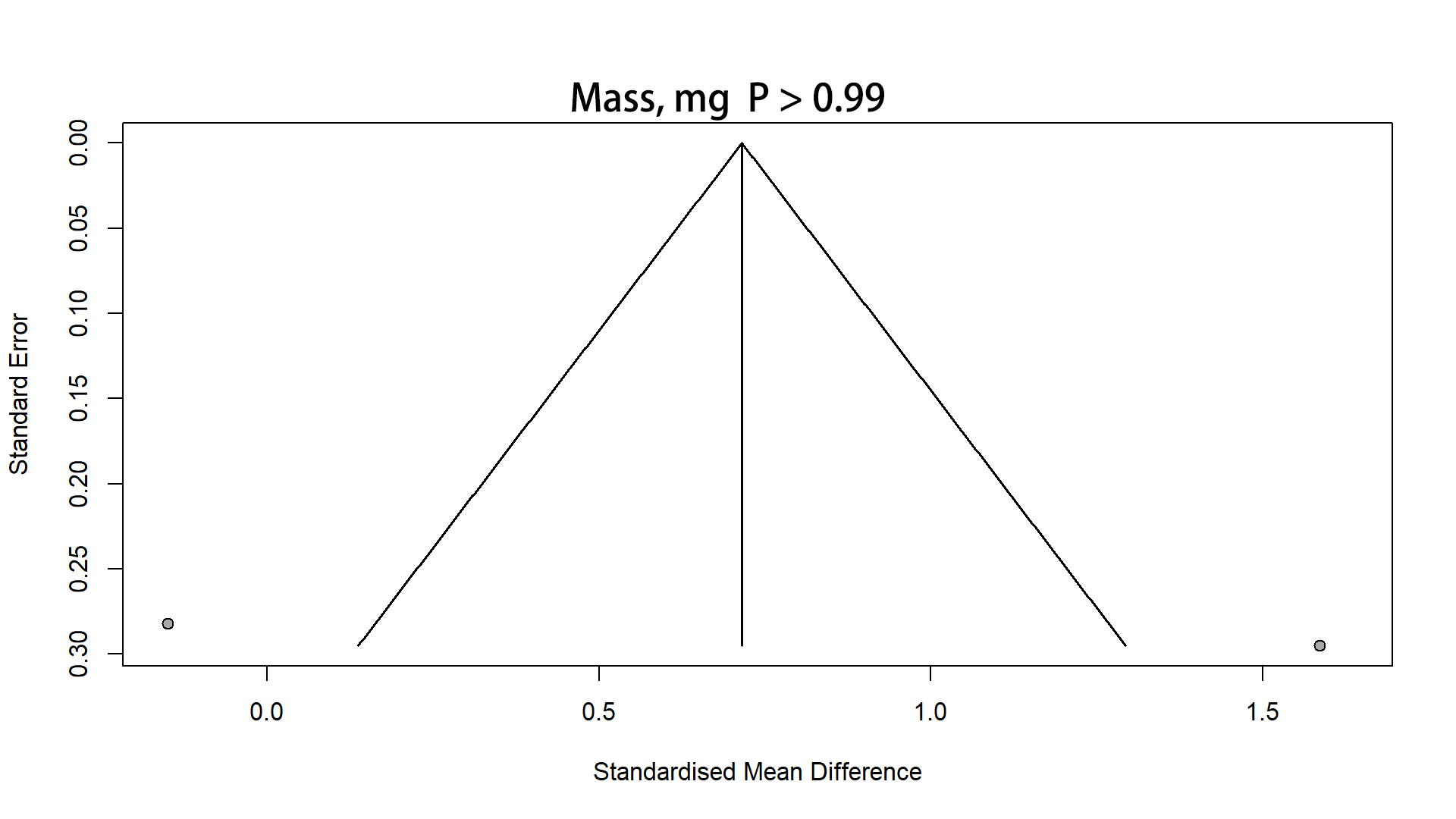

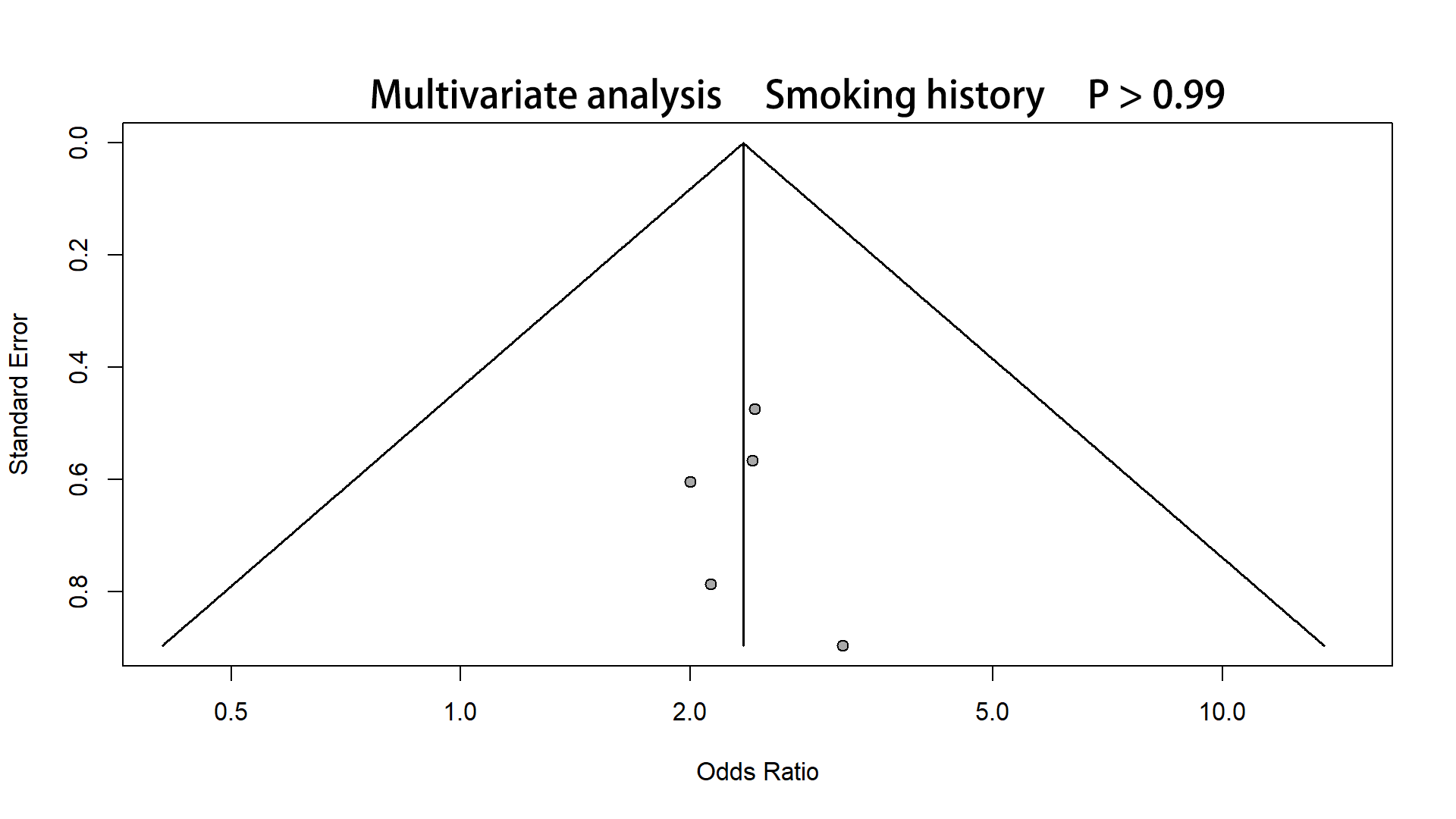

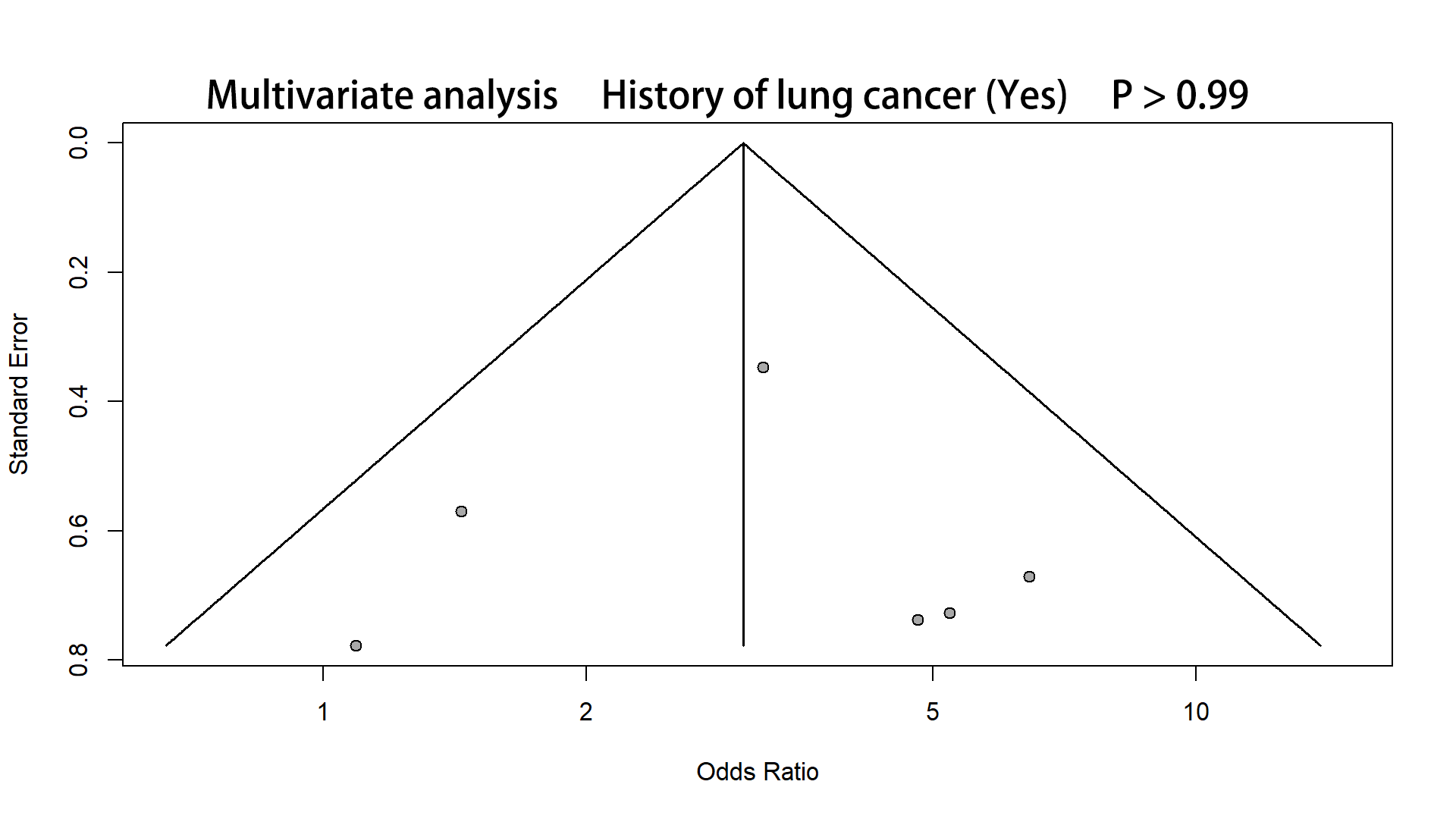

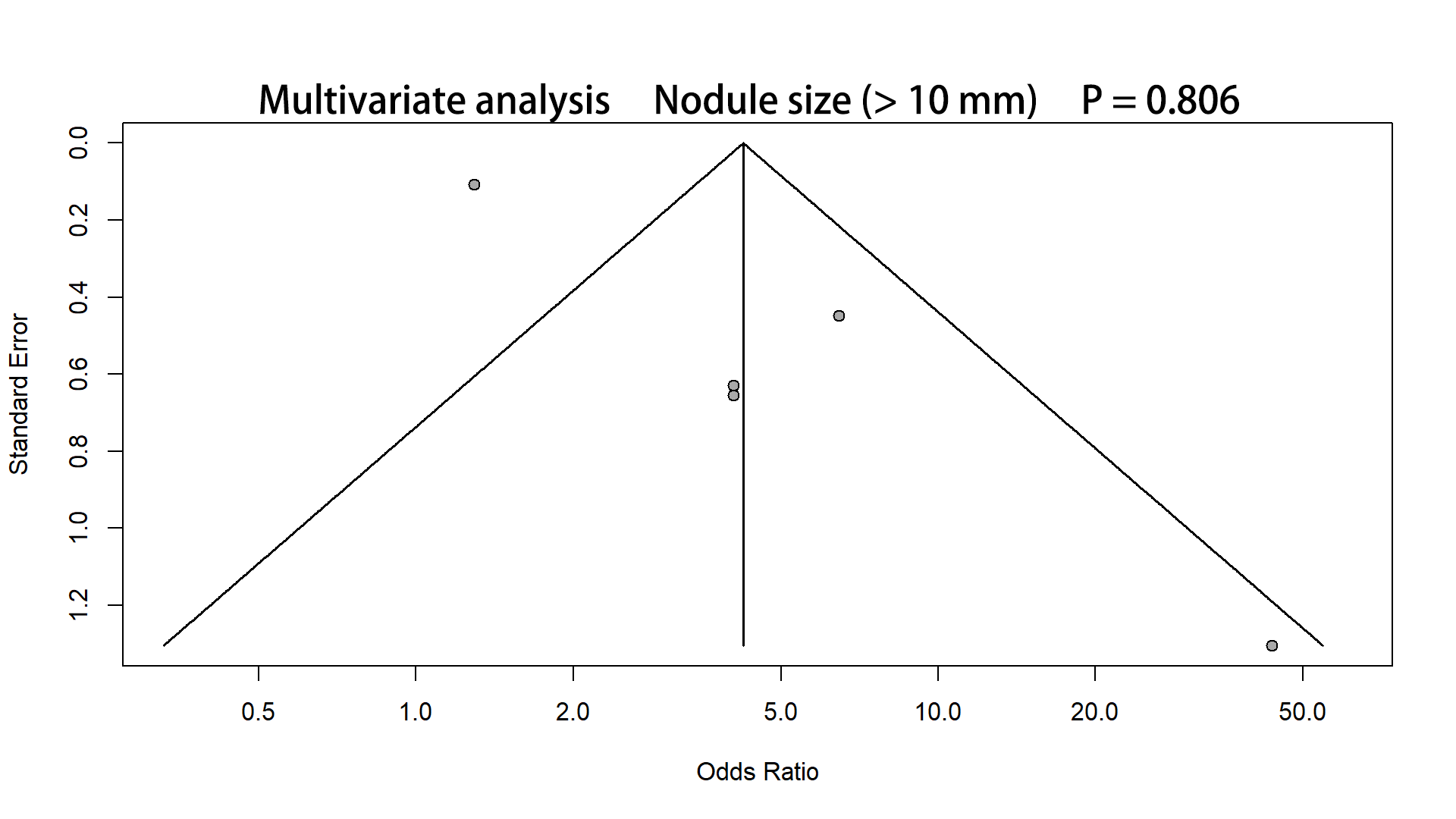

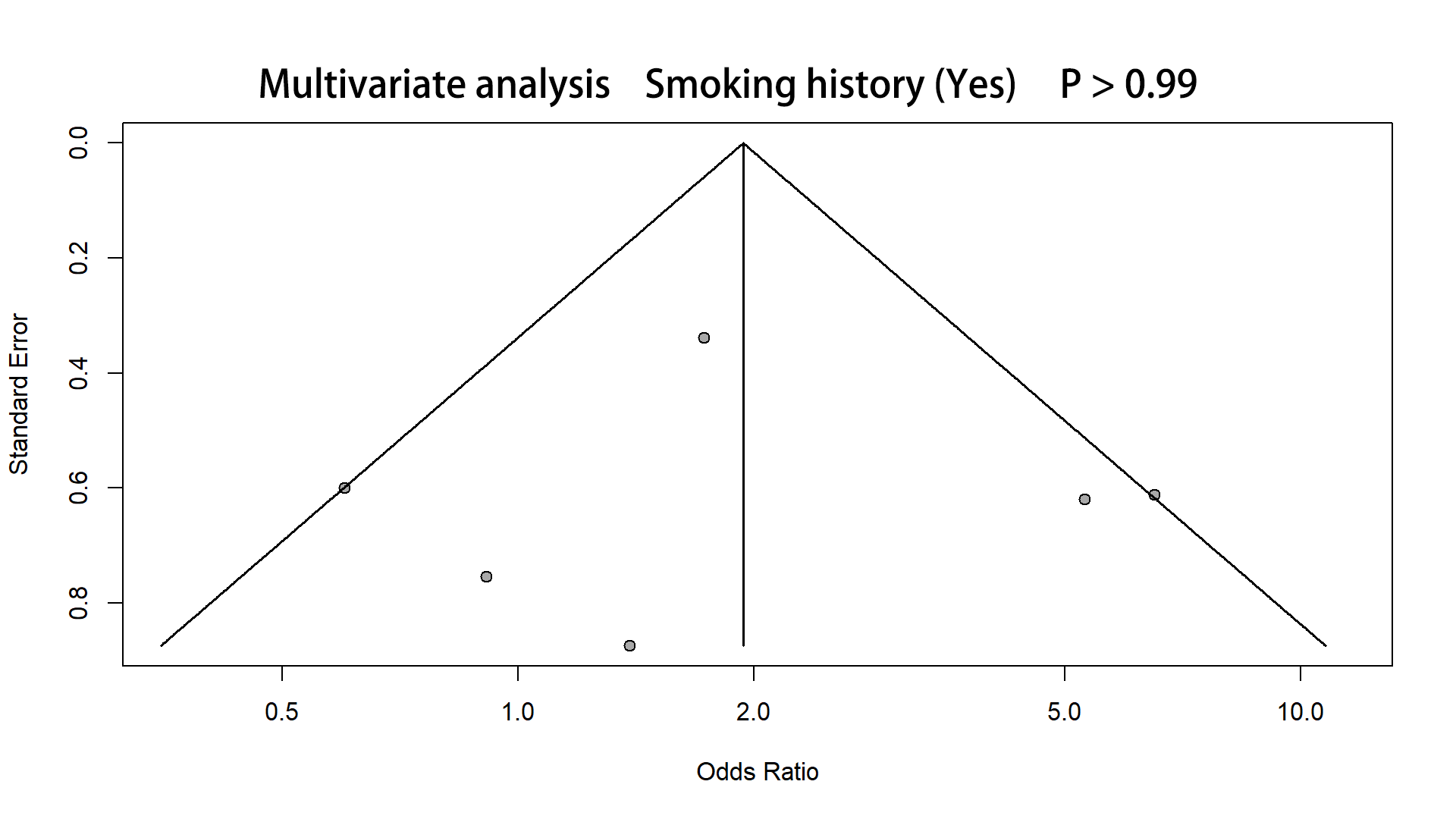

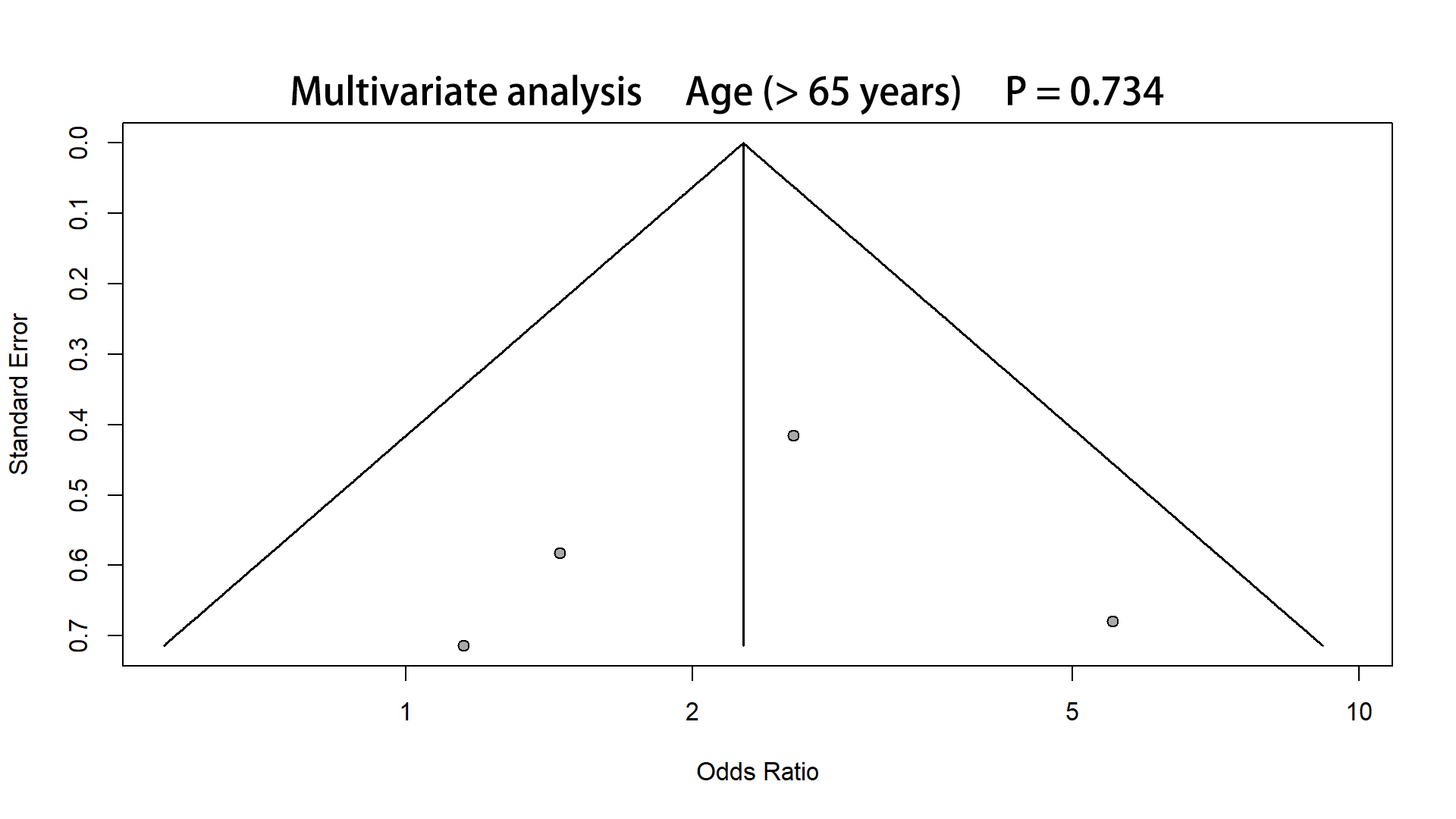

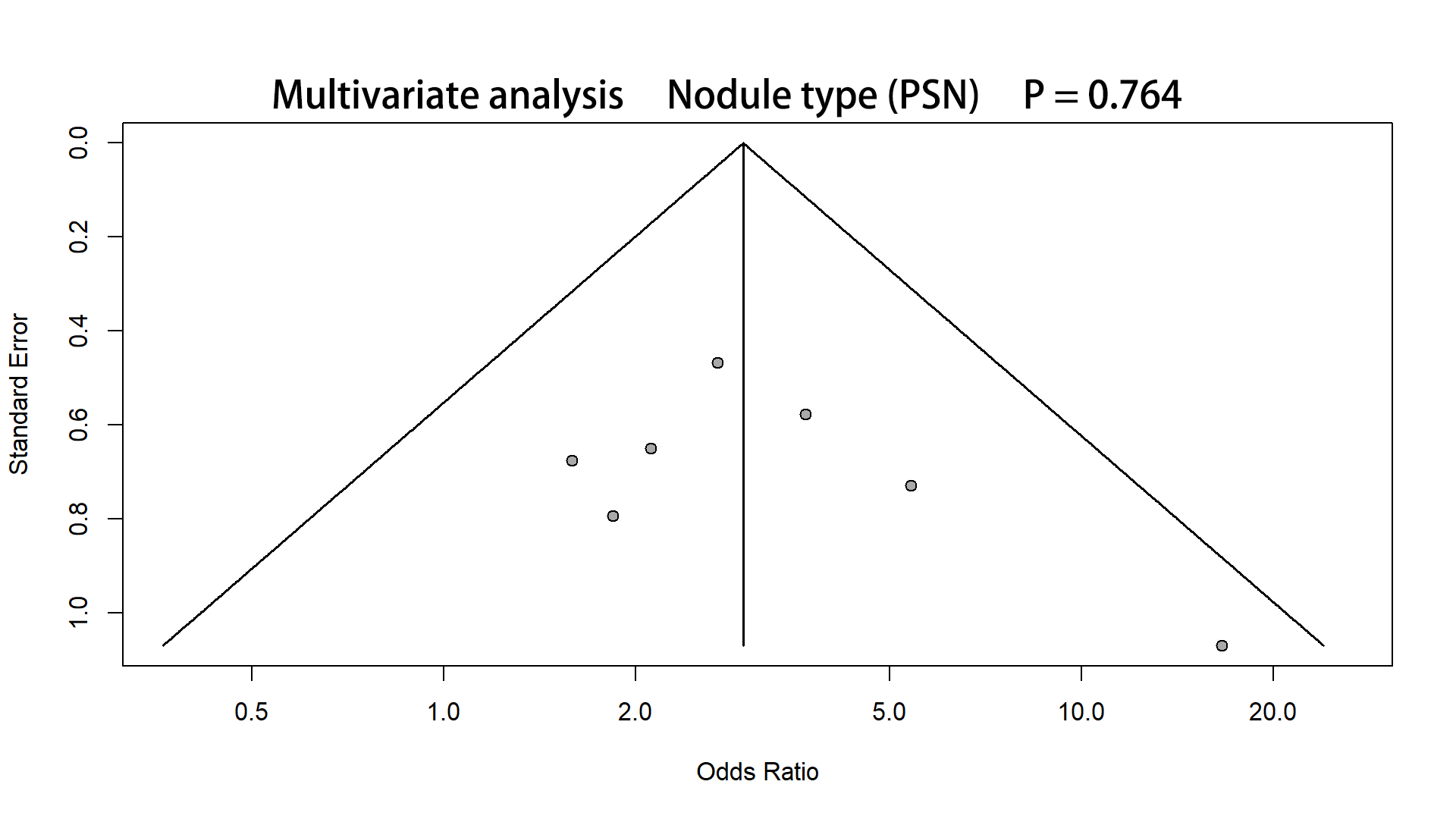


**Figure S3.** Funnel plots showed no publication bias in either analyses based on univariate analysis results or multivariate analysis results in SSN.


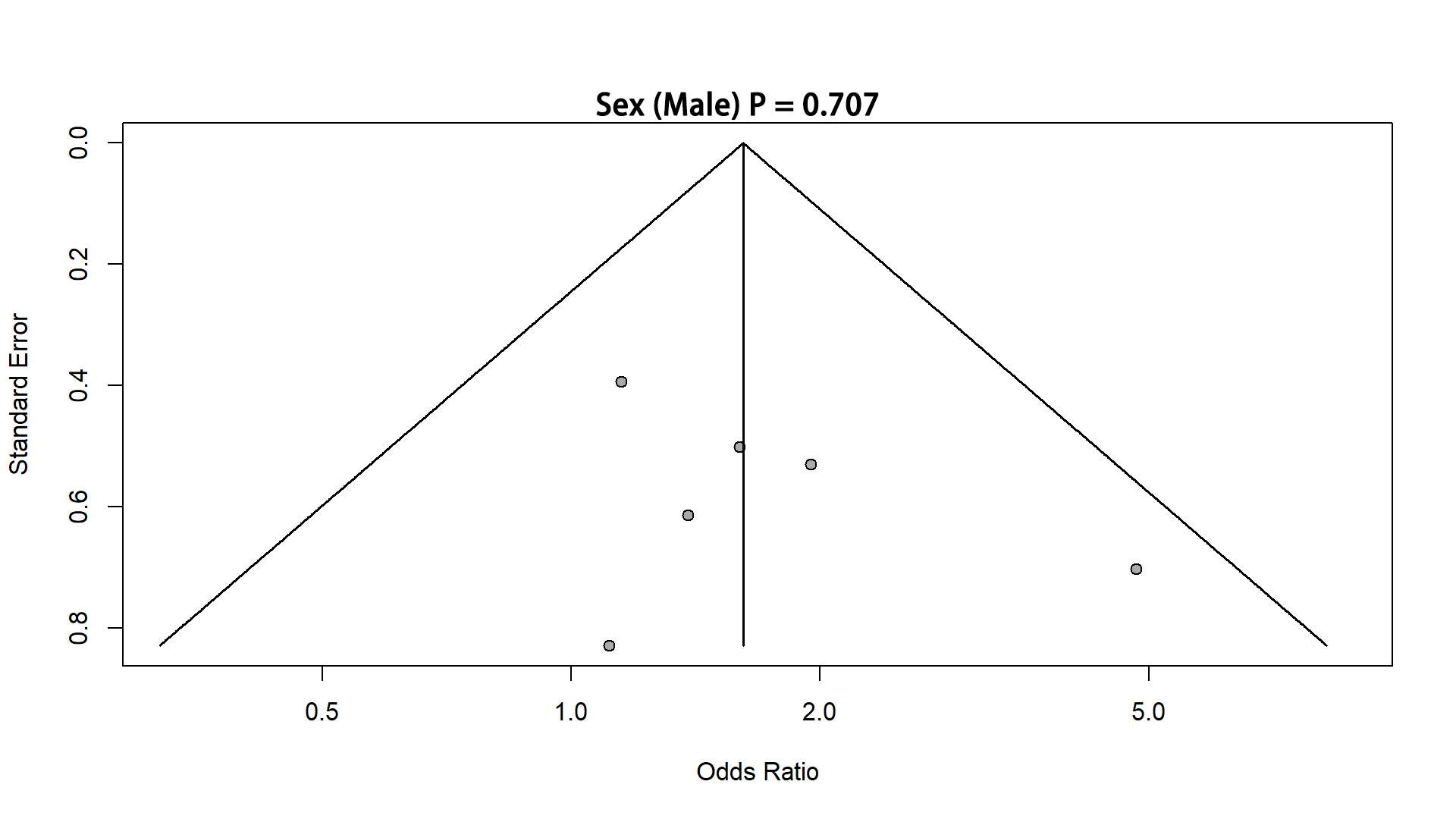

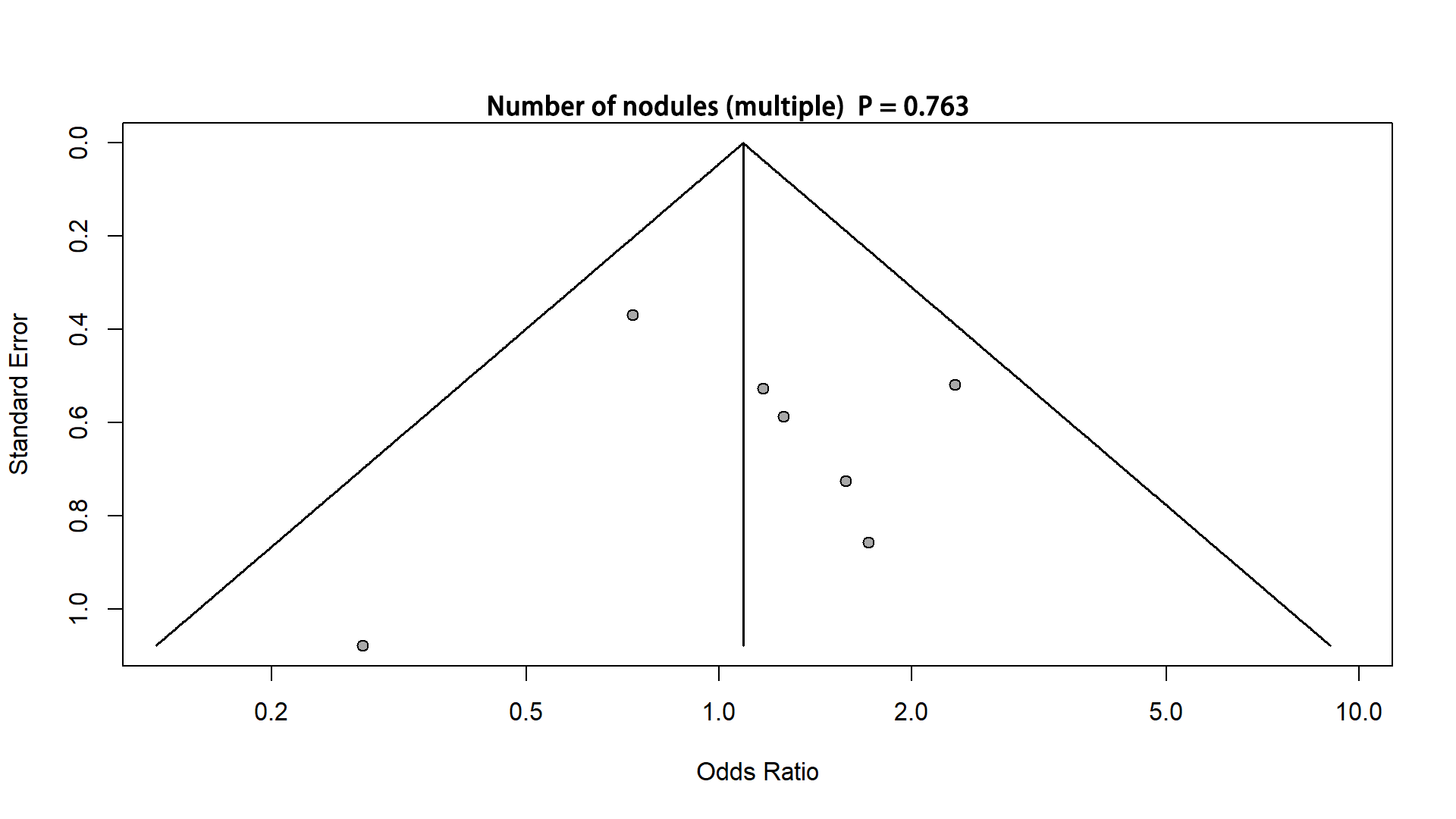

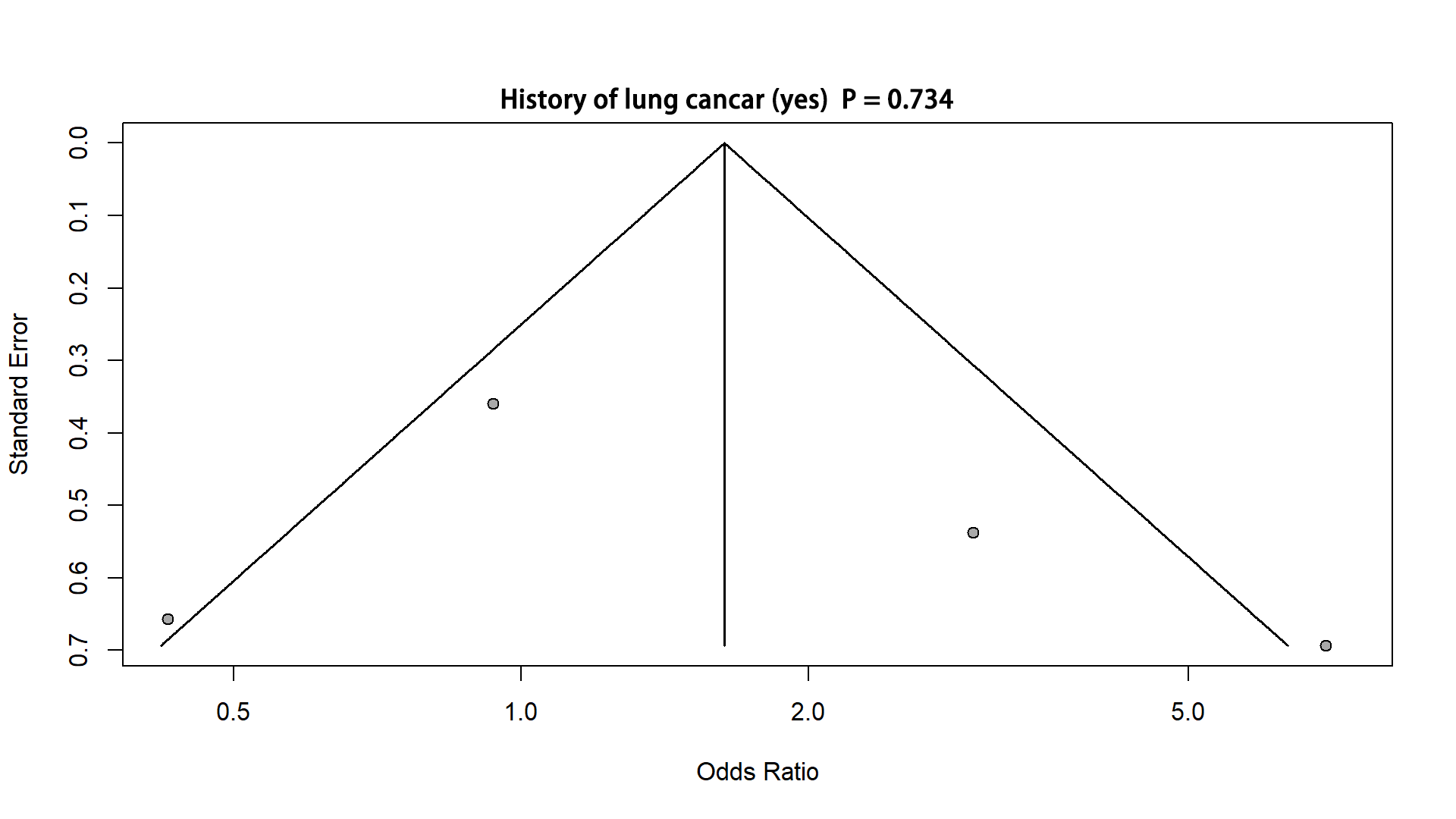

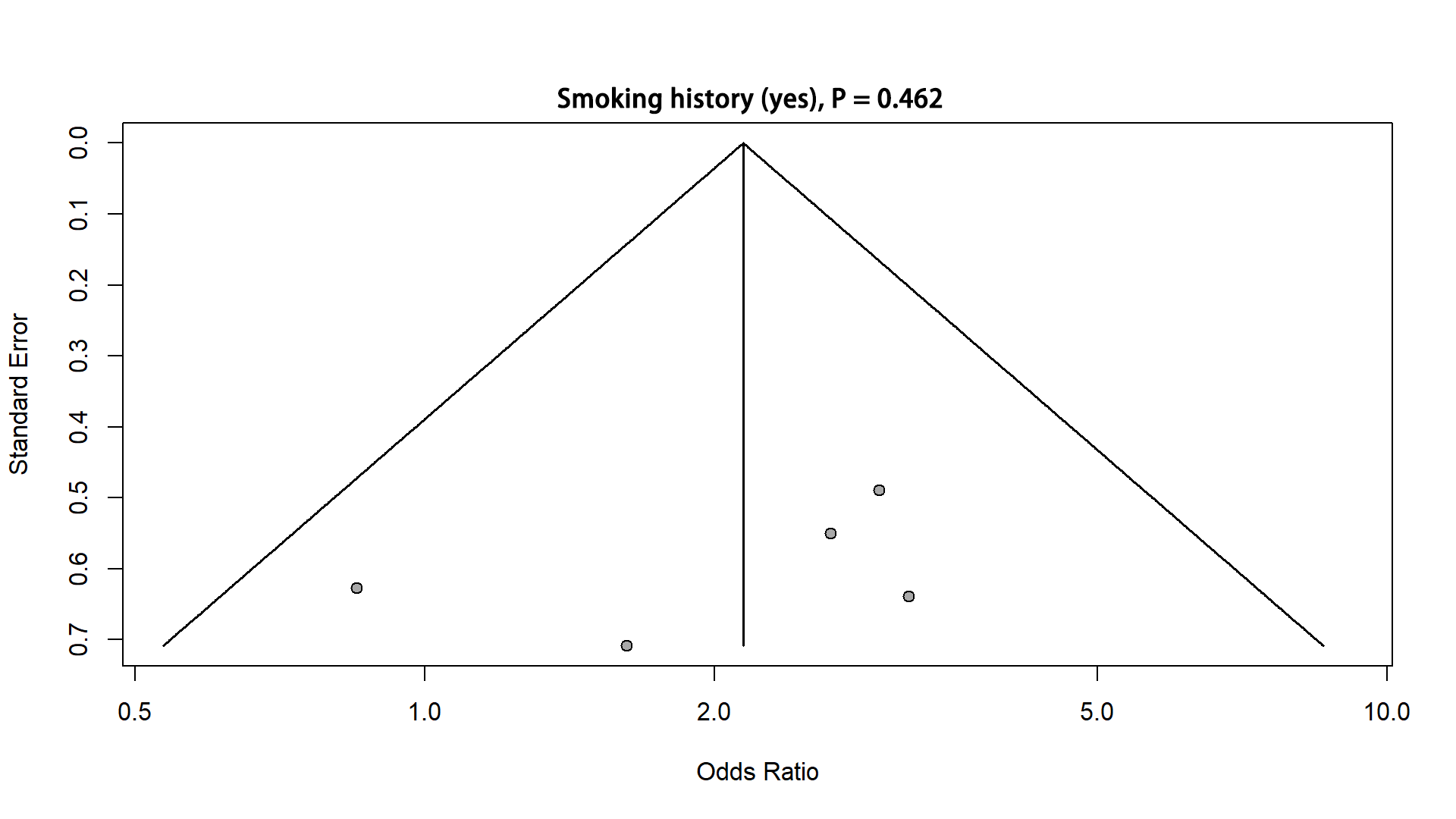

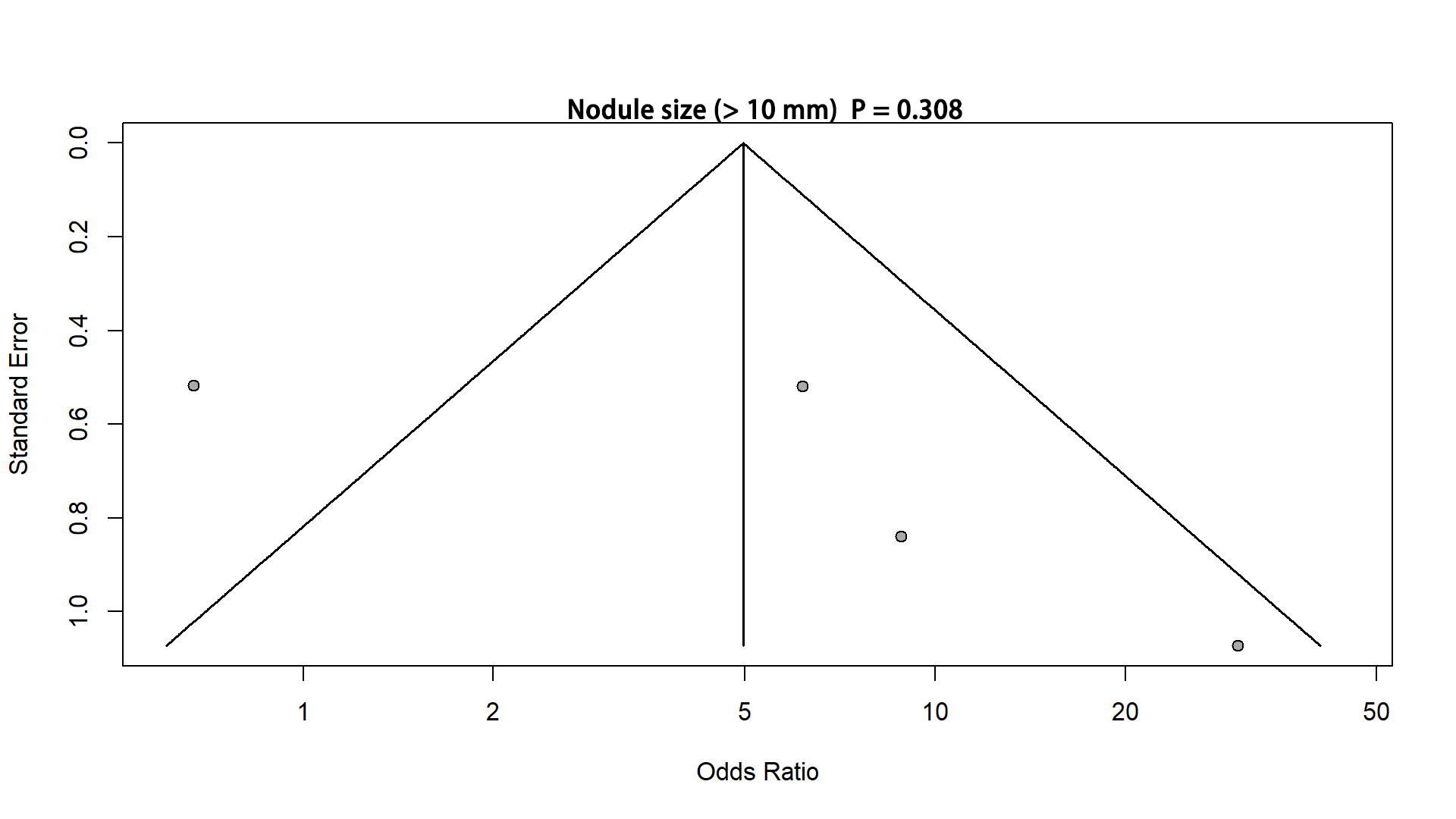

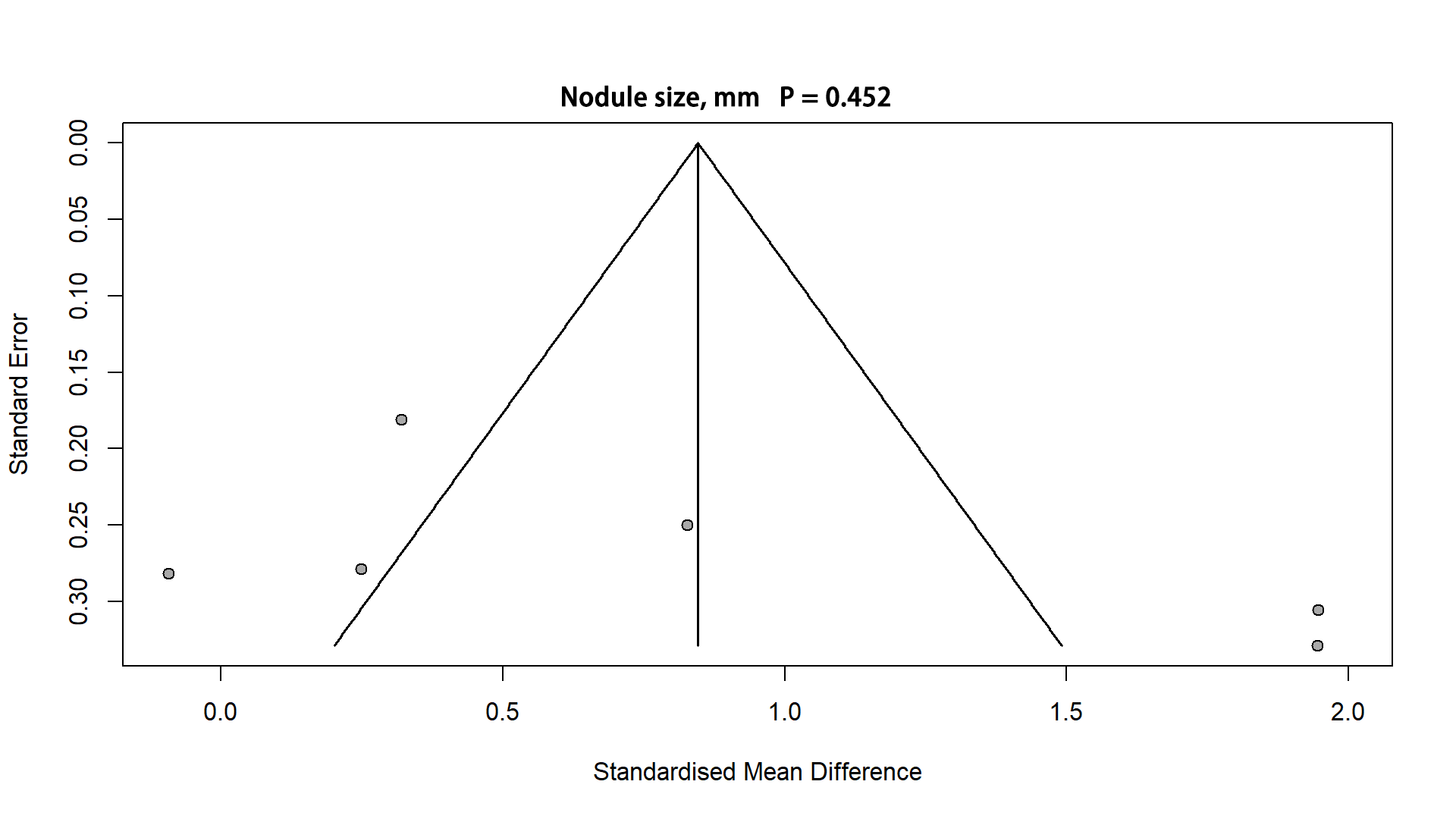

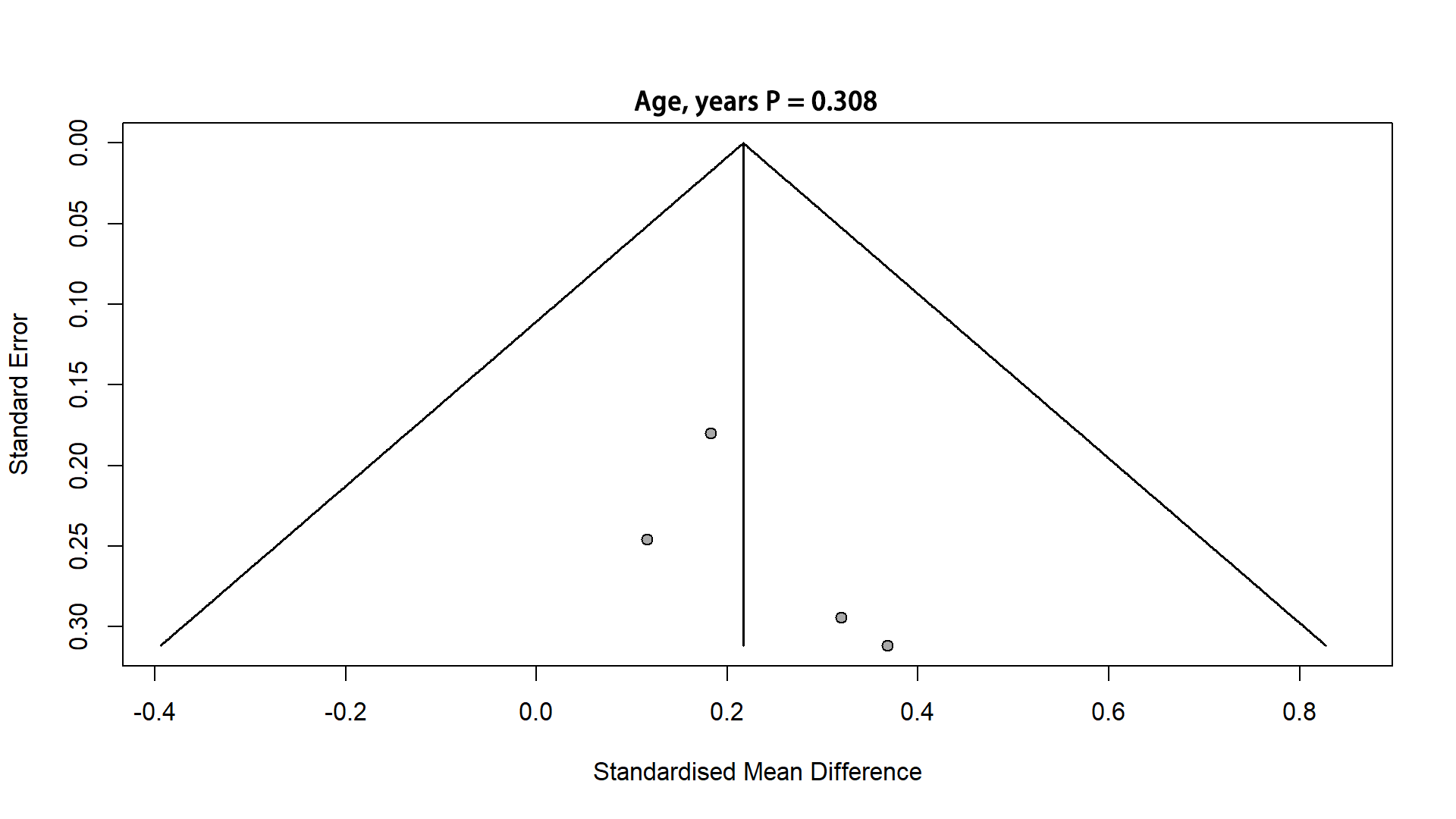

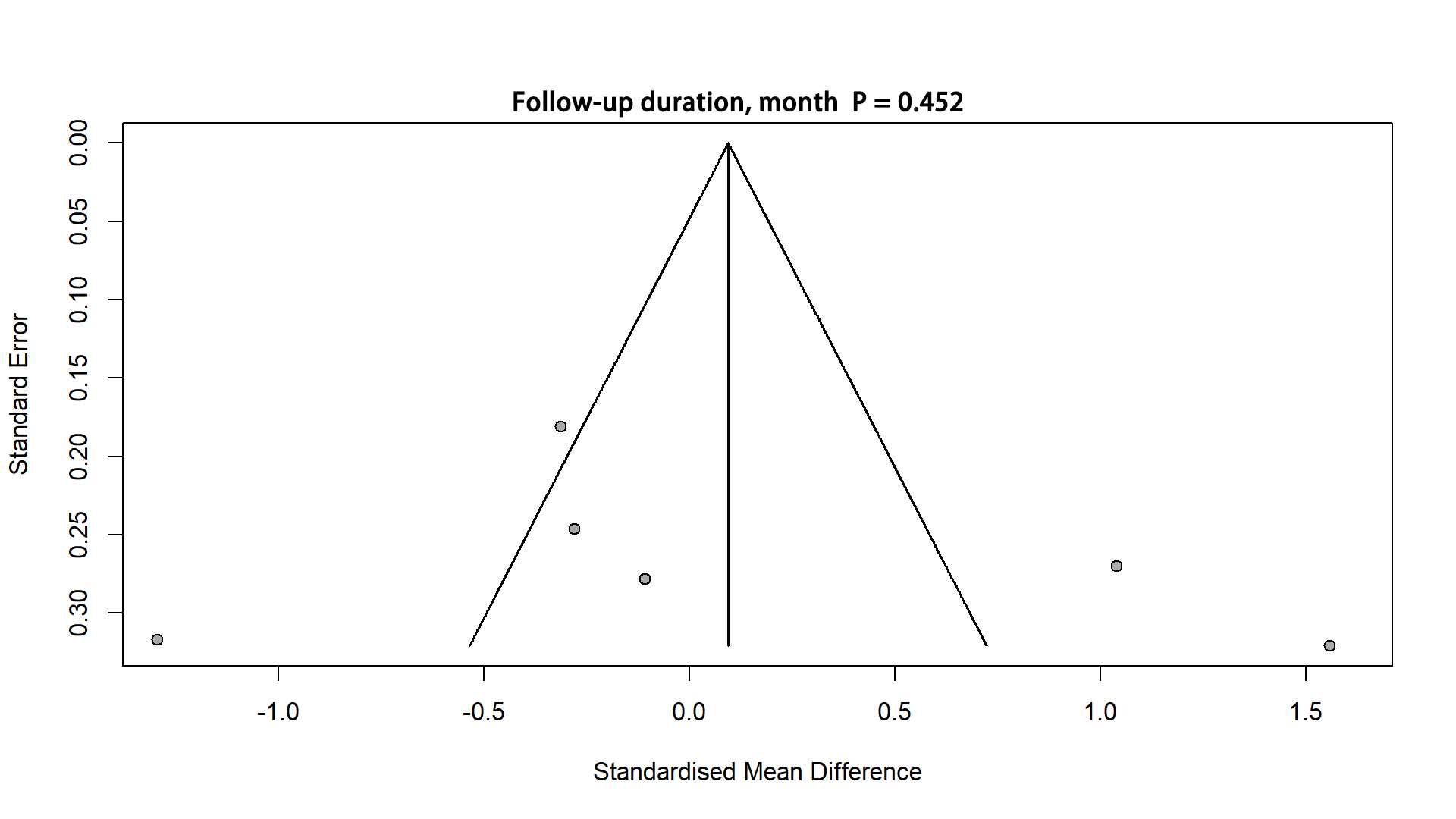

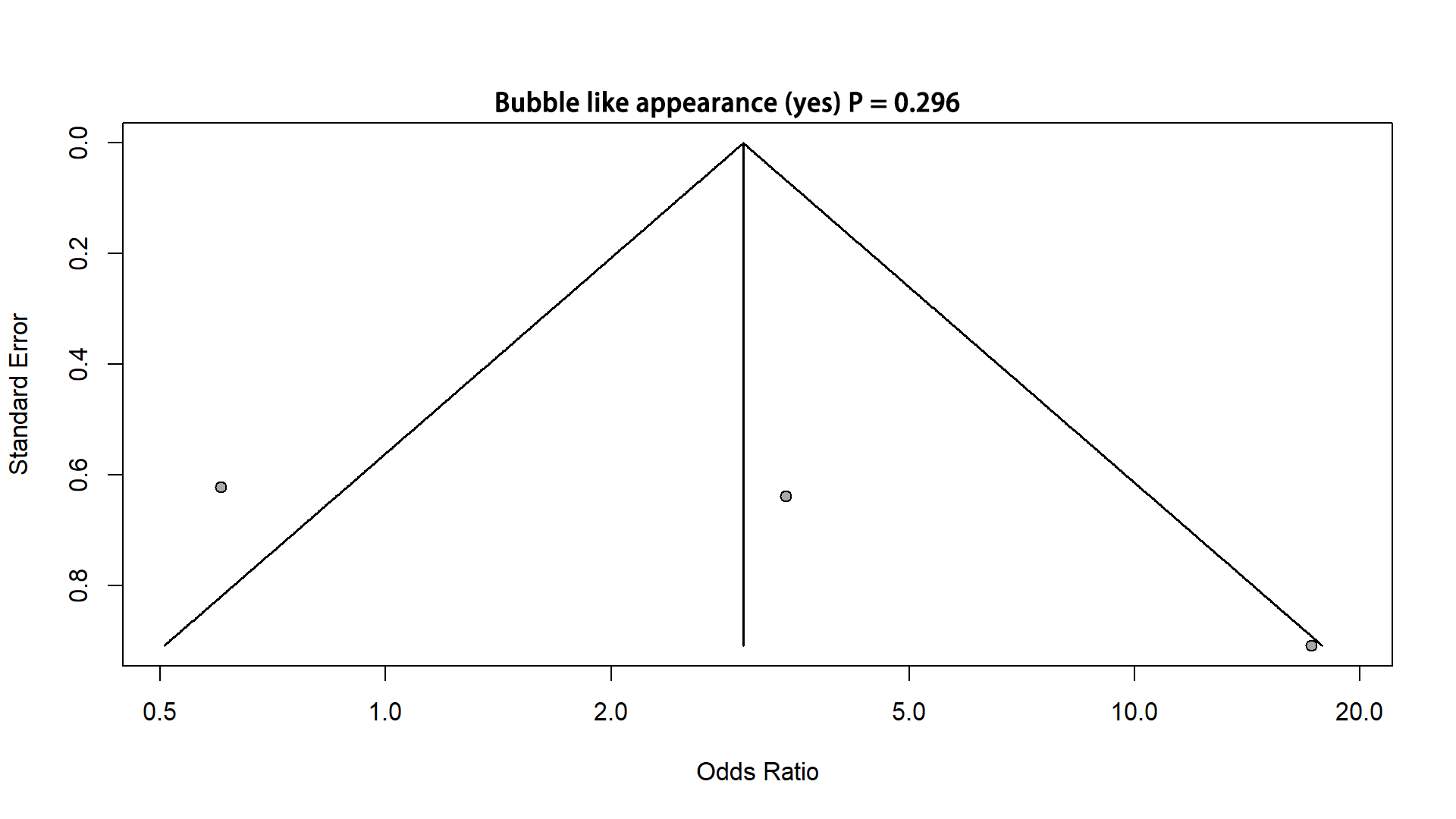

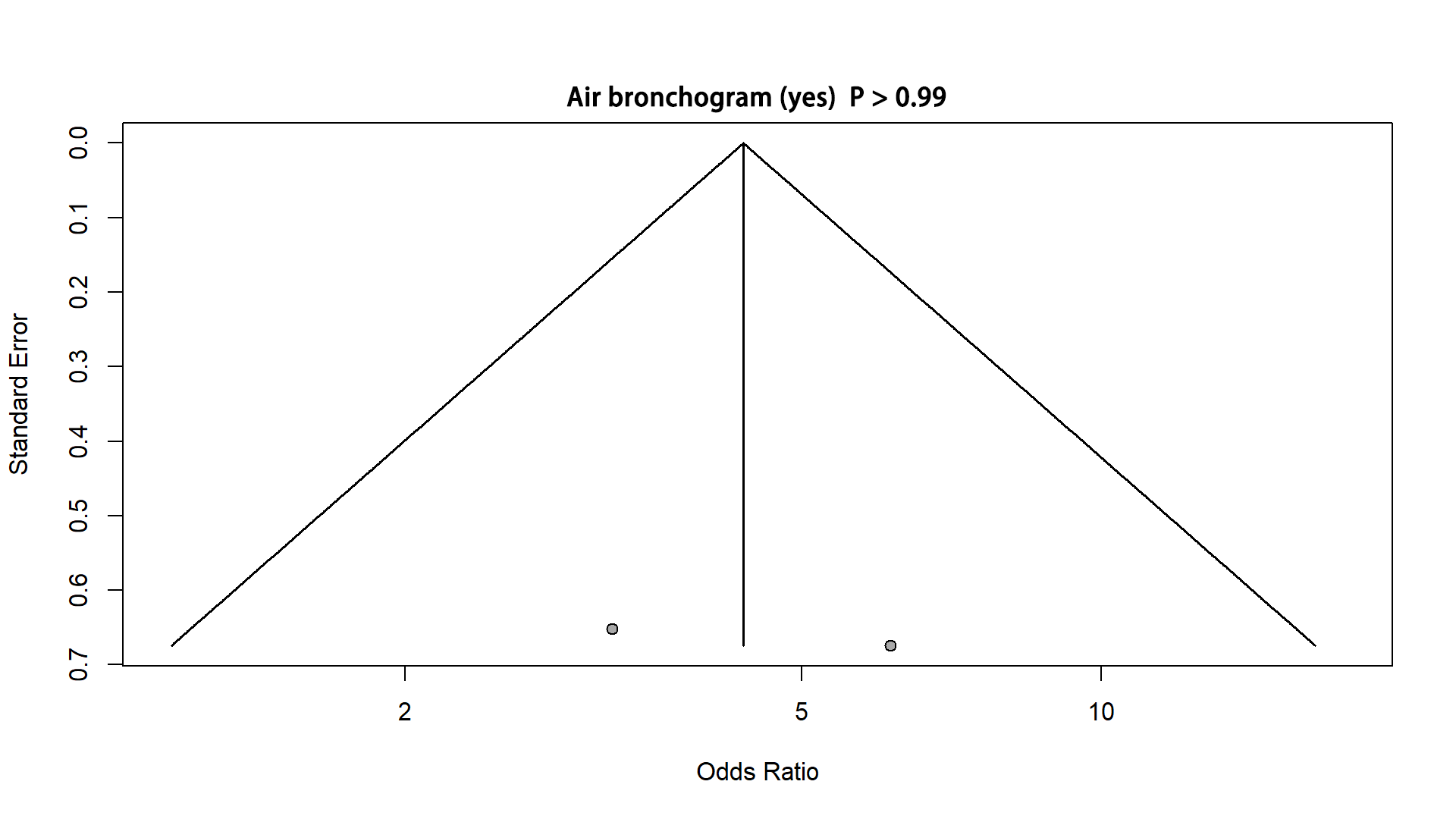

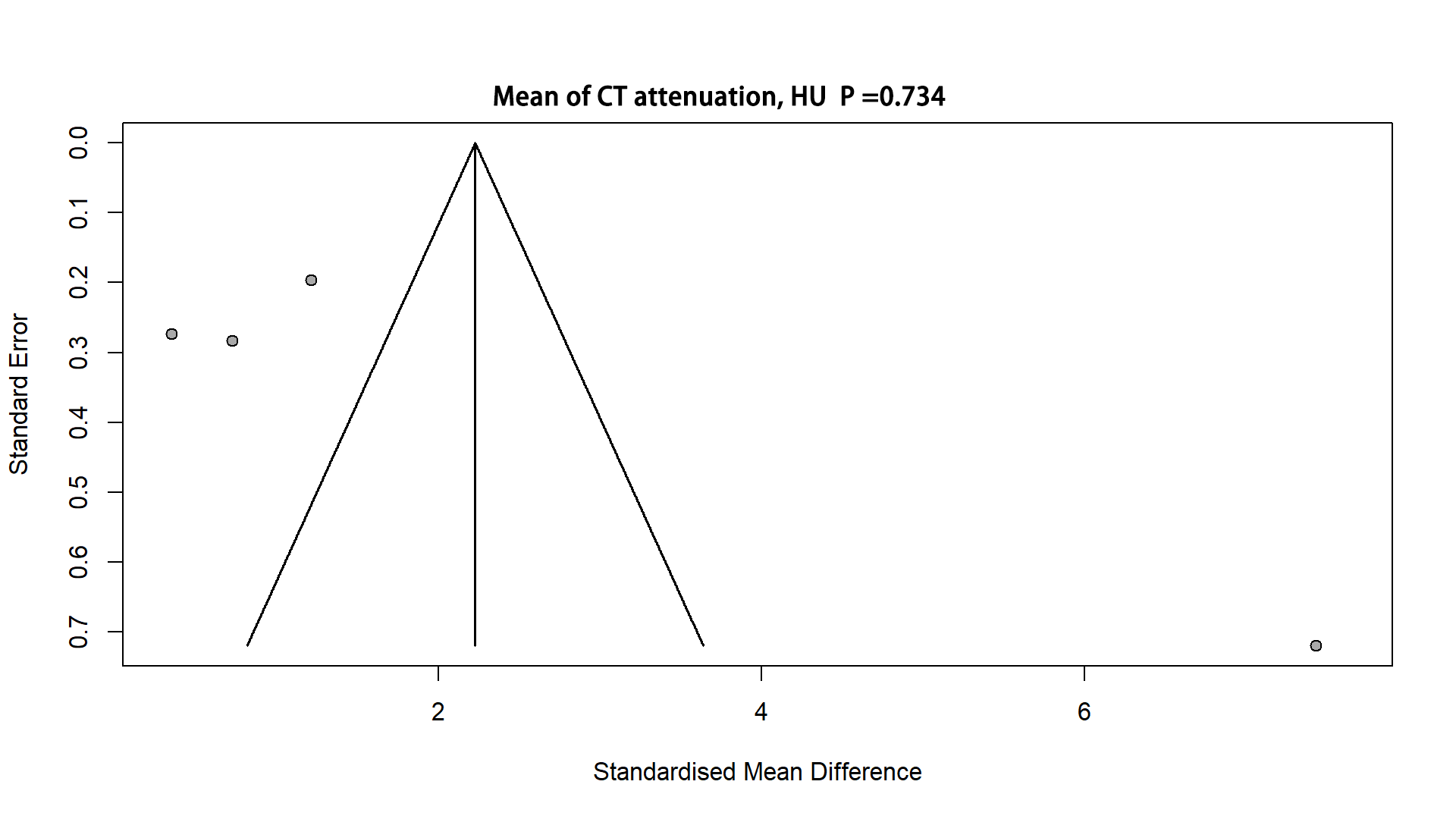

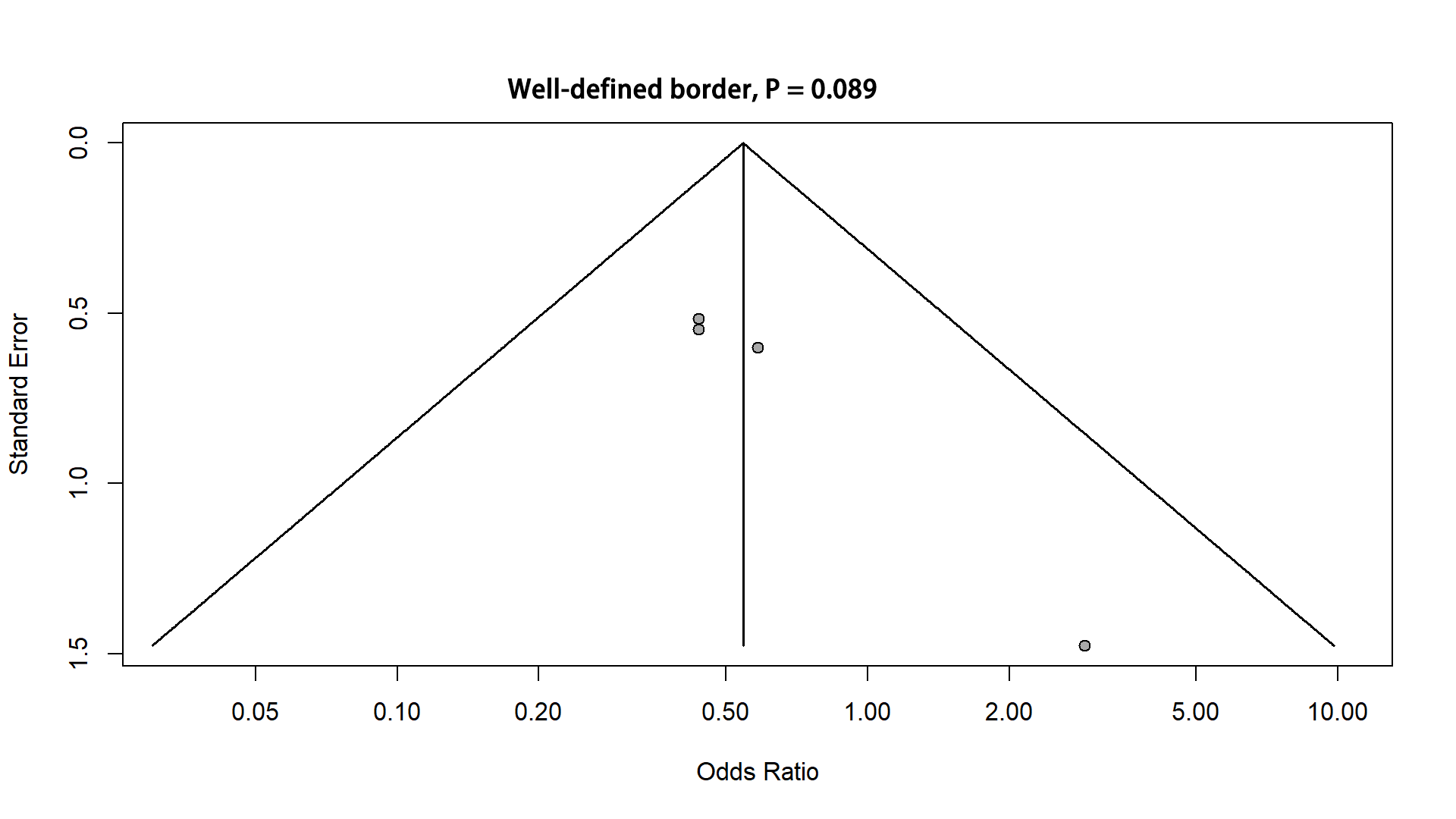

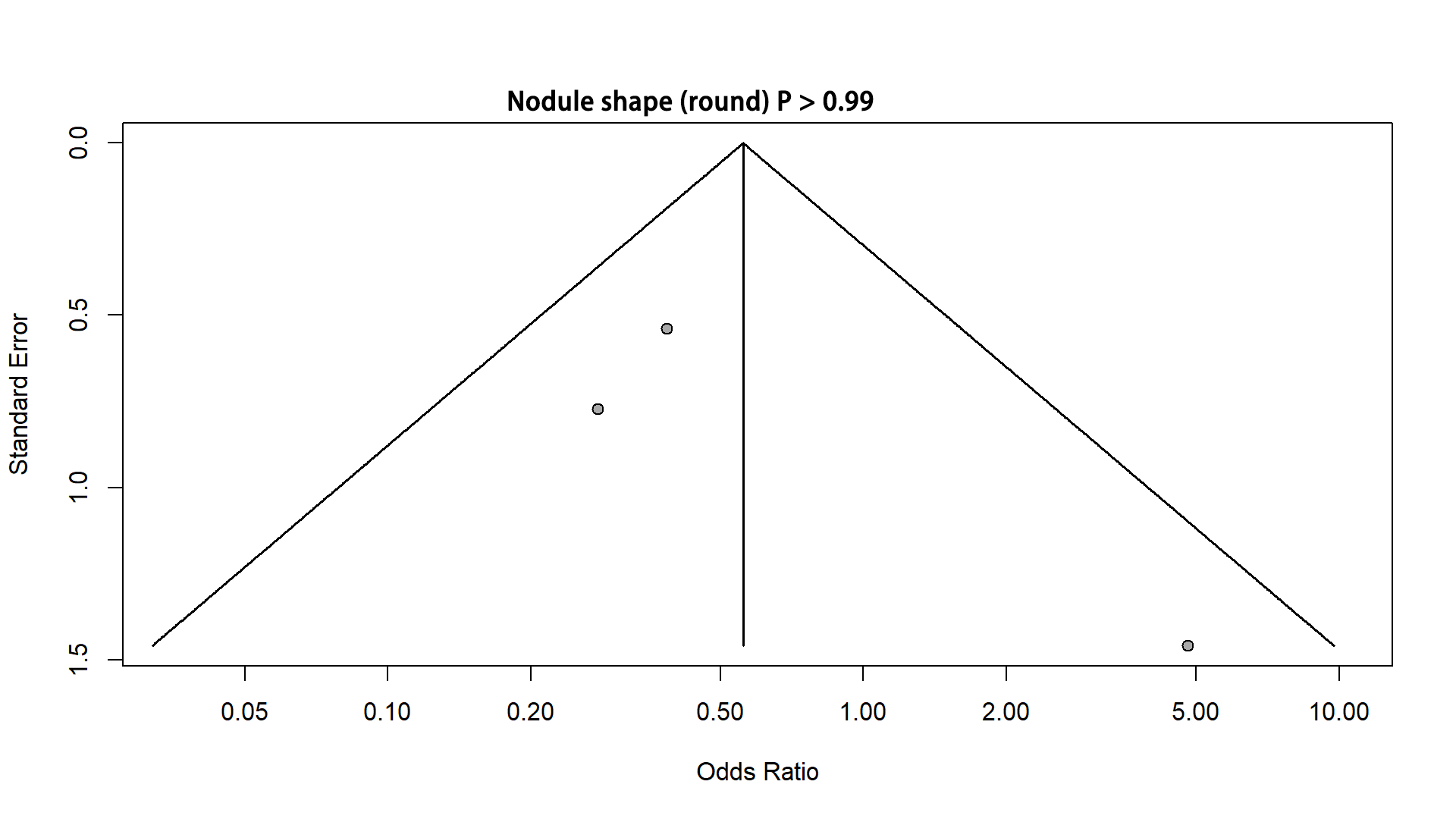

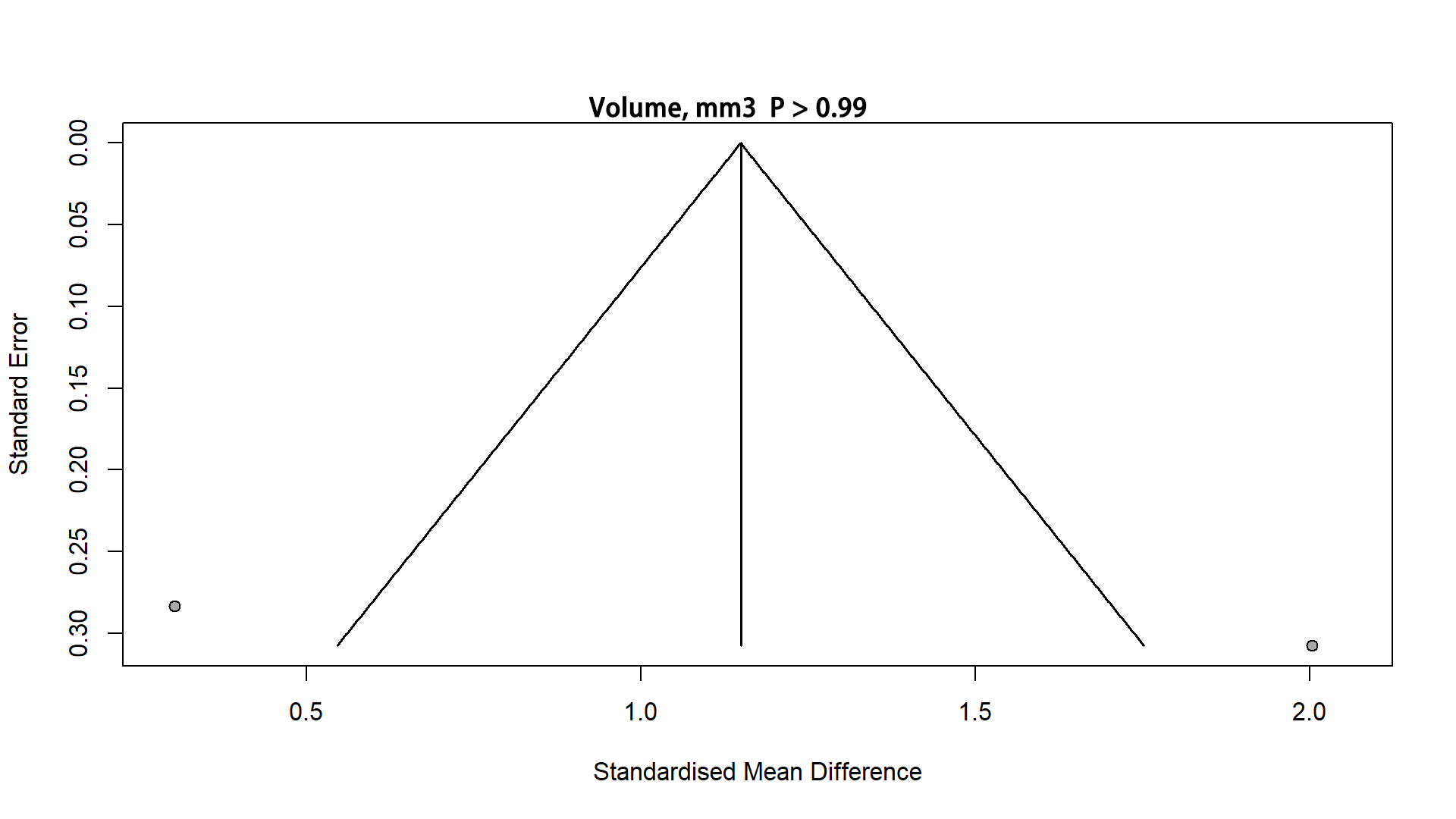

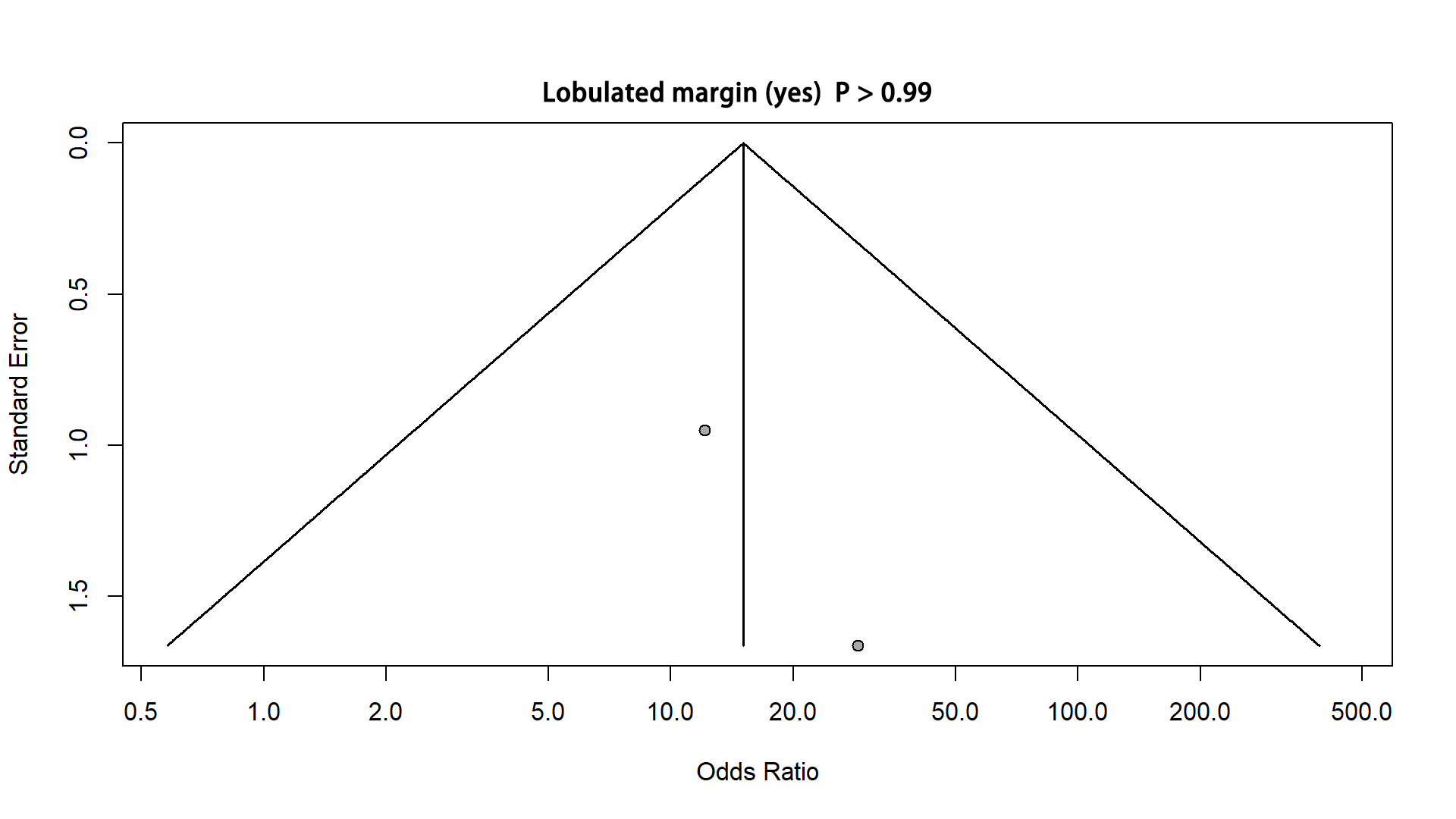

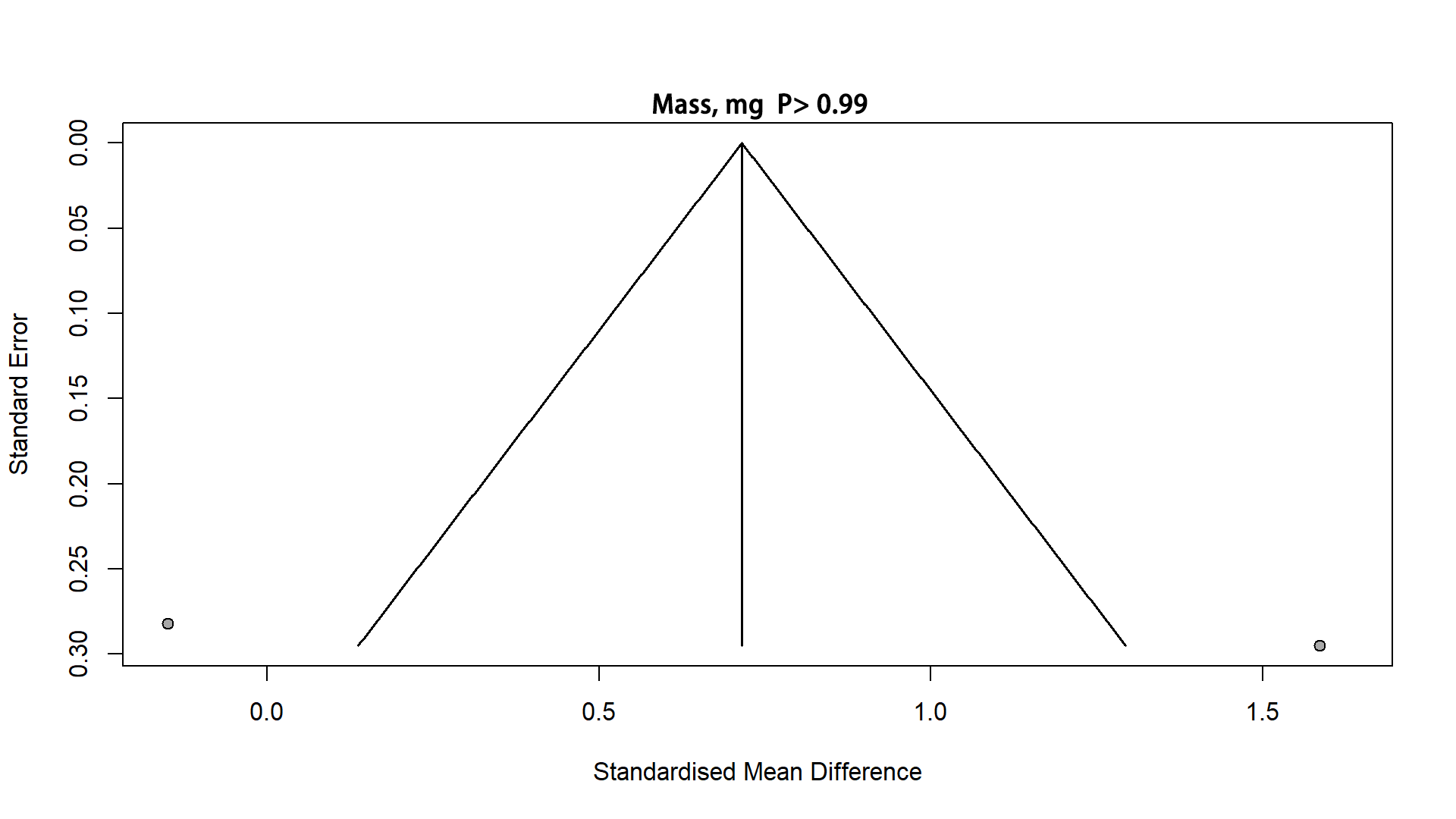


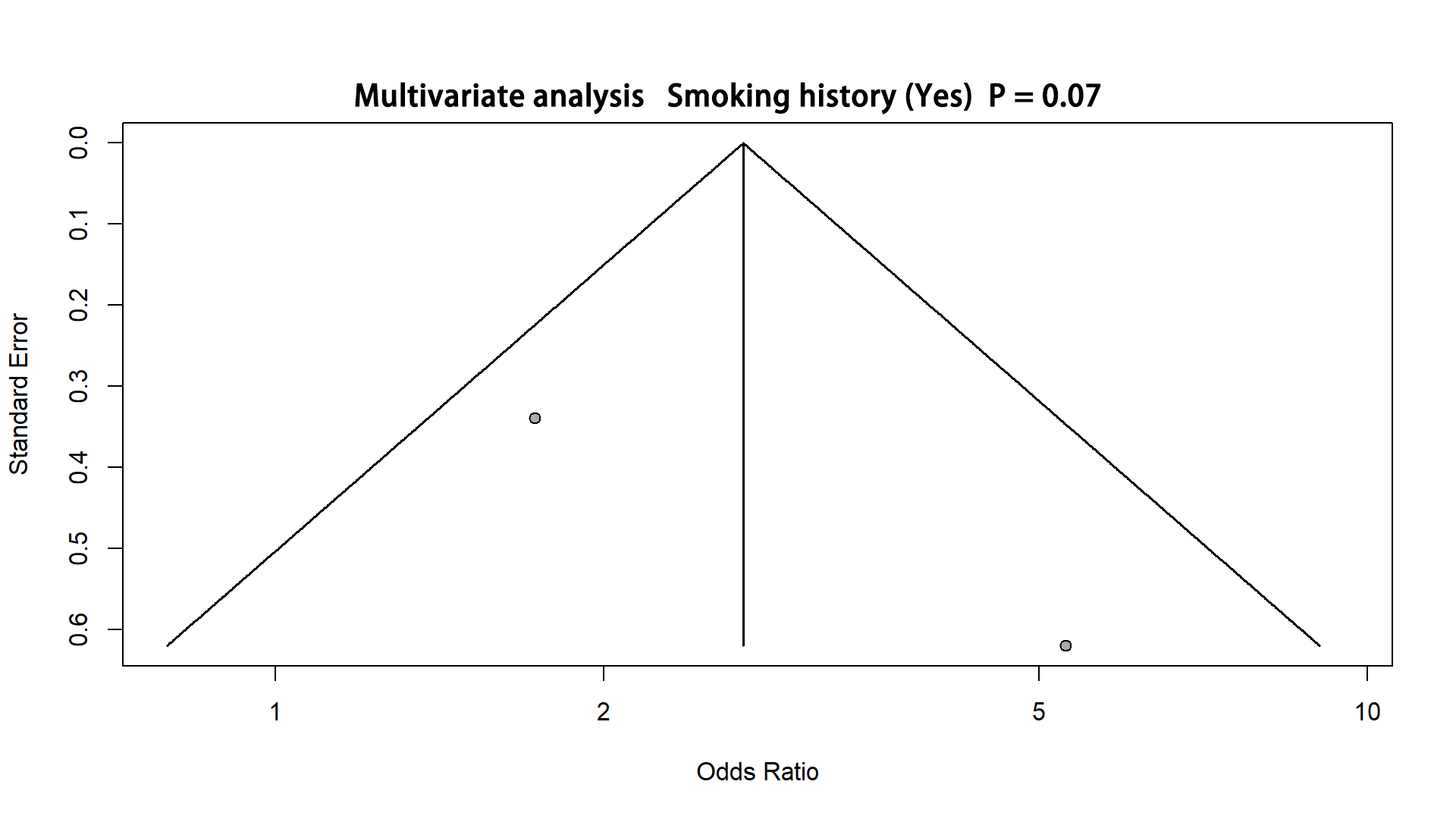


**Figure S4.** Funnel plots showed no publication bias in either analyses based on univariate analysis results or multivariate analysis results in pGGN.

**Part 2: Relationship between SSN growth and malignancy**

Of the 19 studies included in the meta-analysis, 8 studies state the pathological results of growth SSNs and stable SSNs respectively (Table S4). There are 293 growth SSNs and 1239 stable SSNs in the 8 studies. 179 (61.09%, 179/293) growth SSNs and 130 (10.49%, 130/1239) stable SSNs were surgically resected. Pathological categories for these 179 growth SSNs were precursor glandular lesion in 34(18.99%) and invasive lung adenocarcinoma in 145 (81.01%). Pathological categories for these 130 stable SSNs were precursor glandular lesion in 62 (47.69%), invasive lung adenocarcinoma in 64 (49.23%), and benign disease in 4 (3.08%).

**Table S4.** Pathological results of resected SSNs in the included studies.

| Study | Year |  | Growth SSN | | | | |  | Stable SSN | | | | |
| --- | --- | --- | --- | --- | --- | --- | --- | --- | --- | --- | --- | --- | --- |
|  |  |  | Total | Resected | Pathological results | | |  | Total | Resected | Pathological results | | |
|  |  |  |  |  | Benign | PGL | IAC |  |  |  | Benign | PGL | IAC |
| Takashi Eguchi | 2014 |  | 64 | 26 | 0 | 3 | 23 |  | 60 | 7 | 1 | 2 | 4 |
| Jaeyoung Cho | 2016 |  | 15 | 7 | 0 | 0 | 7 |  | 438 | 5 | 0 | 4 | 1 |
| Boksoon Chang | 2013 |  | 12 | 11 | 0 | 2 | 9 |  | 110 | 0 | 0 | 0 | 0 |
| Jong Hyuk Lee | 2016 |  | 42 | 27 | 0 | 19 | 8 |  | 171 | 31 | 0 | 20 | 11 |
| So Hyeon Bak | 2016 |  | 34 | 34 | 0 | 4 | 30 |  | 20 | 20 | 0 | 2 | 18 |
| En-Kuei Tang | 2019 |  | 60 | 39 | 0 | 1 | 38 |  | 68 | 23 | 0 | 4 | 19 |
| Bixiong Wang | 2017 |  | 61 | 34 | 0 | 5 | 29 |  | 142 | 38 | 3 | 28 | 7 |
| Jong Hyuk Lee | 2020 |  | 5 | 1 | 0 | 0 | 1 |  | 230 | 6 | 0 | 2 | 4 |
| Total | _ |  | 293 | 179 | 0 | 34 | 145 |  | 1239 | 130 | 4 | 62 | 64 |

Note: PGL, Precursor Glandular Lesions; IAC, Invasive adenocarcinoma.

Meta-analysis showed significant correlation between SSN growth and malignancy (P = 0.038). Growth SSNs showed a 4.32-fold higher probability of invasive adenocarcinoma than stable SSNs (Figure S5). Funnel plots showed no publication bias (Figure S6).


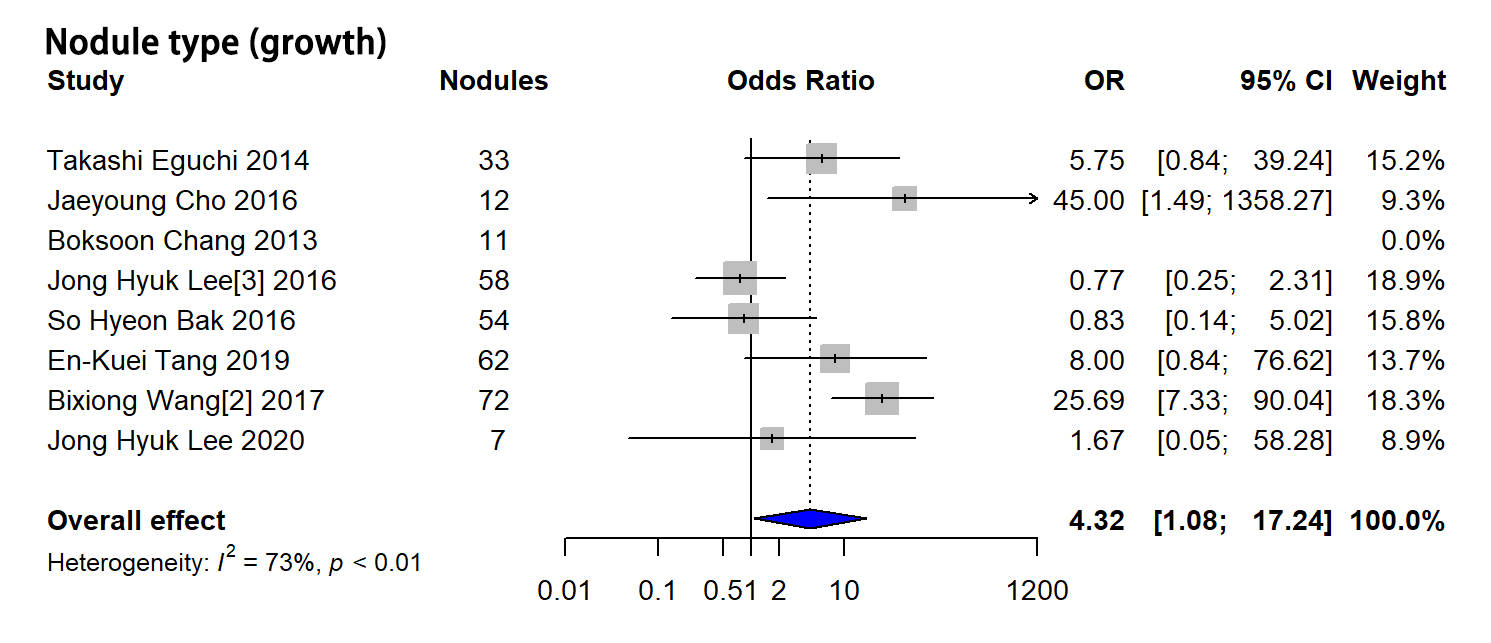
**Figure S5.** Forest plots showed significant correlation between nodule growth and malignancy. OR, odds ratio; CI, confidence interval.


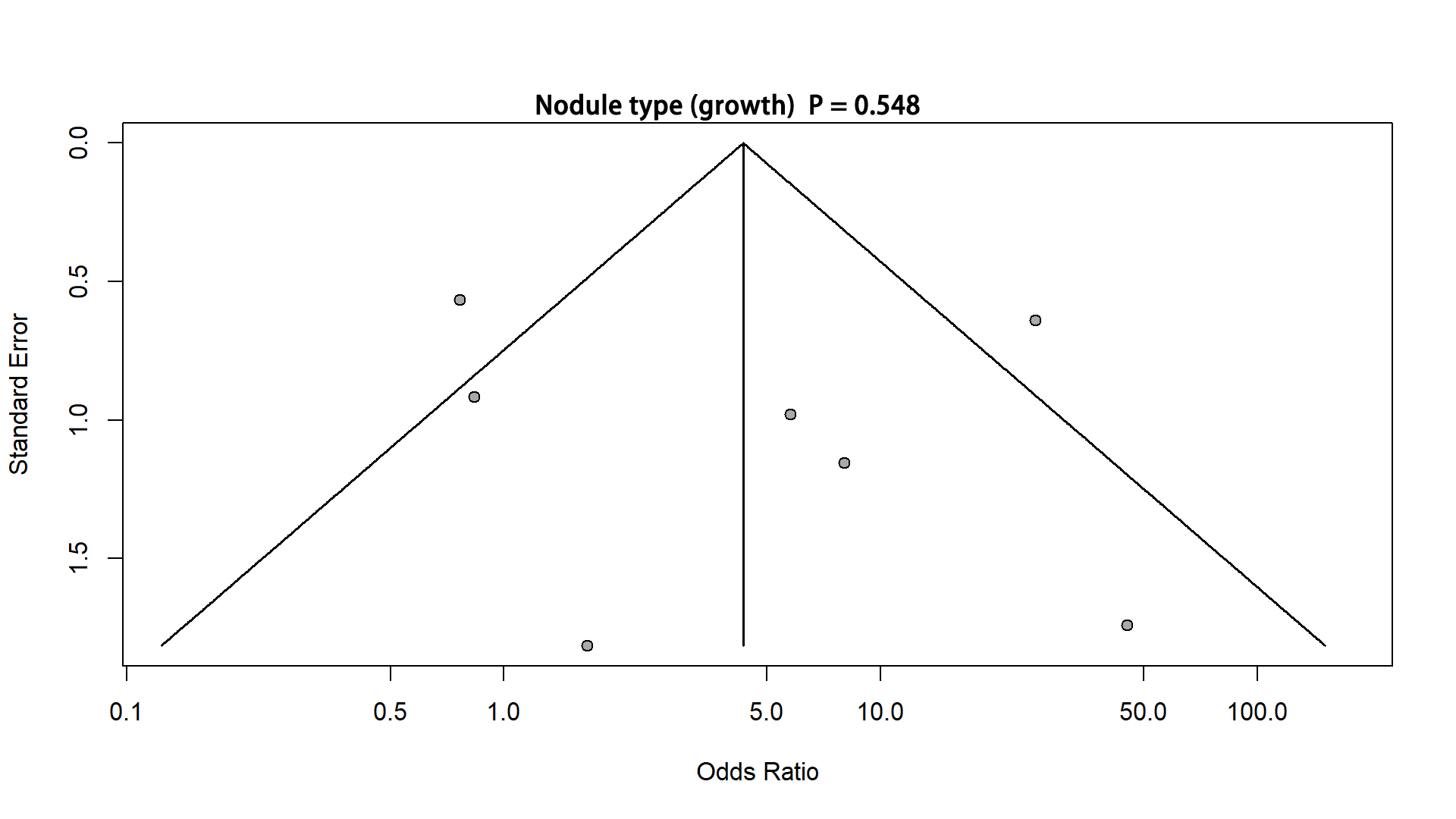


**Figure S6.** Funnel plots showed no publication bias (*p* = 0.548).
